# Asymmetric Synthesis and Biological Screening of Quinoxaline-Containing Synthetic Lipoxin A_4_ Mimetics (QNX-sLXms)

**DOI:** 10.1021/acs.jmedchem.1c00403

**Published:** 2021-06-17

**Authors:** Monica de Gaetano, Catherine Tighe, Kevin Gahan, Andrea Zanetti, Jianmin Chen, Justine Newson, Antonino Cacace, Mariam Marai, Andrew Gaffney, Eoin Brennan, Phillip Kantharidis, Mark E. Cooper, Xavier Leroy, Mauro Perretti, Derek Gilroy, Catherine Godson, Patrick J. Guiry

**Affiliations:** † School of Medicine, Diabetes Complications Research Centre, UCD Conway Institute, University College Dublin, Belfield, Dublin D04 N2E5, Ireland; ‡ Centre for Synthesis and Chemical Biology, School of Chemistry, UCD Conway Institute, University College Dublin, Belfield, Dublin D04 N2E5, Ireland; § William Harvey Research Institute, Queen Mary University London, London EC1M 6BQ, U.K.; ∥ Centre for Clinical Pharmacology, University College London, London WC1E 6JF, U.K.; ⊥ Department of Diabetes, Central Clinical School, Monash University, Melbourne, VIC 3004, Australia; # Domain Therapeutics SA, 67400 Strasbourg, Illkirch, France

## Abstract

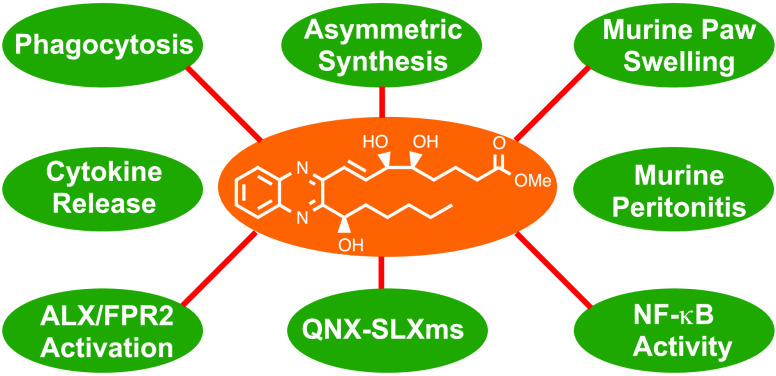

Failure to resolve inflammation underlies many prevalent pathologies. Recent insights have identified lipid mediators, typified by lipoxins (LXs), as drivers of inflammation resolution, suggesting potential therapeutic benefit. We report the asymmetric preparation of novel quinoxaline-containing synthetic-LXA_4_-mimetics (QNX-sLXms). Eight novel compounds were screened for their impact on inflammatory responses. Structure–activity relationship (SAR) studies showed that (*R*)-**6** (also referred to as AT-02-CT) was the most efficacious and potent anti-inflammatory compound of those tested. (*R*)-**6** significantly attenuated lipopolysaccharide (LPS)- and tumor-necrosis-factor-α (TNF-α)-induced NF-κB activity in monocytes and vascular smooth muscle cells. The molecular target of (*R*)-**6** was investigated. (*R*)-**6** activated the endogenous LX receptor formyl peptide receptor 2 (ALX/FPR2). The anti-inflammatory properties of (*R*)-**6** were further investigated *in vivo* in murine models of acute inflammation. Consistent with *in vitro* observations, (*R*)-**6** attenuated inflammatory responses. These results support the therapeutic potential of the lead QNX-sLXm (*R*)-**6** in the context of novel inflammatory regulators.

## Introduction

Inflammation is a vital physiological response to infection^1^ or trauma.^2^ Implicit in effective inflammation is a response limited in time and space and coupled to repair, which promotes return to homeostasis (catabasis).^3^ In contrast, unresolved chronic inflammation leads to fibrosis, tissue scarring, and, ultimately, organ failure.^4^ The resolution of inflammation is a prerequisite for homeostasis and tissue integrity maintenance,^5^ and it is now understood that chronic, insidious inflammation is an important driver of numerous prevalent conditions including arthritis,^6^ atherosclerosis, diabetes, and associated vascular complications.^7^ Efforts to repurpose existing drugs and to develop new ones for the treatment of such diseases are ongoing.^8^ To date, the focus has typically been on anti-inflammatory strategies^9^ and, while these show efficacy, there are challenges regarding the inevitable compromise of innate host defense strategies upon chronic administration.^10^

The LXs (an acronym for lipoxygenase interaction products) are endogenously generated eicosanoids originally isolated from human leukocytes.^11^ LX biosynthesis is initiated during the course of an inflammatory response,^12^ and LXs promote the resolution of inflammation by multiple convergent mechanisms, including inhibition of polymorphonuclear cell (PMN) chemotaxis, monocyte adhesion and transmigration,^13^ macrophage phagocytosis,^14^ and suppression of fibrosis.^15^ In addition to attenuating acute inflammatory responses, we have recently shown that LXs attenuate chronic inflammatory conditions, including renal fibrosis^16^ and the micro-^17^ and macrovascular^18^ complications of diabetes.^19^

Analysis of lipid mediator production over the course of self-limiting inflammation has led to the identification of additional families of mediators^20^ (including resolvins, protectins,^21^ and maresins^22^) with complementary bioactions leading to the collective term “specialized proresolving lipid mediators” (SPMs).^23^ Importantly, responses to SPMs are not coupled to compromised host defense.^24^ The discovery of SPMs^25^ and their bioactions and molecular targets^26^ has led to the proposal that these may be lead compounds for therapies based on promoting resolution.^27^

Deficits in the generation of endogenous LXs have been associated with several chronic inflammatory diseases, including asthma,^28^ arthritis,^29^ and cystic fibrosis.^30^ However, there are major obstacles to the application of LXs as pharmacological agents. LXA_4_ is rapidly metabolized *in vivo* by oxidation at C15, reduction of the C13–C14 double bond,^31^ ω-oxidation at C20,^32^ and β-oxidation at C-3.^12a^ LXA_4_ also has chemical stability issues, as it isomerizes to a mixture of double-bond isomers, including the corresponding *E*,*E*,*E*,*E*- or 11-*trans*-LXA_4_ in the presence of light, and decomposes in the presence of a strong acid.^12a^ Therefore, exploitation of the therapeutic potential of LXs has driven the design and synthesis of small molecules, collectively named “synthetic-LXA_4_-mimetics” (sLXms).^33^

**Figure 1:**
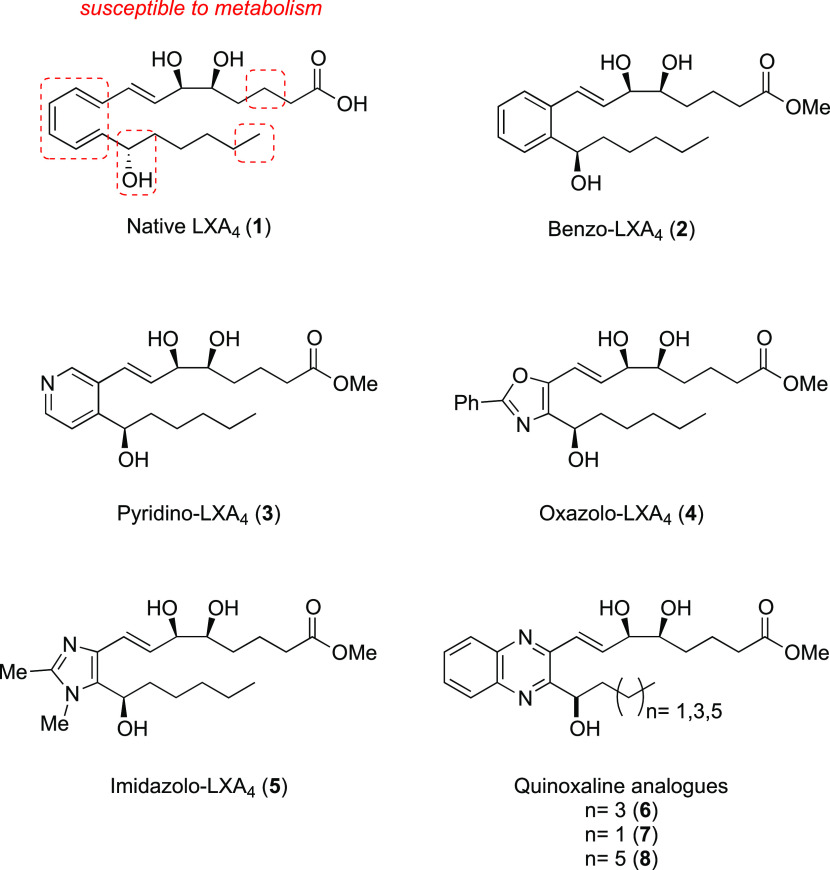
Native LXA_4_ (**1**) and synthetic-LXA_4_-mimetics (**2**–**8**).

Mimetics have been designed to retain key functional groups required for activity. Analogues with modifications to the top C1–8 chain have been reported and are more resistant to β-oxidation.^12a^ There are many analogues reported that have modified the lower C15–20 chain, which are equipotent or more potent than native LXA_4_ (**1**) but more resistant to C15 dehydrogenation and C20 oxidation.^33^ We have focused on modifying the triene core, replacing it with aromatic or heteroaromatic rings, to slow down enzymatic reduction of the C13–14 double bond and prevent double-bond isomerization. We reported the first asymmetric synthesis of the benzo-mimetic (**2**)^34^ and more recently the pyridino-,^35^ oxazolo-, and imidazolo-containing mimetics (**3–5**),^36^ all displaying similar bioactivity to LXA_4_ (**1**), with the imidazolo-mimetic proving to be the most potent, significantly attenuating lipopolysaccharide (LPS)-induced NF-κB activity and attenuating pivotal proinflammatory cytokine secretion.^36^ Here, we report the asymmetric preparation and biological evaluation of quinoxaline-containing LXA_4_ mimetics (QNX-sLXms) (**6**–**8**) (Figure 1).

These novel quinoxaline mimetics, like our current lead imidazolo-containing mimetic (**5**), possess two nitrogens in the heterocyclic system with enhanced potential to engage in hydrogen bonding with the receptor and potentially achieve greater potency than LXA_4_. Here, we describe the stereoselective preparation of both epimeric alcohols on the lower chain with small variations in lower alkyl chain length as well as our investigation to probe the impact of such modifications on biological activity. Biological activity has been assessed in the context of inflammatory responses, including NF-κB activity, cytokine release, and lactate dehydrogenase (LDH) secretion *in vitro*. Target receptor engagement (ALX/FPR2) has been investigated by determining intracellular calcium mobilization. The efficacy of the lead compound has been investigated in an *in vitro* model of phagocytosis as well as in murine models of acute peritonitis and paw swelling. The relative pharmacodynamic (PD) properties of the compounds have been compared to LXA_4_ in an effort to identify the lead compound. These analyses combined data from concentration–response curves (potency, efficacy, and slope) and identified compounds with enhanced potency to LXA_4_.

## Results

### Synthetic Chemistry

**Scheme 1:**
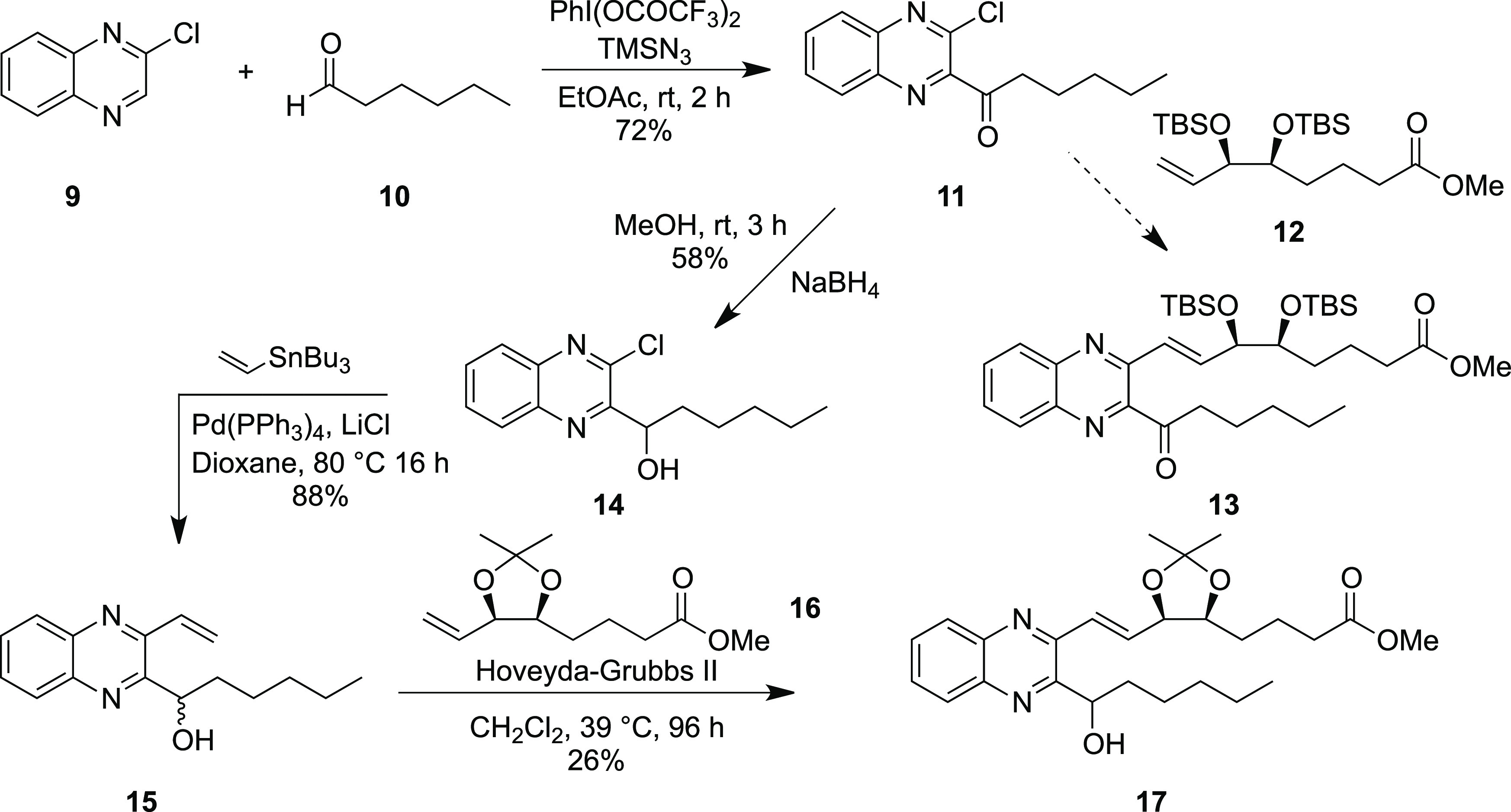
Synthesis of Coupling Partner **14** and Cross-Metathesis to Form **17**

The first synthetic route investigated for synthesizing the analogues was similar in approach to that used previously for the synthesis of the benzo-lipoxin mimetic (**2**). Starting with 2-chloroquinoxaline (**9**), ketone (**11**) was synthesized in 72% yield using Antonchick’s cross-dehydrogenative coupling procedure using hexanal (**10**) with (bis(trifluoroacetoxy)iodo)benzene and trimethylsilyl azide, Scheme 1.^37^ Attempts to lithiate (**9**) at the 1-position with LDA, LiTMP, and TMPMgCl–LiCl and a subsequent quench with various electrophiles were unsuccessful. Similarly, Minisci reactions under various conditions failed to furnish any of the desired product **11**. Our first attempts to form the quinoxaline to the alkene bond involved the Pd-catalyzed Heck reaction with terminal alkene **12**,^38^ but despite using a variety of palladium sources and ligands, the desired product **13** was not formed. A test reaction using methyl acrylate was carried out, and although the product was isolated in low yields, this indicated that the quinoxaline component was undergoing oxidative addition, but there was a problem with the low reactivity of the alkene reactant. Therefore, a new route to the analogues was developed invoking a Grubbs cross-metathesis to form the *trans*-alkene motif. The vinyl quinoxaline intermediate **15** was synthesized in two steps, by first reducing ketone **11** using sodium borohydride in 58% yield and then performing a Pd-catalyzed Stille coupling with tributylvinyltin, which proceeded in an 88% yield. We found that a direct Stille coupling on the quinoxaline ketone **11** led to an unstable product, which rapidly decomposed with similar instability issues of similar compounds having been previously reported.^39^ A Grubbs cross-metathesis was carried out employing the Hoveyda–Grubbs 2nd generation catalyst, and product **17** was stereoselectively obtained in a low yield of 26%, primarily due to difficulties with product purification. This limits the applicability of this route to scaling to bulk synthesis levels.

**Scheme 2:**
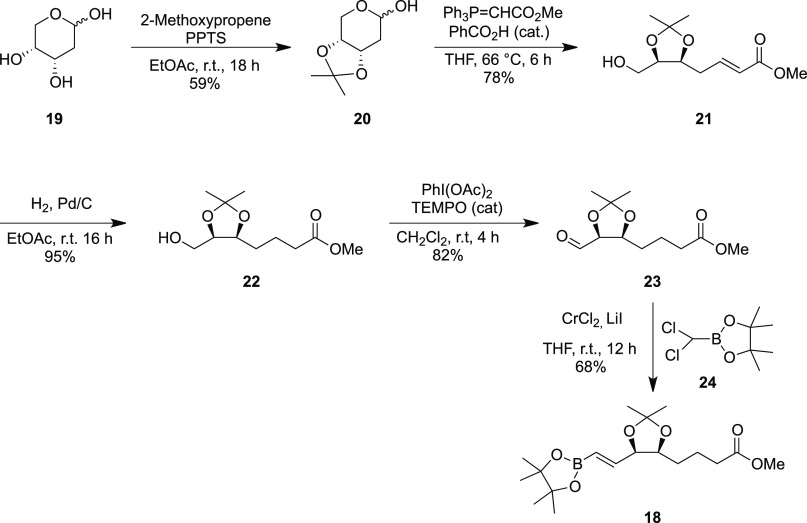
Synthesis of Vinyl Boronate **18**

An alternative route to the formation of mimetics of types **6–8** was investigated, this time featuring a Suzuki cross-coupling reaction as the key arene to alkene-bond-forming reaction, coupling the quinoxaline component **14** to the boronic ester **18**. The synthesis of the boronic ester coupling partner **18** has been recently reported by us, but the combined low yielding nature of the last two steps, a Seyferth–Gilbert homologation (68%) and a hydroboration using pinacolborane catalyzed by Schwartz’s reagent (34%), was limiting.^36^ Starting from commercially available 2-deoxy-d-ribose **19**, which has the desired stereochemistry in place, an acetonide protection to form **20** was carried out followed by a Wittig reaction and alkene reduction using Pd/C to prepare intermediate **22** (Scheme 2). The formation of the aldehyde **23** was optimized using a 2,2,6,6-tetramethylpiperidine 1-oxyl radical (TEMPO)-catalyzed oxidation, which proceeded in a 74% yield. A Takai reaction with the dichloromethyl pinacol boronate reagent **24**,^40^ chromium chloride, and lithium iodide proceeded to generate the boronate coupling partner **18** in a satisfactory 68% yield.

**Scheme 3:**
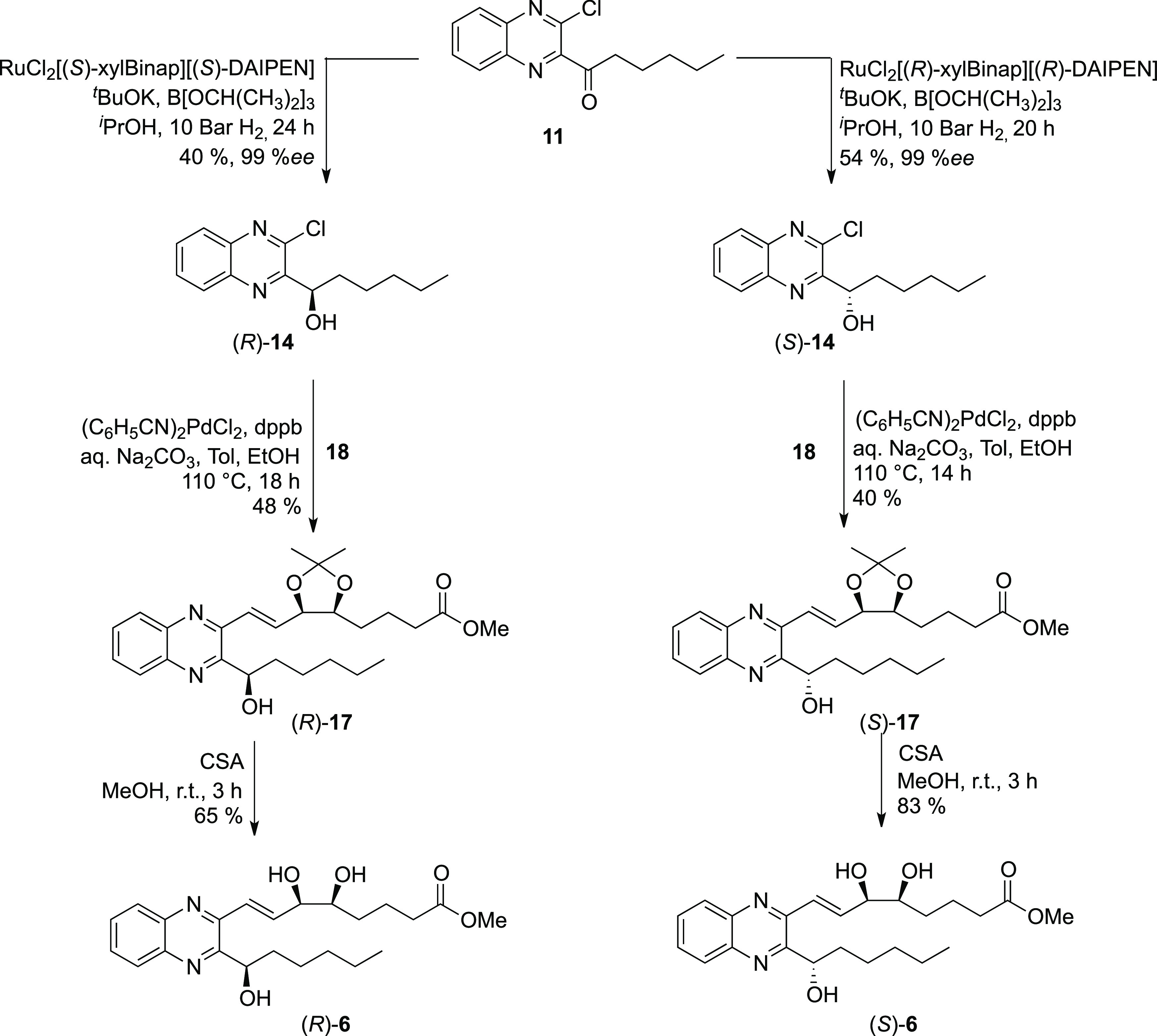
Asymmetric Hydrogenation of Quinoxaline Coupling Partner **11** and Syntheses of (*R*)- and (*S*)-**6**

The asymmetric reduction of the quinoxaline ketone **11** was first attempted using the RuCl[(*R*,*R*)-Tsdpen][*p*-cymene] catalyst in a transfer hydrogenation with formic acid and triethylamine, but low enantioselectivities of 66% were observed. Hydrogenation using Noyori’s catalyst (*R*,*R*)-RuCl_2_[xylBinap]DAIPEN in the presence of potassium *tert*-butoxide and triisopropyl borate proceeded giving excellent enantioselectivity of 99% for both enantiomers, albeit in moderate yields of 40 and 54% for the (*R*)- and (*S*)-enantiomers, respectively; see Scheme 3. The configuration of the enantiomers was confirmed by Mosher’s ester analysis^41^ and matched that predicted by Noyori’s transition states.^42^ The Suzuki coupling reaction between aryl chloride (**14**) and vinyl boronate (**18**) was then attempted using Pd(PPh_3_)_4_, but this failed to furnish any of the desired products. Pd(dppf)Cl_2_ has been reported to be successful in the Suzuki coupling of π-deficient hetero-aryl chlorides,^43^ but our optimized catalyst system used bis(benzonitrile)palladium dichloride together with the ligand 1,4-bis(diphenylphosphino)butane (dppb) giving (1*R*)-**11** in a 48% yield and the (1*S*)-product **11** with a 40% yield (Scheme 3). The acetonide group was removed by reaction with camphorsulfonic acid in methanol, and the final analogues (1*R*)-**6** and (1*S*)-**6** were isolated in 65 and 83% yields, respectively.

**Scheme 4:**
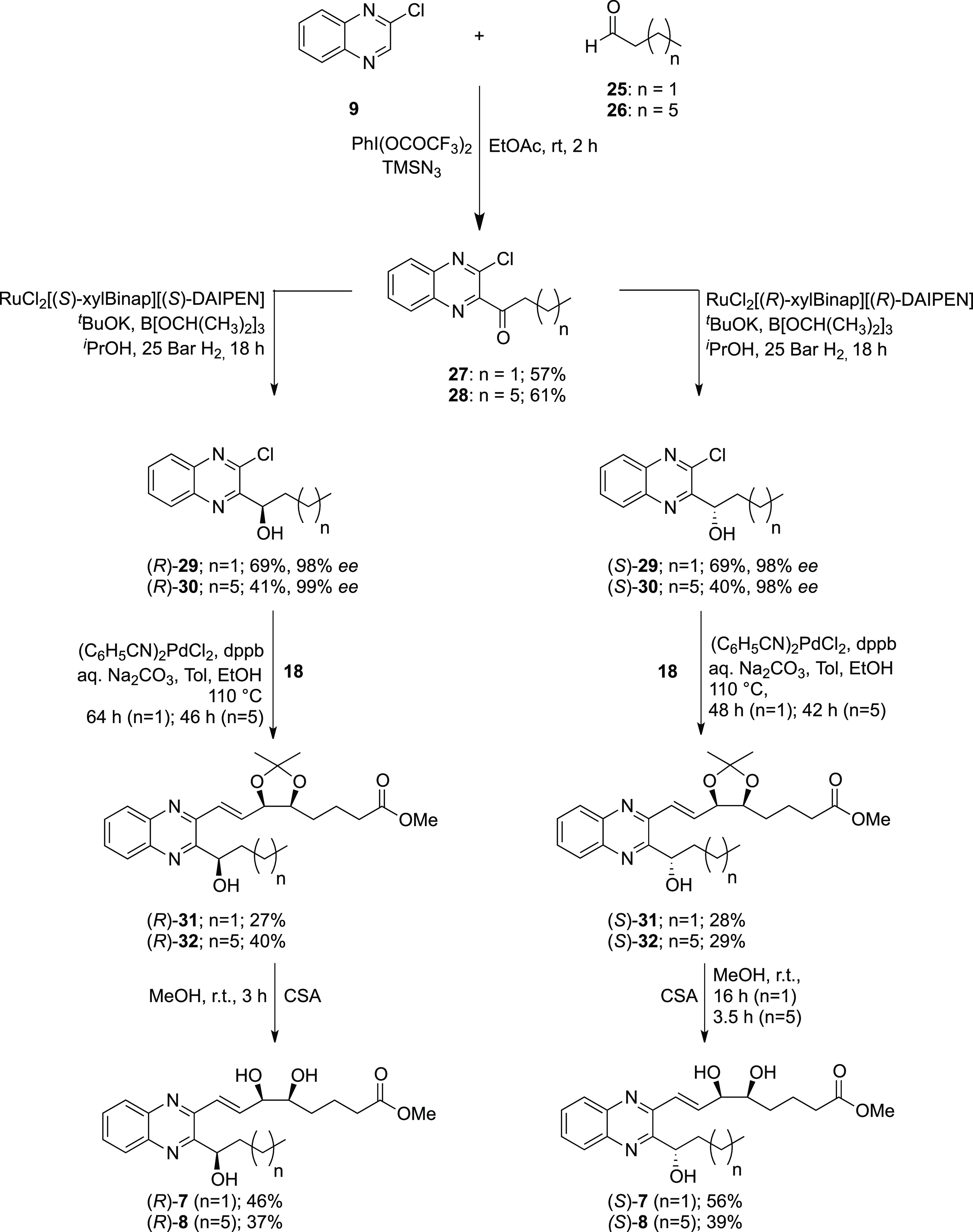
Syntheses of Quinoxaline Mimetics (*R*)- and (*S*)-**7** and (*R*)- and (*S*)-**8** with Varied Alkyl Chain Lengths

Using the same synthetic strategy, four further analogues [(1*R*)-**7**, (1*S*)-**7**, (1*R*)-**8,** and (1*S*)-**8**] with varying lengths of alkyl chain were synthesized to probe the effect this would have on the binding to the receptor and thus biological activity (Scheme 4). This gives a total of six QNX-sLXms, which, in addition to the acetals (*R*)- and (*S*)-**17**, were subjected to biological evaluation as described below.

### Biological Evaluation

#### *In Vitro* Screening of QNX-sLXms Identifies (*R*)-**6** as the Lead Modulator of Inflammation

**Figure 2:**
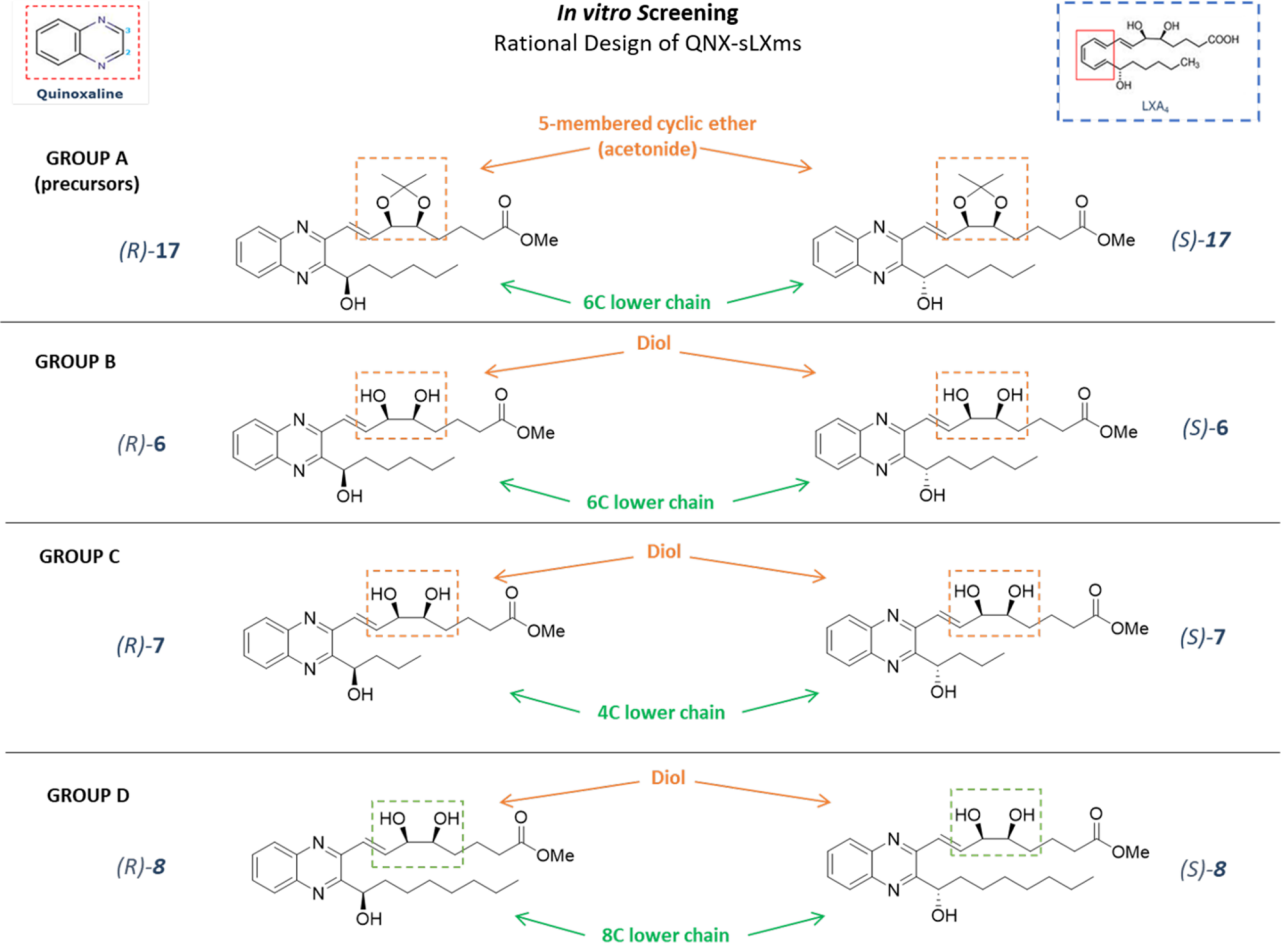
Rational design of QNX derivatives and subgrouping criteria. Strategies adopted to modify the structure of the native LXA_4_ compound (top-right corner) to obtain eight derivatives. QNX-sLXms contain an *ortho*-disubstituted quinoxaline ring (top-left corner). Modifications to the “upper chain” are highlighted in orange, while modifications to the “lower chain” are highlighted in green: based on this classification, the screening has been carried out on four subgroups of candidates [**A** (precursor), **B**, **C**, and **D**].

Using an LPS-challenged human THP-1 monocyte cell line stably expressing an NF-κB luciferase promoter reporter, the anti-inflammatory bioactions of QNX-sLXms were explored, as described previously.^36^ Our work (not shown) and the work of others have shown ALX/FPR2 expression in THP-1 cells.^44^ For screening purposes, the eight candidate compounds were divided into four groups, based on their chemical structure, as described in the “study design” (Figure 2).

For all *in vitro* assays, we derived an aggregate “score” for each compound to describe the pharmacodynamic (PD) profile elicited relative to responses to LXA_4_, thus named the “relative PD score”. This score reflects the maximal % of inhibition of LPS-stimulated response (*I*_max_) [or the maximal % of excitation relative to the vehicle (*E*_max_)], the half-maximal inhibitory or excitatory concentration (IC_50_) or (EC_50_), and the slope of the concentration–response curve [indicated as the Hill–Slope (HS)] (Table S1).

##### QNX-sLXms Attenuate NF-κB Activity in Human Monocytes

**Figure 3:**
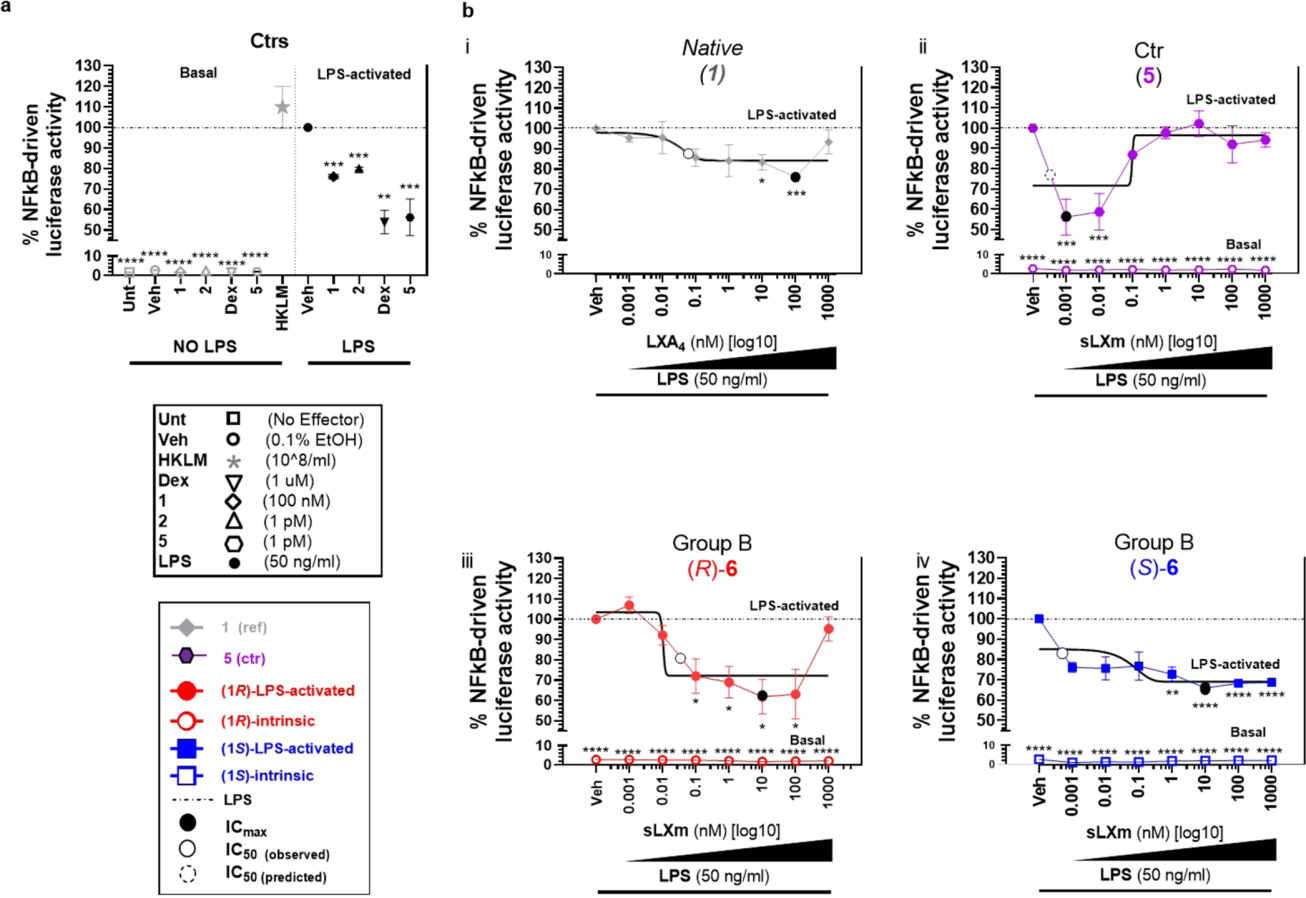
Effect of series (**6**) of QNX-sLXms on LPS-induced NF-κB-driven luciferase activity in monocytes. A total of 1 × 10^5^ THP-1 LUCIA monocytes were pretreated for 30 min with sLXms, vehicle, or appropriate controls, at indicated concentrations in the presence (LPS-activated) or absence (basal) of 50 ng/mL LPS. After 24 h, supernatants were collected and NF-κB-driven luciferase activity was assayed. (a) Single-point analysis of the internal controls. (b) Concentration–response curves of reference compounds (**1**, **5**) and QNX-sLXms. Data are expressed as % ± SEM (*n* = 3) of normalized luminescence unit relative to LPS-induced response. Best-fitting curves are indicated by black solid lines. Statistical analysis was carried out using Student’s unpaired two-tailed *t*-test of the tested compound vs LPS (*p < 0.05; **p < 0.01; ***p < 0.001; ****p < 0.0001) or vs LXA_4_**1** (not shown).

LPS-induced NF-κB-driven luciferase activity was measured in response to all eight candidates (concentration range: 1 pM to 1 μM). LXA_4_ (**1**), previously identified sLXms [specifically, benzo-LXA_4_ (**2**) (1 pM) and imidazolo-LXA_4_ (**5** or **AT-01-KG**)], and the anti-inflammatory cortisol mimetic dexamethasone (Dex; 1 μM) were used as positive controls and/or reference compounds (Figure 3a).

The reference PD profile of LXA_4_ (**1**) showed an *I*_max_ of 24 ± 1% and an IC_50_ of 60 pM. We identified several “hits”: (*R*)-**6** showed the best PD score (*I*_max_ = 38 ± 9%; IC_50_ = 25 pM; *rel. slope = 57*; ***PD score = +5***; *p* < 0.05). Its epimer, (*S*)-**6**, reduced the luciferase activity to a lesser extent (approx. 25–35%) and in a much wider range (1 pM–1 μM) (**PD score = +2**; *p* < 0.0001). In group **C**, both epimers [(*R*)-**7**, (*S*)-**7**] efficiently (by 30–35%) and significantly (*p* < 0.05) decreased the NF-κB activity but were the least potent of all candidates, as demonstrated by IC_50_ in the “nM” range. In group **D**, [(*R*)-**8**, (*S*)-**8**] reduced the activity by about 10–20%, thus showing a lower efficacy compared to the other candidates (IC_50_ in the “pM” range). In contrast, the least effective compounds were the acetonides (*R*)- and (*S*)-**17** (group **A**). In the absence of the LPS challenge, the compounds did not have any effect on the NF-κB activity per se (Figures 3 and S1).

For clarity, data presented in Figure 3 show the effect of (*R*)-**6** and its epimer (*S*)-**6** on LPS-stimulated NF-κB luciferase activity together with relevant controls. Further details of responses to other compounds are provided in Figure S1, as detailed above. Relative PD scores are supplied in Table S2.

##### Quinoxaline-Containing sLXms Hits Differentially Modulate Cytokine Release in Human Monocytes

**Figure 4:**
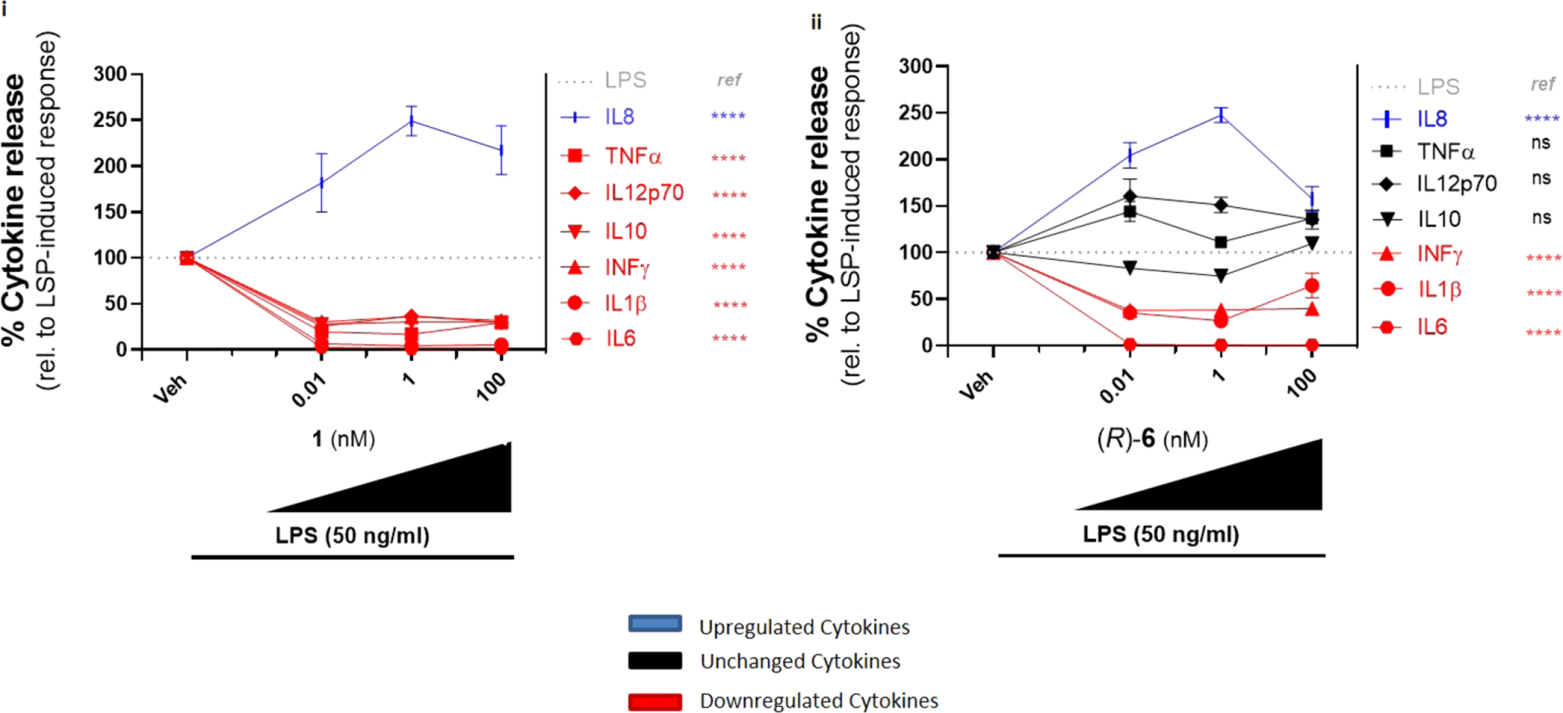
Effect of QNX-sLXm lead compound (*R*)-**6** on the whole panel of proinflammatory cytokine release in monocytes. A total of 1 × 10^5^ THP-1 LUCIA monocytes were pretreated for 30 min at indicated concentrations with LXA_4_**1** (i) or QNX-sLXm (*R*)-**6** (ii). After 24 h from the subsequent stimulation with LPS, supernatants were collected and a panel of seven proinflammatory cytokine levels was measured (IL-6, IL-1β, IFN-γ, IL-12p70, IL-8, IL-10, TNF-α). Concentration–response curves show upregulated (blue), downregulated (red), or unmodified (black) cytokines. Statistical analysis was carried out using Student’s unpaired two-tailed *t*-test of the tested compound vs LPS (ns, not significant; ****p < 0.0001).

To further test the anti-inflammatory bioactions of the six most active compounds (hits) out of the eight candidates, effects on downstream targets of the NF-κB pathway were measured [IL-6, IL-1β, IFN-γ (Figure S2), IL-10, IL-8, IL-12p70, tumor-necrosis-factor-α (TNF-α) (Figure 4)], using multiplex enzyme-linked immunosorbent assay (ELISA) analysis of the supernatant from THP-1 monocytes treated with LPS with or without sLXms.

##### QNX-sLXms from Group **B**, but not **C** and **D**, Abolish IL-6 Secretion

LPS significantly induced IL-6 secretion from monocytes (basal secretion = 1 ± 0.1 pg/mL vs LPS-induced release = 3584 ± 122 pg/mL). As previously reported,^36^ LPS-stimulated IL-6 release was almost completely abolished by LXA_4_ (**1**) (10 pM–100 nM) (*p* < 0.0001), displaying an *I*_max_ of 99 ± 1% and an IC_50_ of 1 pM (Figure S2bi). Similarly, 1 μM Dex abolished IL-6 levels (*I*_max_ = 98 ± 1%, *p* < 0.0001), whereas 1 pM (**2**) attenuated IL-6 concentration to a lesser extent (*I*_max_ = 42 ± 3%, *p* < 0.001) (Figure S2ai). Both mimetics from group **B** significantly (*p* < 0.001) reduced IL-6 release, with the (*S*)-epimer performing better than the native compound, as shown by the positive value of the PD score [relative to LXA_4_ (**1**)]. In particular, (*R*)-**6** displayed *I*_max_ = 84 ± 2%; IC_50_ = 1 pM; *rel. slope = 0.7*; and ***PD score = −2*** (Figure S2ci). The (*S*)-**6** epimer displayed *I*_max_ = 98 ± 1%; IC_50_ = 1 pM; *rel. slope = 2.4*; and ***PD score = +2*** (Figure S2di). On the other hand, mimetics from groups **C** and **D** did not significantly attenuate IL-6 secretion (negative PD scores, ranging from −**4** to −**8**) (Figure S3).

##### QNX-sLXms (*R*)-**6** from Group **B** Inhibits IL-1β Secretion

LPS significantly induced IL-1β secretion (basal secretion = 2 ± 0.5 pg/mL vs LPS-induced release = 1162 ± 53 pg/mL) from monocytes. As we previously demonstrated,^36^ LPS-stimulated IL-1β release was almost completely abolished once again by LXA_4_ (**1**) (10 pM–100 nM), displaying an *I*_max_ of 93–95 ± 1% and an IC_50_ of 1 pM (*p* < 0.001) (Figure S2bii), as well as 1 μM Dex (*I*_max_ = 86 ± 6%, *p* < 0.0001), whereas 1 pM (**2**) maximally reduced secretion by 59 ± 4% (*p* < 0.0001) (Figure S2aii). The (*R*)-epimer from group **B** significantly (*p* < 0.0001) inhibited IL-1β secretion but without outperforming LXA_4_ (**1**) (*I*_max_ = 73 ± 2%; IC_50_ = 1 pM; *rel. slope = −0.03*; ***PD score = −2***) (Figure S2cii); however, its epimer completely lost the protective effect (***PD score = −6***) (Figure S2dii). Mimetics from groups **C** and **D**, although significantly (*p* < 0.05–0.001) attenuating IL-1β secretion, did not outperform the parent compound (negative PD scores of −3 and −4) (Figure S3).

##### All QNX-sLXms Tested Inhibit IFNγ Secretion

LPS significantly induced THP-1 monocyte secretion of IFNγ (basal secretion = 15 ± 2 pg/mL vs LPS-induced release = 146 ± 11 pg/mL). As shown,^36^ LPS-stimulated IFNγ release was attenuated by LXA_4_ (**1**) (10 pM–100 nM), [*I*_max_ = 72 ± 4%; IC_50_ = 1 pM (*p* < 0.001)] (Figure S2biii) as well as 1 μM Dex (by 56 ± 2%, *p* < 0.01), whereas 1 pM (**2**) did not significantly alter LPS-stimulated IFNγ release (Figure S2aiii). The (*R*)-epimer from group **B**, (*R*)-**6**, significantly (*p* < 0.0001) inhibited IFNγ secretion (*I*_max_ = 62 ± 1%; IC_50_ = 1 pM) (Figure S2ciii), while the (*S*)-epimer reduced IFNγ secretion more efficiently but less potently (*I*_max_ = 74 ± 4%; IC_50_ = 2 nM) (Figure S2diii). None of these outperformed LXA_4_ (**1**). Interestingly, both epimers from group **C** significantly (*p* < 0.0001) almost abolished IFNγ secretion, by 85–90 ± 2–4%, IC_50_ = 1–2 pM, with the (*R*)-epimer, (*R*)-**7**, outperforming the native compound (***PD score = + 1***). Both mimetics from group **D**, (*R*)- and (*S*)-**8**, although significantly attenuating (*p* < 0.05–0.0001) IFNγ release, did not perform better than the parent compound (negative PD scores) (Figure S3).

For clarity, the effects of the native (**1**) and the lead compound (*R*)-**6** on all seven cytokines assayed are summarized in Figure 4. The data presented in Figure S2 show the effect of (*R*)-**6** and its epimer (*S*)-**6** only on LPS-stimulated “prototype” inflammatory cytokines (IL-6, IL-1β, INFγ) released together with the relevant controls. Data from the other compounds investigated on these three key cytokines are shown in Figure S3 as detailed above. Relative PD scores are supplied in Table S3.

Notably, LPS-induced secretion of IL-8 was enhanced by 2.5-fold (*p* < 0.001) by Dex (1 μM) (not shown), LXA_4_**1** (1 nM) as well as (*R*)-**6**. Therefore, (**1**) and (*R*)-**6** were equally efficient but more significantly potent than Dex in enhancing IL-8 release (Figure 4).

Overall, (*R*)**-6** was confirmed to be the most effective candidate, downregulating the classical proinflammatory cytokines (IL-6, IL-1β, and IFN-γ) and, interestingly, enhancing secretion of IL-8 from monocytes, similar to controls (**1**) and Dex.

#### *In Vitro* Safety Study of QNX-sLXms Identifies (*R*)-**6** as a Safe “Lead” Modulator of Inflammation

Lactate dehydrogenase (LDH) release is a measure of plasma-membrane integrity conventionally used to assay associated cell damage.^45^ More recently, it has also been recognized as an important inflammatory biomarker, alongside established markers including CRP, IL-1β, and IL-6 in the context of cancer,^46^ pneumonia,^47^ and diabetic retinopathy^48^ models, as well as, very recently, in COVID-19.^49^

**Figure 5:**
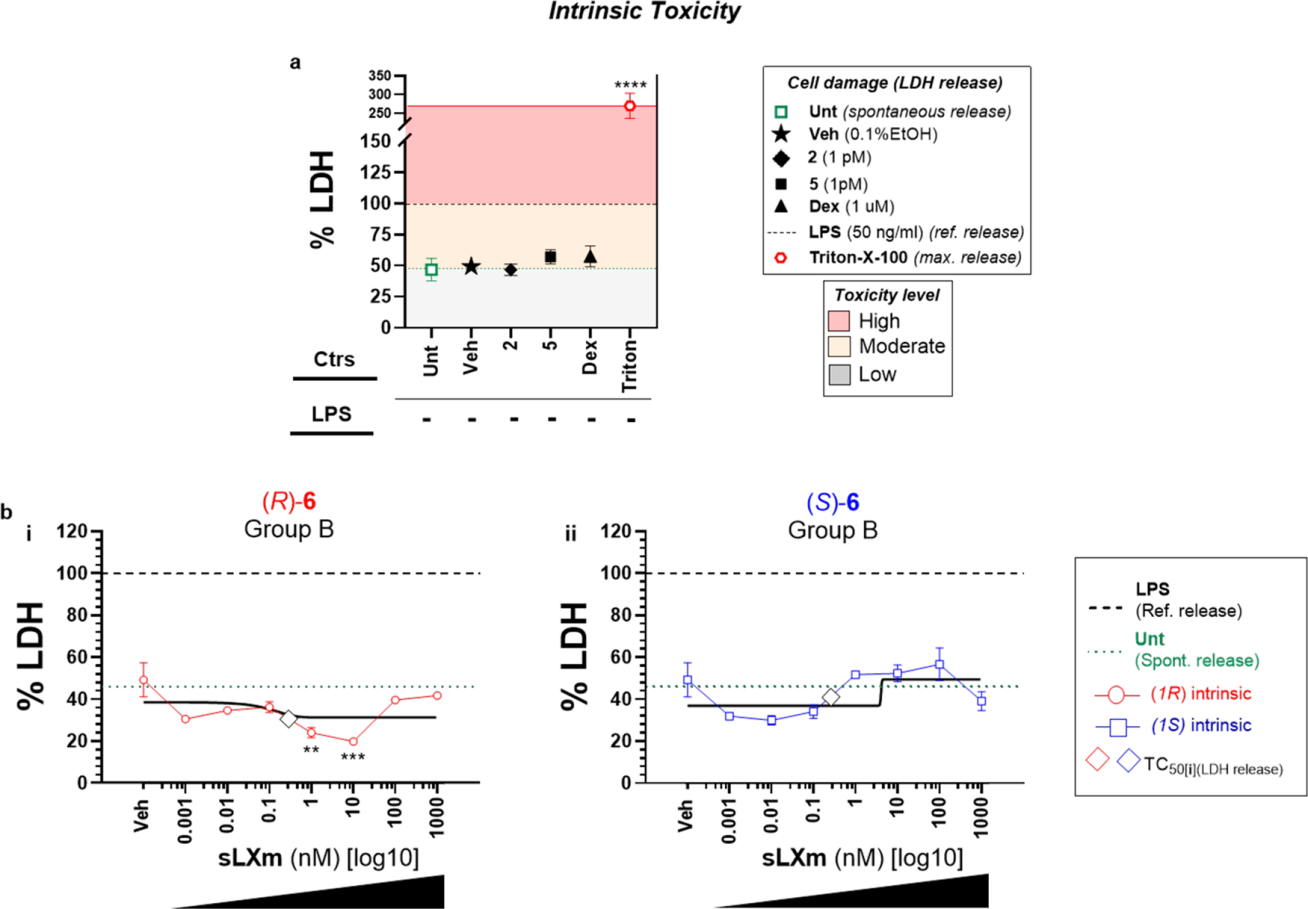
Intrinsic cytotoxic profile of series (**6**) of QNX-sLXms. A total of 1 × 10^5^ THP-1 LUCIA monocytes were treated for 24 h with QNX-sLXms, vehicle, or appropriate controls (1 pM to 1 mM). After 24 h, supernatants were collected and LDH release was assayed. (a) Single-point analysis of controls defines a “high” (red area), “moderate” (yellow area), and “low” (gray area) level of cytotoxicity. (b) Concentration–response and best-fitting curves of (*R*)-**6** and (*S*)-**6**. Data are expressed as % LDH release relative to LPS ± SEM (*n* = 3). Statistical analysis was carried out using Student’s unpaired two-tailed *t*-test of the tested compound vs LPS (**p < 0.01; ***p < 0.001; ****p < 0.0001).

We investigated the potential cytotoxicity of all candidate sLXms by assaying LDH release.^50^ The response to LPS was arbitrarily set to 100%, and values were expressed relative to this. As previously reported by others, LPS provokes LDH release.^51^ The maximal LDH release was observed in response to Triton-X-100 (approximately 3-fold higher than LPS-induced levels). Ultimately, untreated cells defined the spontaneous cell release thresholds. These three responses also defined the thresholds of the LDH-associated level of toxicity, as displayed in Figure 5a.

##### Lead sLXm (*R*)-**6** Displays a Safe Cytotoxic Intrinsic Profile

In the absence of LPS challenge, all tested compounds displayed a safe intrinsic profile, with the exception of acetonides (**17**), which induced an LDH release significantly (*p* < 0.01) higher than the spontaneous cell release. Interestingly, (*R*)-**6**, (*R*)-**7**, and (*S*)-**8** showed LDH levels significantly (*p* < 0.05–0.001) lower than the vehicle, at the nM range. Additionally, Dex (1 μM), **2** (1 pM), or **5** (1 pM) did not display any significant cytotoxic effect, with an LDH release comparable to that of the vehicle (Figures 5 and S4).

##### Lead sLXm (*R*)-**6** Attenuates LPS-Induced Extrinsic Cytotoxicity

**Figure 6:**
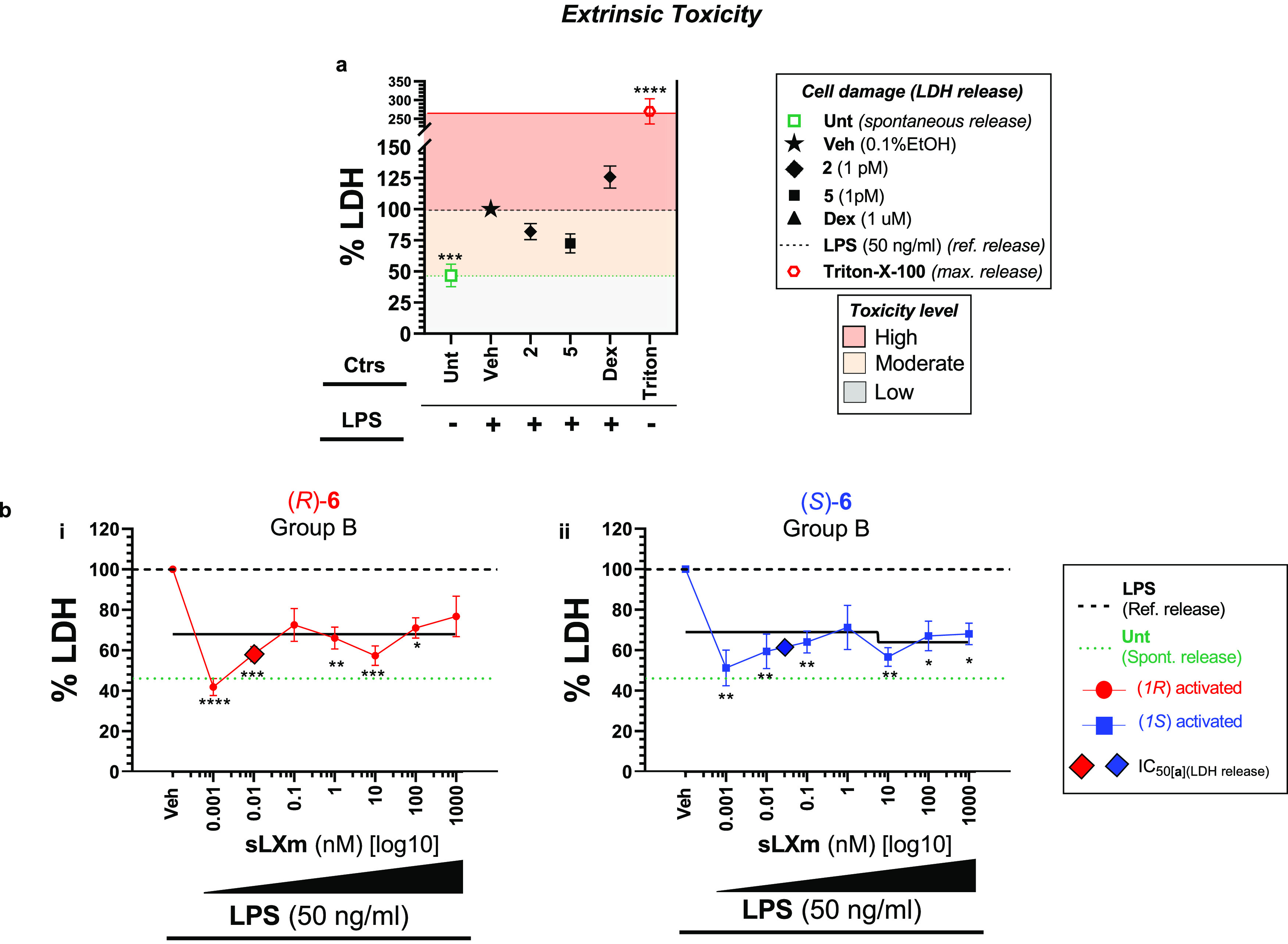
Extrinsic cytotoxic profile of series (**6**) of QNX-sLXms. A total of 1 × 10^5^ THP-1 LUCIA monocytes were pretreated for 30 min with QNX-sLXms, vehicle, or appropriate controls (1 pM–1 μM) and subsequently challenged for 24 h with 50 ng/mL LPS. After 24 h, supernatants were collected and LDH release was assayed. (a) Single-point analysis of controls defines a high (red area), moderate (yellow area), and low (gray area) level of cytotoxicity. (b) Concentration–response and best-fitting curves of (*R*)-**6** and (*S*)-**6**. Data are expressed as % LDH release relative to LPS ± SEM (*n* = 3). Statistical analysis was carried out using Student’s unpaired two-tailed *t*-test of the tested compound vs LPS (*p < 0.05; **p < 0.01; ***p < 0.001; ****p < 0.0001).

In cells stimulated with LPS, a 2-fold increase in LDH release was observed compared to the baseline (from 49 to 100%). In the presence of (*R*)-**6**, cytoprotection was preserved when challenged with LPS for 24 h, significantly (*p* < 0.001) attenuating by 1.3- to 2.4-fold the LPS-induced toxicity (ranging from 42 ± 4% at the lowest dose of (*R*)-**6** up to 77 ± 10%, at the highest). It is noteworthy that Dex (1 μM) did not reduce LPS-induced LDH, as has been reported in similar *in vitro* systems by others,^52^ when compared with *in vivo* observations^53^ (Figures 6 and S5).

##### Lead sLXm (*R*)-**6** Displays a Safe “Activity–Toxicity” Index

A postanalysis “safety study” was conducted on all QNX-sLXms. For each tested compound, we calculated an *in vitro **“Safety index” (S***_***i***_***)*** by relating its “anti-inflammatory half-maximal activity” (the highest dose among the IC_50_ of the extrinsic LPS-challenged phenotype: NF-κB activity, proinflammatory cytokine, and LDH release) to its intrinsic “toxicity” (expressed as the relative half-maximal LDH-associated cytotoxicity). The generated *S*_i_ is potentially predictive of a translational “therapeutic” range: the higher the *S*_i_, the safer the molecule is. As summarized in Table S4, *S*_i_ values from all tested compounds fall in a broad range (7–50). The lead compound (*R*)-**6** displayed an “intermediate” index of **20**, associated with low cytotoxicity.

In summary, the lead compound tested for cytotoxicity displayed safety within the pM to nM range, in the presence or absence of LPS. These data are shown in Figure 5. Data from all compounds tested are shown in Figure S4.

Given the impact on inflammatory responses reported above, subsequent *in vitro* and *in vivo* investigations are detailed for (*R*)**-6**.

#### *In Vitro* Validation of the Anti-Inflammatory Bioactions of Lead Compound (*R*)-**6**

##### (*R*)-**6** Attenuates Inflammatory NF-κB Activity in Smooth Muscle Cells (SMCs)

**Figure 7:**
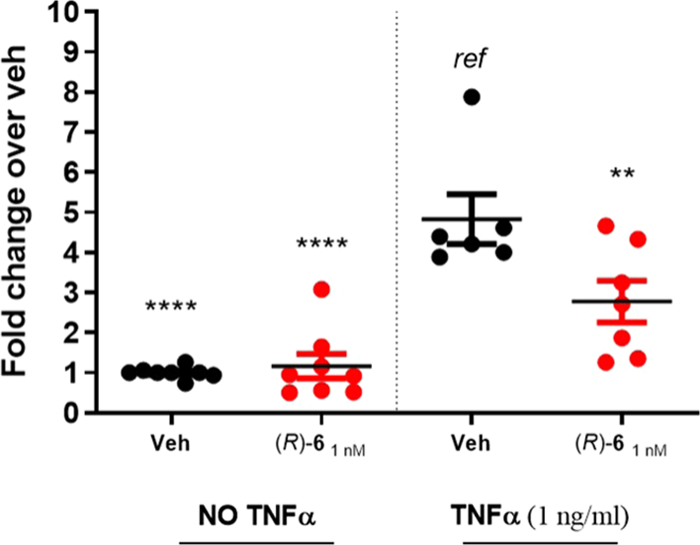
Effect of (*R*)-**6** on TNF-α-induced NF-κB-driven luciferase activity in vSMCs. Mouse primary vSMCs transfected with an NF-κB reporter plasmid (pNF-κB-SEAP vector) were pretreated for 30 min with sLXm (*R*)-**6** (1 nM), vehicle, or appropriate controls. After 24 h from subsequent stimulation with TNF-α (1 ng/mL), supernatants were collected and NF-κB-driven luciferase was assayed. Data are expressed as fold change relative to vehicle ± SEM (*n* = 6–8). Statistical analyses using Student’s unpaired two-tailed *t*-test of the tested compound vs TNF-α (**p < 0.01; ****p < 0.0001).

vSMC-monocyte cross-talk is a key driver of inflammation associated with atherosclerosis.^54^ We evaluated the impact of (*R*)-**6** on TNF-α-stimulated NF-κB in cultured mouse primary vSMCs, as previously described,^18^ by transfecting SMCs with an NF-κB reporter plasmid (pNF-κB-SEAP vector) for 24 h and subsequently stimulating SMCs with TNF-α (1 ng/mL) for 24 h in the presence or absence of a vehicle or (*R*)-**6** (1 nM). We observed a 5-fold increase in NF-κB activity in SMCs in response to TNF-α, which was significantly (*p* < 0.05) reduced to 3-fold by pretreating the TNF-α-challenged cells with the sLXms (*R*)**-6** (Figure 7). As previously reported,^18^ LXA_4_**1** (0.1 nM) and **2** (1 nM) reduced TNF-α-stimulated NF-κB activity in this system.

##### (*R*)-**6** Enhances Macrophage Phagocytosis

We measured the effects of LXs and sLXms on phagocytosis of fluorescent *Escherichia coli* bioparticles by cultured macrophages.^55^ As anticipated from our previous work,^56^ LXA_4_ induced phagocytic activity in macrophages but not in monocytes (data not shown).

**Figure 8:**
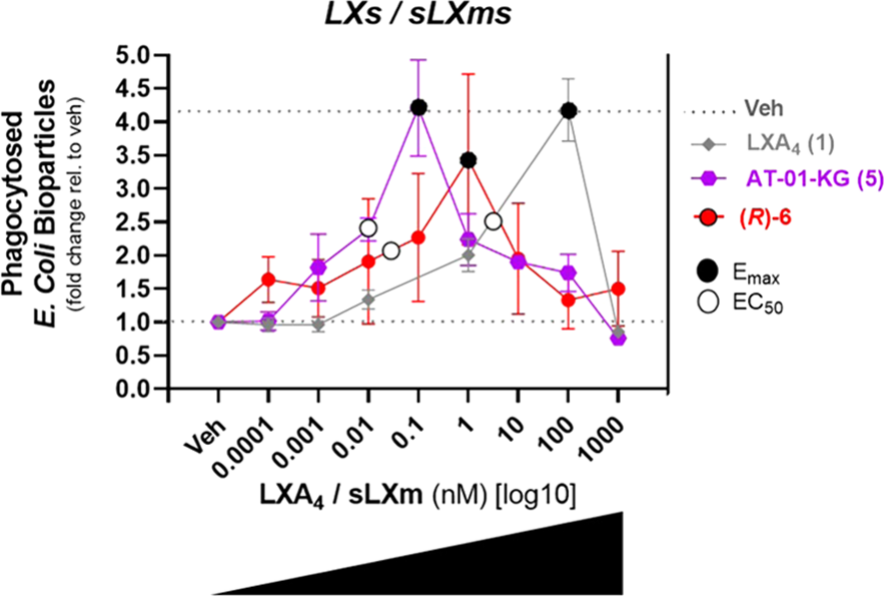
Effects of (*R*)-**6** on *E. coli*-derived bioparticle phagocytosis by THP-1-MF0 macrophage. A total of 1 × 10^5^ THP-1-MF0 macrophage were pretreated for 30 min with sLXms or appropriate controls (1 fM–1 μM), following incubation (for 2 h at 37 °C) with fluorescently labeled *E. coli*-derived bioparticles, prior to measuring the fluorescent signal indicating the uptake of the bioparticles by the macrophages. Data are expressed as fold change ± SEM (*n* = 3). Concentration–response curves show the *E*_max_ (black circles) and EC_50_ (white circles). Statistical analyses were carried out using Student’s unpaired two-tailed *t*-test of the tested compound vs vehicle-treated (veh) (not shown).

Macrophages were pretreated for 30 min with increasing concentrations (1 fM–1 μM) of LXA_4_ (**1**) and sLXms [(**5**) and (*R*)-**6**]. LXA_4_ (**1**) significantly (*p* < 0.01) increased phagocytosis by 4-fold in a concentration-dependent way, reaching a peak at 100 nM and going back to the baseline at 1 μM (*E*_max_ = 4.2 ± 2%; EC_50_ = 5 nM). Overall, by looking at the shape of the curves, the peak of LXA_4_ is right-shifted (low potency), while the peak of sLXms falls right in the middle of the concentration–response curve, resulting in similar efficiency but being more potent (respectively, by 500× and 100×) (positive relative PD scores) than the native compound. This result suggests an anti-inflammatory activity preserving host defense for both sLXms current leads [(**5**) and (*R*)-**6**] by enhancing the phagocytic ability of MF0 macrophages, likely initiating a transition toward an MF2 phenotype (Figure 8).

#### *In Vivo* Validation of the Anti-Inflammatory Bioactions of Lead Compound (*R*)-**6**

With distinct anti-inflammatory responses determined for LXA_4_ and sLXms *in vitro* (cytokine release and NF-κB activation), we next evaluated the lead compound in two *in vivo* models of acute murine inflammation: zymosan-induced peritonitis and carrageenan-induced paw edema.

##### (*R*)-**6** Displays Anti-Inflammatory Activity by Attenuating Murine Zymosan-Induced Peritonitis

**Figure 9:**
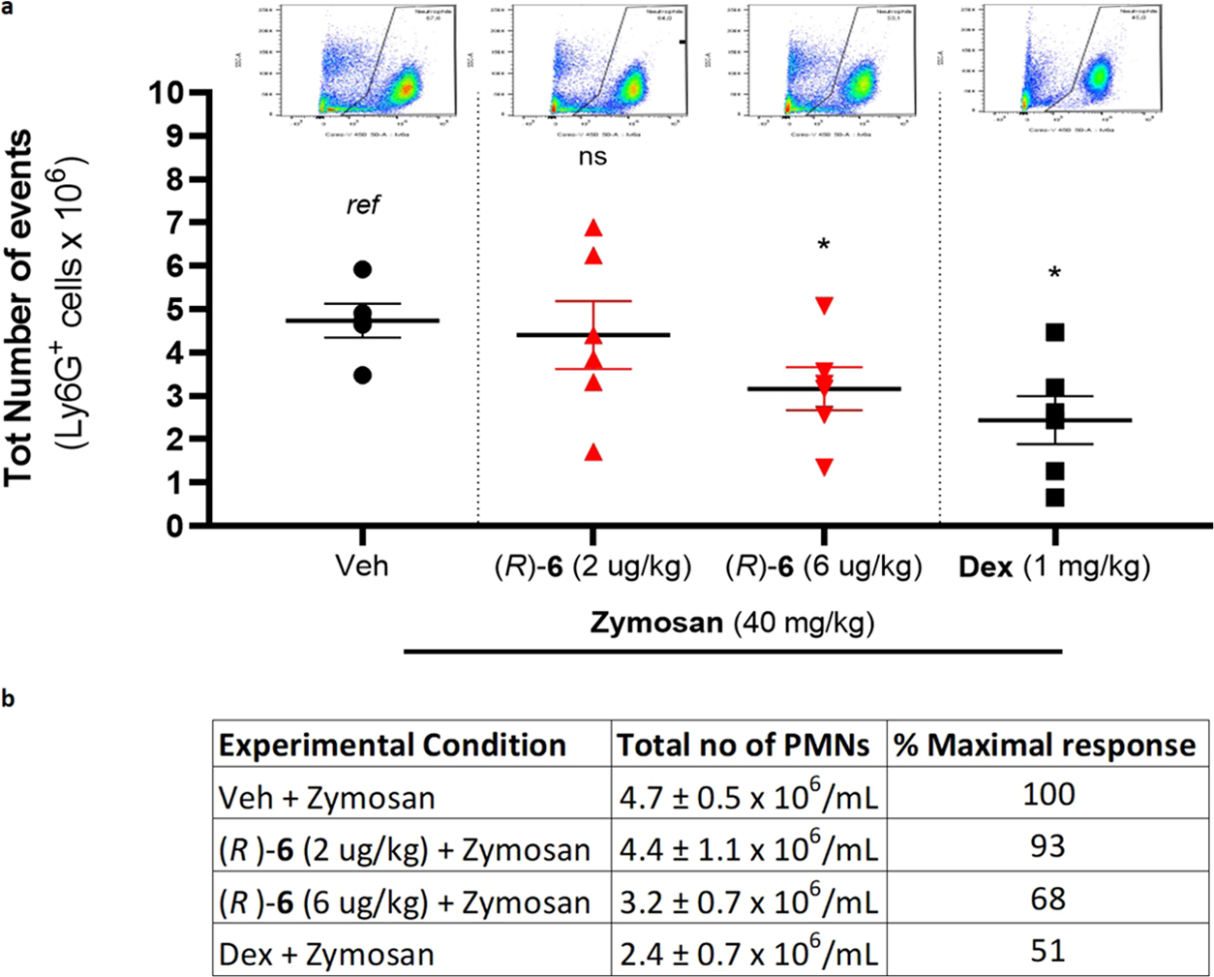
Effect of sLXm (*R*)-**6** on murine zymosan-induced peritonitis. Mice were pretreated with (*R*)-**6** (2 μg/kg and 6 μg/kg) or Dex (1 mg/kg) by ip injection 30 min prior to zymosan (40 mg/kg) administration and again 1 h after zymosan injection. Peritoneal cells were collected by lavage 24 h after zymosan injection. (a) Graphs and dot-plots show PMN counts corresponding to Ly6G^+^ cells. Surface marker expression on peritoneal cells was assessed by flow cytometry. One-way analysis of variance (ANOVA) statistical analysis was performed, *p < 0.05. (b) Table displays the absolute count and relative % of maximal response. Data are presented as mean ± SEM, *n* = 5 mice/treatment group.

Treatment of mice (1 mg/200 μL H_2_O/mouse-zymosan injected ip = 40 mg/kg) was associated with a massive influx of neutrophils into the peritoneal cavity. Pretreatment of the animals with Dex (11 mg/kg, 200 μL ip 1 h prior to zymosan injection) significantly (*p* < 0.01) reduced neutrophil influx by 33.5% of the maximal response. Pretreatment with 6 μg/kg (*R*)**-6** significantly (*p* < 0.05) reduced PMNs by 22.5% of the maximal response (relative to vehicle + zymosan). Mean values of PMN counts were as follows: 4.7 ± 0.5 × 10^6^/mL (vehicle pretreatment and zymosan); 4.4 ± 1.1 × 10^6^/mL (low dose (*R*)-**6** pretreatment and zymosan); 3.2 ± 0.7 × 10^6^/mL (high dose (*R*)-**6** pretreatment and zymosan); and 2.4 ± 0.7 × 10^6^/mL (Dex and zymosan) (Figure 9). It is important to note that sLXms were administered using the same regimen as Dex but with a much lower dosage. Due to the fact that 70% of all leukocytes measured were PMNs, a strongly similar trend was observed when the effect of sLXms on the entire myeloid population (data not shown) or on the PMN fraction was analyzed (Figure 9). Furthermore, cells were specifically stained for PMNs (Ly6G-Pacific Blue) or total macrophages (F4/80-AF488). Total leukocytes were stained with CD11b-APC. Using appropriate isotype controls, quadrants were drawn and data were plotted on logarithmic-scale density- or dot-plots to investigate the relative % of PMNs and monocytes/macrophages by flow cytometry. The relative proportion of each cell type was found not to be altered by treatment with either Dex or sLXms (data not shown).

##### (*R*)-**6** Reduced Acute Inflammatory Response to Carrageenan Paw Swelling

**Figure 10:**
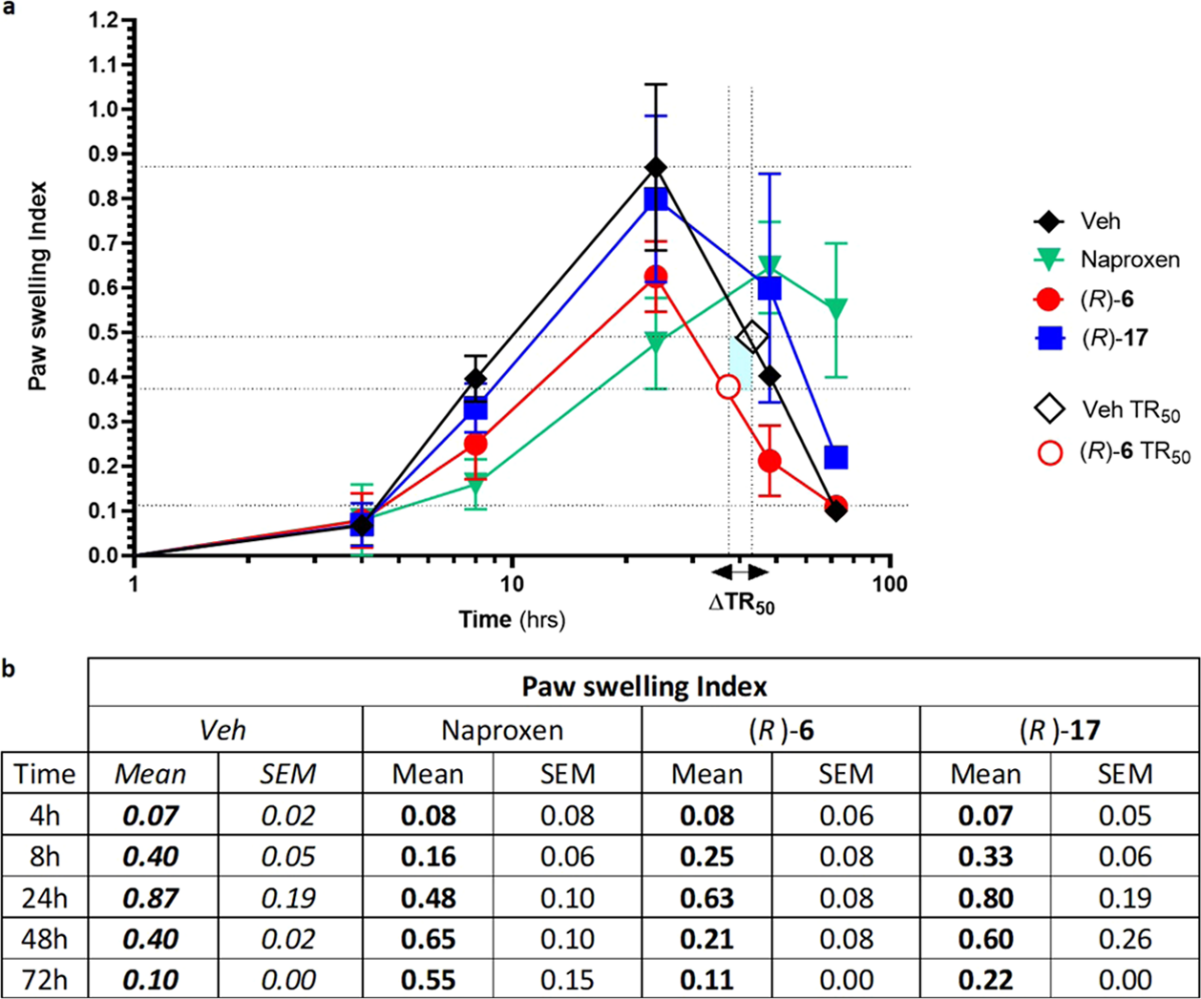
Effect of sLXm (*R*)-**6** on murine carrageenan-induced paw edema. (*R*)-**6**, (*R*)-**17** (2 μg/kg), or Naproxen (50 mg/kg, po) was administered 30 min before the intrapaw injection of 1% carrageenan into male C57bl/6 mice. Paw swelling was monitored over time using an external lever gauge. (a) Graph shows the paw edema index. One-way ANOVA statistical analysis was performed, *p < 0.05, **p < 0.01, ***p < 0.001. (b) Table displays the index of each tested molecule relative to the carrageenan-induced levels. Data are presented as mean ± SEM, *n* = 3 mice/treatment group.

In a murine model, carrageenan injection (1%) induced a self-limiting inflammatory response (paw swelling) that resolved after 72 h. The peak of inflammation was observed at 24 h as previously described.^57^ Inflammation was markedly attenuated in animals treated with (*R*)-**6** (2 μg/kg, ip): the peak response observed was significantly reduced (*p* < 0.05) relative to that seen in vehicle-treated animals (Figure 10). Interestingly, the time taken to reduce the inflammatory response to 50% max is reduced in (*R*)-**6-**treated animals by 6 h relative to that in vehicle-treated animals. This interval is designated in Figure 10 as ΔΤR_50_. Consistently, (*R*)-**17**, a compound without effect on inflammatory responses *in vitro* (Figure S2), had no effect on the inflammatory response in this model. The conventional NSAID Naproxen (50 mg/kg, po) was included in these studies for comparison. The efficacy of (*R*)-**6** (2 μg/kg) was comparable to that of Naproxen regarding reduction of the peak of inflammation (Figure 10). The difference in potency between (*R*)-**6** and Naproxen is noteworthy: 2 μg/kg vs 50 mg/kg, respectively. In Naproxen-treated animals, inflammation persisted after 72 h, the interval at which spontaneous resolution was observed (Figure 10).

#### QNX-sLXm (*R*)-**6** Is an ALX/FPR2 Receptor Agonist

**Figure 11:**
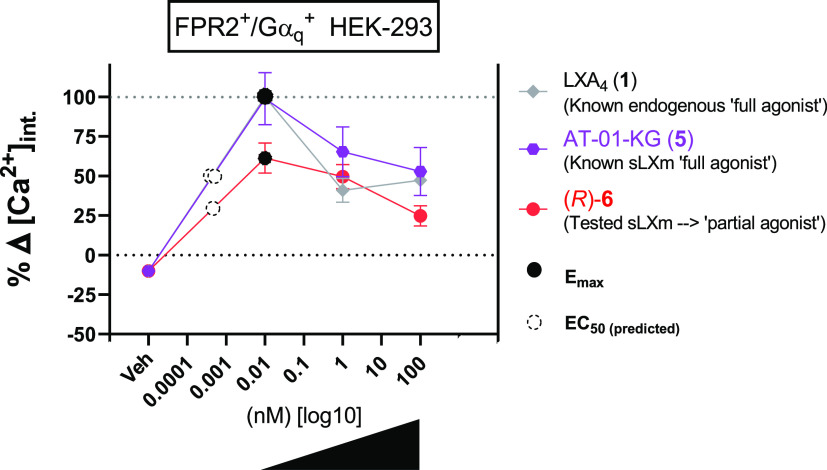
Effects of (*R*)-**6** on intracellular calcium flux in stably transfected HEK-293. Cells were cultured for 18 h prior to labeling with Fluo-4 (37 °C, 1 h). (a) Quantification of three independent experiments was carried out by calculating differential calcium signals measured at the baseline and at maximum peak. Data are expressed as % delta calcium-induced fluorescent signal relative to the peak of a known full agonist (**1**) ± SEM (*n* = 3). Concentration–response curves for LXA_4_ (**1**) and sLXms (10 pM to 100 nM) show the *E*_max_ (black circles) and predicted EC_50_ (white dotted circles). Statistical analyses were carried out using Student’s unpaired *t*-test of the tested compound vs veh (not shown).

ALX/FPR2 is a G-protein-coupled receptor that is activated by the endogenous ligands, including LXA_4_.^58^ The molecular target of sLXms was investigated using a cell line stably expressing the ALX/FPR2 receptor coupled to a Gα_q_ subunit.^59^ ALX/FPR2 activation is coupled to Ca^2+^ release in this experimental system. Using HEK-293 cells stably expressing the ALX/FPR2 receptor together with a Gα_q_ subunit,^60^ receptor activation was determined by transient Ca^2+^ flux. The control used was wild-type HEK cells to verify the specificity of the agonism toward the ALX/FPR2 receptor, as previously described^36^ (Figure S6). Treatment of cells with 100 nM LXA_4_**1** or (*R*)-**6** (10 pM–100 nM) resulted in increased intracellular Ca^2+^, although, through a direct comparison of LXA_4_**1** and (*R*)-**6** at the same concentration (10 pM), where they both maximally activate ALX/FPR2, it is evident that activation by the mimetic was significantly (*p* < 0.05) 40% lower than that induced by the native LXA_4_ (a full agonist) (Figure 11). These data suggest that the mimetic acts as a partial agonist of ALX/FPR2. The partial agonism is also evidenced by the negative PD score relative to the native compound **1** (Table S7). Moreover, in line with observations from LXA_4_ (**1**) and the imidazolo-mimetic (**5**), at 10 pM and 1 nM, (*R*)-**6** induced a similar activation, which was significantly (*p* < 0.01) stronger than that observed at 100 nM. These findings suggest that at higher doses (>100 nM) the interaction with the receptor may reach saturation, probably due to desensitization and/or internalization. These experiments used ATP and W-peptide (Wp) as positive controls. ATP-induced activation of Ca^2+^ mobilization is independent of ALX/FPR2 (mediated via the GPCR purinergic receptor P2Y endogenously expressed on HEK-293 cells).^61^ Ca^2+^ mobilization was observed in ALX/FPR2 expressing cells as a result of stimulation with ATP (1 μM) or Wp (2 nM). Importantly, ATP stimulated Ca^2+^ mobilization in wild-type HEK-293 cells, whereas the ALX/FPR2 ligands (sLXms, Wp, LXA_4_) were without effect (Figure S6). In summary, our data show that (*R*)-**6** is a “partial agonist” at ALX/FPR2.

## Discussion

The well-established metabolic inactivation of LXA_4_**1**,^31^ along with the expense and complexity of its synthesis, significantly limits its potential as a therapeutic agent despite reports of its benefits for the treatment of both acute^2^ and chronic inflammation.^4^ Using the native LXA_4_ structure for inspiration, we previously prepared and evaluated the anti-inflammatory activities of a number of benzo-,^34^ pyridino-,^35^ and imidazolo-mimetic^36^ LXA_4_ analogues. Now, we describe the asymmetric synthesis of a focused library of quinoxalino-QNX-sLXms (**6**–**8**). The successful synthetic route employed set up the required stereochemistry through a combination of chiral pool (2-deoxy-d-ribose) and asymmetric synthesis (ketone hydrogenation). The Suzuki coupling reaction was used for the formation of the heterocycle to the alkene bond and offers an alternative approach to the one using the Heck reaction to prepare other heteroaromatic-containing lipoxins.

The above-detailed synthetic strategy successfully led to eight novel QNX-sLXms, which were screened *in vitro* for their biological activity and safety profile. The anti-inflammatory activity was evaluated by measuring their ability to regulate LPS-induced inflammation in THP-1 Lucia monocytes and subsequent cytokine release. Native LXA_4_ (**1**), a previously reported LXA_4_ analogue [benzo-LXA_4_ (**2**)],^34^ the current sLXm lead compound (imidazolo-LXA_4_**5**),^36^ and dexamethasone^62^ were used as controls.

Briefly, the *in vitro* screening of the novel analogues demonstrated that all tested sLXms, except for the QNX-precursor acetonides (**17**), displayed a similar or more active profile than native LXA_4_ (**1**) (*I*_max_ = 24 ± 1%; IC_50_ = 60 pM; *r*el.* slope = 1*; ***PD score = ref***) in attenuating LPS-induced NF-κB activity. Therefore, the acetonides (**17**) were excluded from further characterization. Among the six remaining QNX-sLXms tested, the quinoxaline analogue containing an alkyl chain of the same length as **1** (6C chain) was shown to be approximately twice as effective as **1**, thus conferring (*R*)-**6** with a general PD score of “**+5**” relative to the native compound. The SAR analysis demonstrated that increasing the length of the alkyl chain by 2 carbons (thus obtaining an 8C chain) did not improve efficacy, but slightly increased potency, compared to **1** (*I*_max_ = 23 ± 8%; IC_50_ = 10–40 pM; rel. slope = 0.5). Reducing the length of the alkyl chain by two carbons (thus obtaining a 4C chain) not only reduced the potency but also ameliorated the efficacy (*I*_max_ = 29–36 ± 8%; IC_50_ = 1–4 nM; rel. slope = 0.6) (Figure 3, Figure S2, and Table S2).

Furthermore, several of the analogues attenuated proinflammatory cytokine release. In particular, (*R*)-**6** demonstrated an immunomodulatory phenotype very similar to LXA_4_ (**1**) by dramatically reducing the levels of IL-1b, IL-6, and IFN-γ (Figure 3) and by enhancing IL-8 secretion, as clearly depicted in Figure S3.

Notably, (**1**) and (*R*)-**6** were equally efficient but more potent than Dex in enhancing LPS-induced release of IL-8 by 2.5-fold (Figure S3). IL-8 has long been recognized to have both pro- and anti-inflammatory activities, which has been established in various models of infection, inflammation, and cancer.^63^ IL-8 is known to inhibit leukocyte adhesion to activated endothelial cells and thus exhibits anti-inflammatory properties. Interestingly, IL-8 possessing 72 amino acids is ca. 10-fold more potent in inhibiting neutrophil adhesion than the corresponding IL-8 variant containing 77 amino acids.^64^ It has been shown that tissue-specific variations in endothelial chemokine secretion rather than variations in adhesion molecules can explain the different patterns of inflammation and leukocyte traffic seen in nonlymphoid tissues.^64^ These data lead us to propose that, in a similar manner to what happens in the endothelium, different monocyte subtypes release different IL-8 isoforms. In this way, LXs and sLXms may differentially induce a monocyte switch, triggering the release of the more potent isoform that inhibits the adhesion of neutrophils: the IL-8_72_. Further studies will address this hypothesis.

Overall, by comparing the effect of the three main compounds tested [native compound, LXA_4_ (**1**), and the two sLXm leads, (*R*)-**6** and (**5**)] on cytokine release, it is evident that all reduce NF-κB activity (Figures 3 and S2). However, their downstream effect is a “fine tuning” of cytokine release through a series of intermediate states, ranging between the abolishment of IL-6 and the enhancement of IL-8 (Figure S3). Such diverse actions can be partially explained by the fact that certain cytokines are simultaneously regulated by multiple transcription factors. Therefore, the inhibitory effect exerted on NF-κB is not sufficient to explain the pleiotropic responses observed, and it is reasonable to suggest epigenetic regulation of cytokine expression in response to SPMs.^65^

Evaluation of putative safety profiles by relating the biological half-maximal activity to the intrinsic and extrinsic toxicities of the compounds supported the conclusion that *R*-(**6**) displayed a suitable safety profile for selection as the “lead compound”, thus warranting validation *in vitro* and *in vivo*.

Given the importance of the cross-talk between monocytes and vSMCs,^54^ the anti-inflammatory ability of *R*-(**6**) was also confirmed in a different *in vitro* model: vSMCs stimulated with TNF-α, thus mimicking a sterile inflammatory scenario in contrast to LPS-evoked responses. The outcome confirmed the ability of *R*-(**6**) to inhibit the NF-κB activity (Figure 7).

Having demonstrated the significant anti-inflammatory properties of *R*-(**6**), it was relevant to test its effect on the phagocytic ability of unprimed macrophages (MF0) derived from THP-1 monocytes. It was revealed that sLXms-lead compounds, **5** and (*R*)-**6**, displayed a similar efficacy to LXA_4_ in enhancing *E. coli* bioparticles’ phagocytosis by MF0 macrophages (Figure 8), thus suggesting an anti-inflammatory action, which preserves innate host defense against microbial invasion.

*In vivo* validation of the therapeutic potential of *R*-(**6**) was investigated in two murine acute inflammatory models: zymosan-induced peritonitis and carrageenan-induced paw edema. The former showed a reduced neutrophil count in the peritoneal lavage in response to (*R*)-**6** administration, suggesting a reduced infiltration of those immune cells in the peritoneum (Figure 9). The latter model demonstrated an (*R*)-**6**-mediated reduction in paw edema.

By comparing the kinetics of the lead compound with the known endogenous and exogenous full agonists of ALX/FPR2 (*E*_max_ set to 100%), (*R*)-**6** could be identified as equipotent partial agonist at ALX/FPR2, having an *E*_max_ less than 100% but displaying the same potency as the full agonists. This suggested that QNX-sLXms, particularly (*R*)-**6**, induced a mild effect at lower doses, which increased with higher concentrations, until it reached a peak (*I*_max_), after which the effect is reduced. These findings suggest desensitization or internalization of the receptor. This is in keeping with ALX/FPR2 internalization that we previously reported in response to LXA_4_ at 1 nM.^14^ This is also supported by our calcium mobilization analysis, where the calcium peak is reached at the “pM range” for both LXA_4_ and sLXms, which is consistent with the hypothesis of a desensitization/internalization of ALX/FPR2 occurring at the “nM range” (Figure 11). Since a molecule can activate more than one receptor (as is the case of epi-LXA_4_),^66^ the specificity of the interaction of (*R*)-**6** with ALX/FPR2 was assessed in the wild-type HEK-293 (which constitutively expresses low levels of the receptor). In such a system, no calcium release was induced, thus confirming the selectivity of the agonism of sLXm for ALX/FPR2 (Figure S6). Therefore, (*R*)-**6** was chosen as the lead compound from this series and evaluated in further biological assays.

Drug discovery routinely tests compounds for their activity against a particular receptor using isolated tissues and high-throughput assays. For compounds that behave as agonists, EC_50_ and *E*_max_ are the parameters normally measured, whereas observed affinity and intrinsic efficacy are parameters of more importance to drug development.

An important aspect of this study was the development of a novel method for the analysis of an agonist’s concentration–response curve so that the product of observed potency, efficacy, and safety expressed relative to a standard agonist can be estimated. This most comprehensive parameter, which we term the **“*****relative pharmacodynamic [PD] score*****”**, implements and complements pre-existing indices [i.e., the intrinsic relative activity (RAi) by Griffin].^67^ It is readily applicable (1) for analyzing responses at G-protein-coupled receptors; (2) for detecting agonist-directed signaling: [upstream of the second messenger level (i.e., calcium mobilization), middle stream (i.e., NF-κB activity), and downstream response (i.e., cytokine release)]; and (3) for a more accurate *in vitro* screening tool, being based on a more comprehensive index than pre-existing ones. In fact, the PD score is equivalent to a “summative index” of the potency, efficacy, and slope of curve ratios that agonists would be predicted to exhibit in an assay that is hypothetical and highly sensitive in which all agonists act as full agonists, even those that possess low levels of intrinsic efficacy.

Taken together, this novel pharmacodynamic approach supports and confirms the novel inflammatory regulator potential of heteroaromatic sLXms, as already demonstrated by us for LXs^56a^ and imidazole-sLXms.^36^

## Conclusions

In this study, eight novel sLXm analogues were designed and successfully prepared in an asymmetric synthesis mainly consisting of a combination of chiral pool (2-deoxy-d-ribose) and enantioselective ketone reduction. These eight candidate molecules were biologically evaluated using an innovative scoring system (PD score) based on three PD components (potency, efficacy, slope). The PD study was conducted (1) to analyze the upstream response at the GPCR ALX/FPR2 in HEK-293 cells (by measuring the stimulation of calcium mobilization through Ga_q_); and (2) to screen the candidate molecules through a novel *in vitro* approach (by measuring the NF-κB activity and the downstream release of proinflammatory cytokines and LDH of monocytes as well as the phagocytic activity of macrophages).

In summary, all tested QNX-sLXms were shown to have minimal toxic effects on human monocytes and displayed a similar or more active profile than the native LXA_4_ (**1**) in attenuating LPS-induced NF-κB activity. Of the QNX-sLXms tested, the quinoxaline analogue (*R*)-**6** demonstrated a superior PD profile than the native compound. The effect of (*R*)-**6** was especially noteworthy in the context of attenuation of cytokine release. The SAR analysis demonstrated that increasing the alkyl chain length led to a reduction in efficacy, while reducing the alkyl chain length also negatively affected potency. Combining the outcomes from subsequent *in vivo* validation models, a role emerged for (*R*)-**6** as an “immuno-modulator” of the neutrophil count and edema formation.

These data clearly demonstrate the therapeutic potential of QNX-sLXms as novel inflammatory regulators.

## Experimental Section

### General Information

#### Chemistry Materials and Methods

##### General Experimental

Starting materials were supplied from commercial sources and used without further purification. All commercially available solvents were used as supplied unless otherwise stated. Anhydrous diethyl ether (Et_2_O), tetrahydrofuran (THF), and dichloromethane (CH_2_Cl_2_) were obtained from a Grubbs-type still, supplied by the Innovative Technology Inc. Pure Solv-400-3-MD solvent purification system. Oxygen-free nitrogen was obtained from BOC gases and was used without further drying. Infrared spectroscopy was performed on a Varian FT-IR 3100 spectrometer. ^1^H NMR spectra were recorded on Varian-Inova spectrometers (300, 400, and 500 MHz) using tetramethylsilane as an internal standard. ^13^C NMR spectra were recorded on 400 and 500 MHz Varian-Inova spectrometers (101 and 125 MHz) using tetramethylsilane as an internal standard. ^19^F NMR spectra were recorded on a 400 MHz Varian-Inova spectrometer (376 MHz). CDCl_3_, purchased from Aldrich, was used as supplied. High-resolution mass spectra (HRMS) were obtained using a Micromass/Walters LCT instrument. Supercritical fluid chromatography (SFC) was performed on a Waters Acquity UPC^2^ instrument with Chiralpak IA3, IB3, IC3, and ID3 columns. High-performance liquid chromatography (HPLC)-grade solvents, purchased from Aldrich, were used as supplied. TLC was performed on Merck precoated Kieselgel 60 F_254_ aluminum plates with realization by UV irradiation or by charring with acidic vanillin. Flash column chromatography was performed on Davisil silica LC60A, particle size 0.040–0.063 mm. Optical rotation measurements were recorded using a Schmidt-Haensch Unipol L2000 polarimeter at 589 nm and are quoted in units of deg dm^–1^ cm^3^ g^–1^ (concentration *c* is given in g/100 mL). Melting points were determined in open capillary tubes with a Barnstead Electrothermal 9300 melting point apparatus. All tested compounds have a purity > 95% as determined by HPLC.

#### Experimental Method

##### 1-(3-Chloro-quinoxalin-2-yl)-hexan-1-one (**11**)

2-Chloroquinoxaline (500 mg, 3.04 mmol, 1 equiv) was dissolved in EtOAc (23 mL). Hexanal (1.5 mL, 12.15 mmol, 4 equiv) and TMSN_3_ (0.8 mL, 6.08 mmol, 2 equiv) were added. PhI(OCOCF_3_)_2_ (2.61 g, 6.08 mmol, 2 equiv) was added portionwise over 10 min, and the mixture turned orange in color. The mixture was stirred at room temperature for 2 h. Additional TMSN_3_ (0.8 mL, 6.08 mmol, 2 equiv) and PhI(OCOCF_3_)_2_ (2.61 g, 6.08 mmol, 2 equiv) were added, and the reaction mixture was stirred for a further 16 h. Triethylamine (7 mL) was added dropwise, and the mixture was stirred for 15 min, filtered through Celite, and washed with 10% CuSO_4_ solution (3 × 50 mL), water (25 mL), and brine (25 mL). The organic layer was dried with MgSO_4_, filtered, and concentrated. The crude mixture was purified by silica gel column chromatography (50:1 → 20:1 pentane/EtOAc) to afford product **11** as a yellow solid (572 mg, 72%). *R_f_* = 0.4 (20:1 pentane/EtOAc); mp = 45–48 °C; ^1^H NMR (400 MHz, CDCl_3_) δ 8.12 (dd, *J* = 8.0, 1.5 Hz, 1H), 8.04 (dd, *J* = 8.0, 1.5 Hz, 1H), 7.85 (m, 2H), 3.18 (t, *J* = 7.5 Hz, 2H), 1.82–1.72 (m, 2H), 1.44–1.31 (m, 4H), 0.91 (t, *J* = 7.0 Hz, 3H); ^13^C NMR (101 MHz, CDCl_3_) δ 200.7, 148.2, 143.7, 142.3, 139.5, 132.6, 130.8, 129.54, 128.3, 40.4, 31.3, 23.2, 22.4, 13.9; IR (neat) (ν_max_, cm^–1^) 3435, 1710, 1265; HRMS (ESI) [M + H]^+^ calcd 263.0951 for C_14_H_16_O^35^Cl; found 263.0941.

##### 1-(3-Chloroquinoxalin-2-yl)hexan-1-ol (**14**)

Ketone **11** (97 mg, 0.369 mmol, 1 equiv) was dissolved in dry MeOH (2 mL) and cooled to 0 °C. NaBH_4_ (40 mg, 1.057 mmol, 2.9 equiv) was added. The reaction was stirred at room temperature for 2 h and then was quenched by the addition of acetone (5 mL). The solvent was removed *in vacuo*, and the mixture was resuspended in a saturated aq NH_4_Cl solution (25 mL) and extracted with CH_2_Cl_2_ (3 × 20 mL). The organic layer was washed with water (20 mL) and brine (20 mL), dried over MgSO_4_, filtered, and concentrated. The crude product was purified by silica gel column chromatography (10:1 pentane/EtOAc) to afford product **14** as a pale-yellow solid (57 mg, 58%). *R_f_* = 0.50 (pentane/EtOAc 6:1); mp = 73–79 °C; ^1^H NMR (400 MHz, CDCl_3_) δ 8.12–8.06 (m, 1H), 8.06–8.00 (m, 1H), 7.88–7.72 (m, 2H), 5.17 (m, 1H), 4.05 (d, *J* = 8.0 Hz, 1H), 2.06–1.98 (m, 1H), 1.65–1.54 (m, 3H), 1.41–1.25 (m, 4H), 0.88 (t, *J* = 7.0 Hz, 3H); ^13^C NMR (101 MHz, CDCl_3_) δ 156.0, 145.4, 141.8, 139.9, 130.9, 130.7, 128.6, 128.4, 70.6, 37.2, 31.7, 25.4, 22.7, 14.2; IR (CHCl_3_) (ν_max_, cm^–1^) 3476, 2958, 1466, 1048; HRMS (ESI) [M + H]^+^ calcd 265.1108 for C_14_H_18_^35^ClN_2_O; found 265.1106.

##### (*S*)-1-(3-Chloroquinoxalin-2-yl)hexan-1-ol ((1*S*)-**14**)

Quinoxaline ketone **11** (50 mg, 0.190 mmol, 1 equiv) was dissolved in *^i^*PrOH (2.5 mL). RuCl_2_[(*R*)-xylBinap][(*R*)-DAIPEN] (12 mg, 0.0095 mmol, 0.05 mmol), ^*t*^BuOK (6 mg, 0.0535 mmol, 0.28 equiv), and tri*iso*propyl borate (0.01 mL) were added, and the reaction mixture was stirred under 10 bar of H_2_ for 24 h. The mixture was concentrated and purified by silica gel column chromatography (10:1 pentane/EtOAc), and product (1*S*)-**14** was isolated as a pale-yellow solid (27 mg, 54%). Mp = 78–81 °C; [α]_D_ = −7.22 (*c* = 1.5 in CHCl_3_); *ee* = 97% as determined by SFC using a Chiralpak IC column (CO_2_/MeCN, gradient 99:1 0–1 min and then gradient to 60:40 until 5 min, 3 mL/min), *R*_t_ = 3.00 min (*S*)-enantiomer, 3.41 min (*R*)-enantiomer. Identical in all other physical data to the previously prepared racemic **14**.

##### (*R*)-1-(3-Chloroquinoxalin-2-yl)hexan-1-ol ((1*R*)-**14**)

Quinoxaline ketone **11** (100 mg, 0.381 mmol, 1 equiv) was dissolved in *^i^*PrOH (3 mL). RuCl_2_[(*S*)-xylbinap][(*S*)-DAIPEN] (23 mg, 0.019 mmol, 0.05 mmol), ^*t*^BuOK (11 mg, 0.095 mmol, 0.25 equiv), and tri*iso*propyl borate (0.02 mL) were added, and the reaction mixture was stirred under 10 bar of H_2_ for 18 h. The mixture was concentrated and purified by silica gel column chromatography (10:1 pentane/EtOAc), and product (1*R*)-**14** was isolated as a pale-yellow solid (40 mg, 40%). Mp = 78–81 °C; [α]_D_ = +13.49 (*c* = 1.5 in CHCl_3_); *ee* = 98% as determined by SFC using a Chiralpak IC column (CO_2_/MeCN, gradient 99:1 0–1 min and then gradient to 60:40 until 5 min, 3 mL/min), *R*_t_ = 3.00 min (*S*)-enantiomer, 3.41 min (*R*)-enantiomer. Identical in all other physical data to the previously prepared racemic **14**.

##### 1-(3-Vinylquinoxalin-2-yl)hexan-1-ol (**15**)

Pd(PPh_3_)_4_ (31 mg, 5 mol %) and LiCl (80 mg, 1.887 mmol, 4 equiv) were dissolved in dry 1,4-dioxane (2 mL). Aryl chloride **14** (121 mg, 0.457 mmol, 1 equiv), dissolved in dioxane (3 mL), was added, followed by tributyl(vinyl)stannane (0.17 mL, 0.589 mmol, 1.3 equiv), and the reaction mixture was heated to 80 °C, sealed under nitrogen, and stirred for 16 h. The reaction mixture was allowed to cool, filtered through a pad of celite, eluted with CH_2_Cl_2_ (20 mL), and concentrated. The crude product was purified by silica gel column chromatography (10:1 cyclohexane/EtOAc) to afford product **15** as an orange oil (104 mg, 88%). *R_f_* = 0.6 (6:1 pentane/EtOAc); ^1^H NMR (400 MHz, CDCl_3_) δ 8.11–8.04 (m, 1H), 8.04–7.99 (m, 1H), 7.76–7.67 (m, 2H), 7.04 (dd, *J* = 16.8, 10.7 Hz, 1H), 6.67 (dd, *J* = 16.8, 1.7 Hz, 1H), 5.75 (dd, *J* = 10.7, 1.7 Hz, 1H), 5.14 (td, *J* = 7.5, 3.0 Hz, 1H), 4.65 (d, *J* = 7.5 Hz, 1H), 1.87 (dt, *J* = 9.8, 7.0 Hz, 1H), 1.59–1.48 (m, 3H), 1.35–1.26 (m, 4H), 0.90 (t, *J* = 7.5 Hz, 3H); ^13^C NMR (101 MHz, CDCl_3_) δ 155.1, 147.6, 141.8, 140.0, 130.5, 129.8, 129.7, 129.2, 128.2, 123.6, 69.9, 38.1, 31.6, 25.3, 22.6, 14.0; HRMS (ESI) [M + H]^+^ calcd 257.1654 for C_16_H_21_N_2_O; found 257.1652.

##### Methyl 4-((4*S*,5*R*)-5-((*E*)-2-(3-(1-Hydroxyhexyl)quinoxalin-2-yl)vinyl)-2,2-dimethyl-1,3-dioxolan-4-yl)butanoate (**17**)

Hoveyda–Grubbs II catalyst (22 mg, 0.035 mmol, 10 mol %) was added to a Schlenk tube. Acetonide chain **16** (97 mg, 0.412 mmol, 1.15 equiv) and vinyl quinoxaline **15** (95 mg, 0.358 mmol, 1 equiv) dissolved in dry CH_2_Cl_2_ (1.1 mL) were added, and the reaction was heated to 39 °C and stirred for 96 h. The reaction mixture was concentrated and purified by silica gel column chromatography (6:1 → 1:1 pentane/EtOAc) to give product **17** as a brown oil (41 mg, 26%). *R_f_* = 0.2 (6:1 pentane/EtOAc); ^1^H NMR (500 MHz, CDCl_3_) δ 8.11–8.05 (m, 1H), 8.02 (dd, *J* = 6.0, 2.0 Hz, 1H), 7.76–7.69 (m, 2H), 7.17 (dt, *J* = 15.0, 6.0 Hz, 1H), 7.02–6.94 (m, 1H), 5.15 (d, *J* = 7.0 Hz, 1H), 4.86 (q, *J* = 7.0 Hz, 1H), 4.69–4.62 (2 x d, *J* = 7.0 Hz, 1H), 4.32 (dt, *J* = 15.0, 4.5 Hz, 1H), 3.66 (s, 1H), 3.63 (d, *J* = 2.5 Hz, 2H), 2.40–2.32 (m, 2H), 1.92–1.69 (m, 4H), 1.59 (d, *J* = 2.0 Hz, 3H), 1.55 (dd, *J* = 8.0, 3.0 Hz, 2H), 1.44 (d, *J* = 3.0 Hz, 3H), 1.37–1.25 (m, 6H), 0.89 (dt, *J* = 12.0, 6.0 Hz, 3H); ^13^C NMR spectrum complicated by a mixture of diastereomers (NMR spectra of individual diastereomers are reported vide infra); IR (CHCl_3_) (ν_max_, cm^–1^) 3054, 1733, 1422, 1266; HRMS (ESI) [M + H]^+^ calcd 457.2702 for C_26_H_37_N_2_O_5_; found, 457.2702.

##### (3a*R*,7a*S*)-2,2-Dimethyltetrahydro-4*H*-[1,3]dioxolo[4,5-*c*]pyran-6-ol (**20**)^68^

2-Deoxy-d-ribose (10.0 g, 74.6 mmol, 1 equiv) and pyridinium 4-toluenesulfonate (500 mg, 1.99 mmol, 2.7 mol %) were dissolved in acetone (125 mL). The reaction mixture was stirred for 15 min. 2-Methoxypropene (14.0 mL, 146.19 mmol, 2 equiv) was then added, and the reaction was stirred at room temperature for 18 h. The reaction mixture was filtered using a Buchner funnel, and triethylamine (1.0 mL) was added to the filtrate. The mixture was concentrated, and the crude product was purified by silica gel column chromatography (4:1 → 1:1 pentane/EtOAc) to afford product **20** as a light-yellow oil (7.65 g, 59%). Spectral data were consistent with the literature; *R_f_* = 0.25 (1:1 pentane/EtOAc); ^1^H NMR (400 MHz, CDCl_3_) δ 5.25 (dd, *J* = 7.0, 4.5 Hz, 0.7H), 5.06 (m, 0.3H), 4.47 (dt, *J* = 6.5, 4.0 Hz, 0.7H), 4.40 (dd, *J* = 10.5, 5.0 Hz, 0.3H), 4.19–4.13 (m, 1H), 3.96–3.91 (m, 1H), 3.73–3.66 (m, 1H), 3.23–3.09 (m, 1H), 2.23 (dt, *J* = 15.0, 4.5 Hz, 0.7H), 2.10–2.06 (m, 0.6H), 1.77 (ddd, *J* = 15.0, 7.0, 5.0 Hz, 0.7H), 1.53 (s, 1H), 1.49 (s, 2H), 1.35–1.32 (m, 3H); [α]_D_ = −14.1 (*c* = 0.95 in CHCl_3_) (Lit. [α]_D_ = −18.5 (*c* = 4.2 in CHCl_3_)).

##### Methyl (*E*)-4-((4*S*,5*R*)-5-(Hydroxymethyl)-2,2-dimethyl-1,3-dioxolan-4-yl)but-2-enoate (**21**)^68^

Methyl (triphenylphosphoranylidene)acetate (8.81 g, 26.4 mmol, 1.2 equiv) was dissolved in THF (120 mL). The protected ribose **20** (3.83 g, 21.958 mmol, 1 equiv), dissolved in THF (30 mL), was added followed by benzoic acid (134 mg, 1.099 mmol, 5 mol %). The mixture was heated to reflux and stirred for 4 h. The reaction was allowed to cool to room temperature, filtered through a pad of celite, and then eluted with Et_2_O (80 mL), and the reaction mixture was washed with a saturated aq NH_4_Cl solution (50 mL), H_2_O (50 mL), and brine (50 mL). The organic phase was then dried over MgSO_4_, filtered, and concentrated, and the crude mixture was purified by silica gel column chromatography (4:1 → 3:1 pentane/EtOAc) to afford product **21** as a yellow oil (3.958 g, 78%). Spectral data were consistent with the literature;^68^*R_f_* = 0.30 (1:1 pentane/EtOAc); ^1^H NMR (300 MHz, CDCl_3_) δ 6.97 (dt, *J* = 15.5, 7.0 Hz, 1H), 5.93 (dt, *J* = 15.0, 1.5 Hz, 1H), 4.29 (ddd, *J* = 8.5, 6.0, 5.0 Hz, 1H), 4.20 (dd, *J* = 11.5, 5.5 Hz, 1H), 3.73 (s, 3H), 3.65 (t, *J* = 5.5 Hz, 2H), 2.60–2.37 (m, 2H), 1.47 (s, 3H), 1.36 (s, 3H); ^13^C NMR (101 MHz, CDCl_3_) δ 166.8, 145.0, 123.3, 108.7, 77.6, 75.5, 61.6, 51.7, 32.6, 28.1, 25.4; IR (CH_2_Cl_2_) (ν_max_, cm^–1^) = 3053, 2987, 1725, 1712, 1265, 1217, 1069; [α]_D_ = −28.26 (*c* = 0.65 in CHCl_3_); HRMS (ESI) [M + Na]^+^ calcd 253.1052 for C_11_H_18_O_5_Na; found, 253.1057.

##### Methyl 4-((4*R*, 5*S*)-5-(Hydroxymethyl)-2, 2-dimethyl-1, 3-dioxolan-4-yl) butanoate (**22**)^68^

Alkene **21** (2.469 g, 10.72 mmol, 1 equiv) was dissolved in EtOAc (30 mL). The reaction flask was evacuated and backfilled with N_2_ three times. Then, 10% Pd/C (250 mg, 10 wt %) was added and the flask was evacuated and backfilled with N_2_. The flask was then evacuated and backfilled with H_2_ three times and stirred under a balloon of H_2_ at room temperature for 16 h. The reaction mixture was filtered through a pad of celite, eluted with EtOAc, and concentrated to afford the title compound **22** as a colorless oil (2.327 g, 95%). *R_f_* = 0.32 (1:1 pentane/EtOAc); ^1^H NMR (300 MHz, CDCl_3_) δ 4.15 (m, 2H), 3.66 (s, 3H), 3.60 (d, *J* = 4.0 Hz, 2H), 2.37 (td, *J* = 7.0, 4.5 Hz, 2H), 2.17 (s, 1H), 1.93–1.50 (m, 4H), 1.45 (s, 3H), 1.35 (s, 3H); ^13^C NMR (101 MHz, CDCl_3_) δ 173.8, 108.1, 77.8, 76.6, 61.6, 51.5, 33.6, 28.3, 28.1, 25.4, 22.0; IR (neat) (ν_max_, cm^–1^) 3469, 2881, 1712, 1249, 1216, 1043; [α]_D_ = + 14.48 (*c* = 2.3 in CHCl_3_)^68^ [α]_D_ = +21.8 (*c* = 2.7 in CHCl_3_); HRMS (ESI) [M + Na]^+^ calcd 255.1208 for C_11_H_20_O_5_Na; found, 255.1200.

##### Methyl 4-((4*S*,5*S*)-5-Formyl-2,2-dimethyl-1,3-dioxolan-4-yl)butanoate (**23**)

Alcohol **22** (1.2 g, 5.17 mmol, 1 equiv) was dissolved in CH_2_Cl_2_ (8 mL), and diacetoxyiodobenzene (2.00 g, 6.20 mmol, 1.2 equiv) and TEMPO (202 mg, 1.293 mmol, 0.25 equiv) were added. The reaction was stirred at room temperature for 4 h, after which the solvent, a crude mixture, was purified by silica gel column chromatography (6:1 → 3:1 pentane/EtOAc). Product **23** was isolated as a pale-yellow oil (930 mg, 82%). *R_f_* = 0.53 (1:1 pentane/EtOAc); ^1^H NMR (400 MHz, CDCl_3_) δ 9.63 (d, *J* = 3.0 Hz, 1H), 4.36–4.30 (m, 1H), 4.25 (dd, *J* = 7.0, 3.0 Hz, 1H), 3.66 (s, 3H), 2.35 (t, *J* = 7.0 Hz, 2H), 1.88–1.60 (m, 4H), 1.57 (s, 3H), 1.40 (s, 3H); ^13^C NMR (101 MHz, CDCl_3_) δ 202.1, 173.5, 110.6, 81.9, 78.2, 51.5, 33.5, 29.1, 27.6, 25.3, 21.9; IR (neat) (ν_max_, cm^–1^) 3456, 1733, 1639, 1256, 909, 737; [α]_D_ = −14.5 (*c* = 0.94 in CHCl_3_); HRMS (ESI) [M + Na]^+^ calcd 253.1052 for C_11_H_18_O_5_Na; found, 253.1050.

##### Dichloromethyl Pinacol Boronate (**24**)^40^

CH_2_Cl_2_ (1.77 mL, 27.72 mmol, 1 equiv) was added to THF (45 mL) and cooled to −100 °C. *n*BuLi (9.98 mL of 2.5 M sol., 24.95 mmol, 0.9 equiv) was added dropwise over 45 min. The reaction mixture was stirred for a further 30 min at −100 °C. Trimethylborate (3.09 mL, 27.718 mmol, 1 equiv) was added in one portion, and the reaction mixture was stirred for 30 min. HCl (5 mL of 5 M sol.) was added, and the reaction mixture was stirred while warming to room temperature. The reaction mixture was diluted with H_2_O (25 mL), extracted with Et_2_O (3 × 50 mL), and concentrated to a yellow oil. This was dissolved in toluene (50 mL), pinacol (3.275 g, 27.718, 1 equiv) was added, and the reaction mixture was refluxed for 72 h. The reaction mixture was concentrated and purified by vacuum distillation, affording the purified product **24** as a colorless oil (3.7 g, 70%). Spectral data matched those previously reported;^69^ Bp = 94–96 °C at 0.6 mbar; ^1^H NMR (400 MHz, CDCl_3_) δ 5.32 (s, 1H), 1.31 (s, 12H).

##### Methyl 4-((4*S*,5*R*)-2,2-Dimethyl-5-((*E*)-2-(4,4,5,5-tetramethyl-1,3,2-dioxaborolan-2-yl)vinyl)-1,3-dioxolan-4-yl)butanoate (**18**)

CrCl_2_ (3.2 g, 26.06 mmol, 8 equiv) was dissolved in dry THF (20 mL) in a Schlenk tube. Aldehyde **23** (750 mg, 3.26 mmol, 1 equiv) and boronic ester **24** (1.375 mg, 6.514 mmol, 2.0 equiv) were dissolved in THF (8 mL) and added to the reaction flask. LiI (1.744 mg, 13.028 mmol, 4.0 equiv), dissolved in THF (5 mL), was added slowly. The Schlenk tube was wrapped in a tin foil and stirred vigorously for 12 h with the reaction mixture turning brown in color. The mixture was poured onto ice-water (50 mL) and turned green in color. This was extracted with EtOAc (3 × 40 mL), and the extracts were washed with brine (50 mL), dried over Na_2_SO_4_, filtered, and concentrated. The crude product was purified by silica gel column chromatography (6:1 pentane: EtOAc → 2:1 pentane/EtOAc) affording product **18** as a pale-yellow oil (780 mg, 68%). *R_f_* = 0.46 (6:1 pentane/EtOAc); ^1^H NMR (500 MHz, CDCl_3_) δ 6.47 (dd, *J* = 18.0, 6.8 Hz, 1H), 5.69 (d, *J* = 18.0 Hz, 1H), 4.47 (td, *J* = 6.8, 1.0 Hz, 1H), 4.18–4.12 (m, 1H), 3.64 (s, 3H), 2.33 (td, *J* = 7.5, 1.5 Hz, 2H), 1.83–1.59 (m, 2H), 1.46 (s, 3H), 1.46–1.36 (m, 2H), 1.33 (s, 3H), 1.24 (s, 12H). ^13^C NMR (101 MHz, CDCl_3_) δ 172.8, 147.0, 122.0, 107.5, 82.3, 79.4, 77.0, 50.5, 32.7, 28.8, 27.1, 24.6, 23.8, 23.7, 20.8; IR (CHCl_3_) (ν_max_, cm^–1^) 3435, 3020, 1734, 1648, 1216; [α]_D_ = −1.72 (*c* = 1.0 in CHCl_3_); HRMS (ESI) [M + Na]^+^ calcd 377.2111 for C_18_H_31_O_6_Na; found, 377.2108.

##### Methyl 4-((4*S*,5*R*)-5-((*E*)-2-(3-((*R*)-1-Hydroxyhexyl)quinoxalin-2-yl)vinyl)-2,2-dimethyl-1,3-dioxolan-4-yl)butanoate ((1*R*)-**17**)

*Bis*(benzonitrile)Pd(II)chloride (10 mg, 0.0261 mmol, 0.17 equiv) and dppb (18 mg, 0.0422 mmol, 0.28 equiv) were dissolved in toluene (1 mL) and stirred at room temperature for 30 min to give a creamy orange solution. Quinoxaline alcohol (1*R*)-**14** (40 mg, 0.151 mmol, 1 equiv), dissolved in toluene (1 mL), and boronic ester **18** (62 mg, 0.180 mmol, 1.2 equiv), dissolved in toluene (1 mL), were added followed by EtOH (0.05 mL) and Na_2_CO_3_ (0.17 mL of a 1 M aq solution, 0.177 mmol, 1.2 equiv). The reaction mixture was heated to 110 °C and stirred for 18 h, after which it was diluted with H_2_O (20 mL) and extracted with EtOAc (3 × 15 mL), and the extracts were then washed with brine (20 mL), dried with MgSO_4_, filtered, and concentrated. The crude reaction mixture was purified by silica gel column chromatography (10:1 → 2:1 pentane/EtOAc) to give product (1*R*)-**17** as a pale-yellow oil (33 mg, 48%). *R_f_* = 0.2 (6:1 pentane/EtOAc); ^1^H NMR (400 MHz, CDCl_3_) δ 8.11–7.94 (m, 2H), 7.70 (m, 2H), 7.14 (dd, *J* = 15.0, 6.0 Hz, 1H), 6.97 (d, *J* = 15.0 Hz, 1H), 5.16–5.10 (m, 1H), 4.84 (dd, *J* = 11.0, 5.5 Hz, 1H), 4.64 (d, *J* = 6.5 Hz, 1H), 4.30 (dt, *J* = 8.5, 5.5 Hz, 1H), 3.63 (s, 3H), 2.43–2.23 (m, 2H), 1.94–1.80 (m, 2H), 1.76–1.63 (m, 2H), 1.57 (s, 3H), 1.42 (s, 3H), 1.34–1.21 (m, 8H), 0.85 (t, *J* = 7 Hz, 3H). ^13^C NMR (101 MHz, CDCl_3_) δ 173.7, 155.1, 146.9, 141.8, 139.8, 135.9, 129.7, 129.7, 129.1, 128.2, 125.3, 108.7, 78.3, 78.3, 69.8, 51.5, 38.1, 33.7, 31.5, 30.1, 28.2, 25.6, 25.2, 22.6, 21.8, 14.0; IR (CHCl_3_) (ν_max_, cm^–1^) 3054, 1733, 1422, 1266; [α]_D_ = +2.21 (*c* = 0.8 in CHCl_3_); HRMS (ESI) [M + H]^+^ calcd 457.2702 for C_26_H_37_N_2_O_5_; found, 457.2706.

##### Methyl 4-((4*S*,5*R*)-5-((*E*)-2-(3-((*S*)-1-Hydroxyhexyl)quinoxalin-2-yl)vinyl)-2,2-dimethyl-1,3-dioxolan-4-yl)butanoate ((1*S*)-**17**)

*Bis*(benzonitrile)Pd(II)chloride (10 mg, 0.0261 mmol, 0.17 equiv) and dppb (18 mg, 0.0422 mmol, 0.28 equiv) were dissolved in toluene (1 mL) and stirred at room temperature for 30 min to give a creamy orange solution. Quinoxaline alcohol (1*S*)-**14** (39 mg, 0.147 mmol, 1 equiv), dissolved in toluene (1 mL), and boronic ester **18** (70 mg, 0.198 mmol, 1.3 equiv), dissolved in toluene (1 mL), were added followed by EtOH (0.05 mL) and Na_2_CO_3_ (0.17 mL of a 1 M aq solution, 0.177 mmol, 1.2 equiv). The reaction mixture was heated to 110 °C and stirred for 14 h after which it was diluted with H_2_O (20 mL) and extracted with EtOAc (3 × 20 mL), and the extracts were then washed with brine (20 mL), dried with MgSO_4_, filtered, and concentrated to give a crude mixture, which was purified by silica gel column chromatography (10:1 → 2:1 pentane/EtOAc) to give product (1*S*)-**17** as a pale-yellow oil (27 mg, 40%). *R_f_* = 0.2 (6:1 pentane/EtOAc); ^1^H NMR (500 MHz, CDCl_3_) δ 8.04–7.92 (m, 2H), 7.70–7.61 (m, 2H), 7.10 (dd, *J* = 15.0, 6.0 Hz, 1H), 6.90 (d, *J* = 15.0 Hz, 1H), 5.08 (m, 1H), 4.80 (t, *J* = 6.0 Hz, 1H), 4.62 (d, *J* = 7.0 Hz, 1H), 4.25 (dd, *J* = 13.5, 6.0 Hz, 1H), 3.55 (s, 3H), 2.33–2.24 (m, 2H), 1.85–1.78 (m, 2H), 1.70–1.63 (m, 1H), 1.53 (s, 3H), 1.51–1.47 (m, 4H), 1.37 (s, 3H), 1.33–1.20 (m, 5H), 0.81 (t, *J* = 6.8 Hz, 3H); ^13^C NMR (101 MHz, CDCl_3_) δ 174.3, 155.3, 147.3, 141.9, 140.0, 137.9, 130.0, 129.9, 129.1, 128.5, 125.4, 75.2, 74.1, 70.1, 51.8, 38.1, 33.8, 31.8, 31.5, 25.3, 22.7, 21.2, 14.2; IR (CHCl_3_) (ν_max_, cm^–1^) 3054, 1733, 1422, 1266; [α]_D_ = +83.31 (*c* = 0.85 in CHCl_3_); HRMS (ESI) [M + H]^+^ calcd 457.2702 for C_26_H_37_N_2_O_5_; found, 457.2706.

##### Methyl (5*S*,6*R*,*E*)-5,6-Dihydroxy-8-(3-((*S*)-1-hydroxyhexyl)quinoxalin-2-yl)oct-7-enoate ((1*S*)-**6**)

Acetonide (1*S*)-**17** (20 mg, 0.0438 mmol, 1 equiv) was dissolved in MeOH (0.5 mL), camphorsulfonic acid (9 mg, 0.0387 mmol, 0.88 equiv) was added, and the reaction mixture was stirred at room temperature for 3 h. The mixture was diluted with Et_2_O (20 mL), washed with H_2_O (20 mL) and brine (20 mL), and the organic layer was dried with MgSO_4_, filtered, and concentrated. The crude product was purified by preparative thin-layer chromatography (96:4 CH_2_Cl_2_/MeOH). Product (1*S*)-**6** was isolated as a yellow oil (15 mg, 83%). *R_f_* = 0.36 (96:4 CH_2_Cl_2_/MeOH); ^1^H NMR (500 MHz, CDCl_3_) δ 8.07–7.99 (m, 2H), 7.74–7.69 (m, 2H), 7.22 (dd, *J* = 15.0, 5.5 Hz, 1H), 7.04 (dd, *J* = 15.0, 1.5 Hz, 1H), 5.16 (dd, *J* = 7.5, 3.0 Hz, 1H), 4.51–4.47 (m, 1H), 3.85 (dt, *J* = 8.5, 4.0 Hz, 1H), 3.66 (s, 3H), 2.37 (td, *J* = 7.0, 2.0 Hz, 2H), 1.93–1.85 (m, 2H), 1.79–1.72 (m, 1H), 1.62–1.53 (m, 4H), 1.35–1.24 (m, 6H), 0.87 (t, *J* = 7.0 Hz, 3H); ^13^C NMR (101 MHz, CDCl_3_) δ 174.4, 155.3, 147.3, 141.8, 140.0, 137.9, 130.0, 129.9, 129.1, 128.5, 125.3, 75.2, 74.1, 70.2, 51.8, 38.1, 33.8, 31.8, 31.4, 25.3, 22.7, 21.2, 14.2; IR (CHCl_3_) (ν_max_, cm^–1^) 3434, 3054, 1734, 1422, 1265; [α]_D_ = −14.33 (*c* = 0.75 in CHCl_3_); HRMS (ESI) [M + Na]^+^ calcd 439.2209 for C_23_H_32_O_5_Na; found, 439.2213.

##### Methyl (5*S*,6*R*,*E*)-5,6-Dihydroxy-8-(3-((*R*)-1-hydroxyhexyl)quinoxalin-2-yl)oct-7-enoate ((1*R*)-**6**)

Acetonide (1*R*)-**17** (25 mg, 0.055 mmol, 1 equiv) was dissolved in MeOH (1.5 mL), camphorsulfonic acid (14 mg, 0.060 mmol, 1.1 equiv) was added, and the reaction mixture was stirred at room temperature for 3 h. The mixture was concentrated and purified by preparative thin-layer chromatography (96:4 CH_2_Cl_2_/MeOH). Product (1*R*)-**6** was isolated as a yellow oil (15 mg, 65%). *R_f_* = 0.36 (96:4 CH_2_Cl_2_/MeOH); ^1^H NMR (400 MHz, CDCl_3_) δ 8.07–7.95 (m, 2H), 7.75–7.67 (m, 2H), 7.25–7.19 (dd, *J* =15.0, 6 Hz, 1H), 7.01 (dd, *J* = 15.0, 1.5 Hz, 1H), 5.18–5.10 (m, 1H), 4.64 (d, *J* = 7.0 Hz, 1H), 4.49–4.44 (m, 1H), 3.89–3.82 (m, 1H), 3.66 (s, 3H), 2.75 (s, 1H), 2.63 (s, 1H), 2.38 (t, *J* = 7.5 Hz, 2H), 1.95–1.81 (m, 2H), 1.81–1.67 (m, 2H), 1.64–1.51 (m, 4H), 1.33–1.25 (m, 4H), 0.86 (t, *J* = 7.0 Hz, 3H); ^13^C NMR (101 MHz, CDCl_3_) δ 174.2, 155.1, 147.1, 141.6, 139.9, 137.7, 129.8, 129.7, 128.9, 128.3, 125.3, 75.1, 74.0, 69.9, 51.6, 37.9, 33.6, 31.6, 31.5, 25.1, 22.6, 21.1, 14.0; IR (CHCl_3_) (ν_max_, cm^–1^) 3434, 3054, 1734, 1422, 1265; [α]_D_ = +36.74 (*c* = 0.7 in CHCl_3_); HRMS (ESI) [M]^+^ calcd 417.2389 for C_23_H_32_O_5_; found, 417.2371.

##### 1-(3-Chloroquinoxalin-2-yl)butan-1-one (**27**)

2-Chloroquinoxaline (300 mg, 1.82 mmol, 1 equiv) was dissolved in EtOAc (14 mL). Butyraldehyde (0.66 mL, 7.29 mmol, 4 equiv) and TMSN_3_ (0.48 mL, 3.65 mmol, 2 equiv) were added. PhI(OCOCF_3_)_2_ (1.57 g, 3.65 mmol, 2 equiv) was added portionwise over 15 min, and the mixture turned orange in color. The mixture was stirred at room temperature for 2 h. Further, TMSN_3_ (0.48 mL, 3.65 mmol, 2 equiv) and PhI(OCOCF_3_)_2_ (1.57 g, 3.65 mmol, 2 equiv) were added, and the reaction was stirred for 16 h. Triethylamine (2 mL) was added dropwise, and the mixture was stirred for 10 min. The reaction mixture was concentrated, and the crude mixture was purified by silica gel column chromatography (50:1 cyclohexane/EtOAc) to afford product **27** as a yellow solid (234 mg, 57%). *R_f_* = 0.42 (10:1 cyclohexane/EtOAc); mp = 33–36 °C; ^1^H NMR (400 MHz, CDCl_3_) δ 8.18–8.11 (m, 1H), 8.09–8.03 (m, 1H), 7.86 (m, 2H), 3.19 (t, *J* = 7.0 Hz, 2H), 1.87–1.78 (m, 2H), 1.06 (t, *J* = 7.5 Hz, 3H); ^13^C NMR (101 MHz, CDCl_3_) δ 200.6, 148.5, 143.7, 142.3, 139.5, 132.6, 130.8, 129.6, 128.3, 42.3, 17.1, 13.7; IR (CHCl_3_) (ν_max_, cm^–1^) 2983, 1710, 1260; HRMS (ESI) [M]^+^ calcd 234.0560 for C_12_H_11_N_2_O^35^Cl; found 234.0560.

##### (*R*)-1-(3-Bromoquinoxalin-2-yl)butan-1-ol ((1*R*)-**29**)

Quinoxaline ketone **27** (107 mg, 0.439 mmol, 1 equiv) was dissolved in *^i^*PrOH (2.5 mL). RuCl_2_[(*S*)-xylBinap][(*S*)-DAIPEN] (26 mg, 0.0212 mmol, 0.048 mmol), *^t^*BuOK (12 mg, 0.107 mmol, 0.24 equiv), and tri*iso*propyl borate (0.02 mL) were added, and the reaction mixture was stirred under 25 bar of H_2_ for 18 h. The mixture was concentrated and purified by silica gel column chromatography (20:1 → 10:1 cyclohexane/EtOAc), and product (1*R*)-**29** was isolated as a pale-yellow solid (75 mg, 69%). *R_f_* = 0.31 (20:1 cyclohexane/EtOAc); mp = 100–104 °C; ^1^H NMR (400 MHz, CDCl_3_) δ 8.09–8.04 (m, 1H), 8.04–7.99 (m, 1H), 7.79–7.73 (m, 2H), 5.19 (td, *J* = 8.0, 3.0 Hz, 1H), 4.10 (d, *J* = 8.0, 1H), 2.04–1.94 (m, 1H), 1.65–1.54 (m, 3H), 0.98 (t, *J* = 7.0 Hz, 3H); ^13^C NMR (101 MHz, CDCl_3_) δ 155.9, 145.3, 141.7, 139.8, 130.8, 130.6, 128.5, 128.4, 70.3, 39.2, 18.9, 13.9; IR (CHCl_3_) (ν_max_, cm^–1^) 3449, 3020, 1641, 1050; [α]_D_ = −41.16 (*c* = 2.0 in CHCl_3_); *ee* = 98% as determined by SFC using a Chiralpak IC column (CO_2_/MeCN, gradient 99:1 0–1 min and then gradient to 60:40 until 5 min, 3 mL/min), *R*_t_ = 3.02 min (*S*)-enantiomer, 3.45 min (*R*)-enantiomer.

##### (*S*)-1-(3-Bromoquinoxalin-2-yl)butan-1-ol ((1*S*)-**29**)

Quinoxaline ketone **27** (107 mg, 0.439 mmol, 1 equiv) was dissolved in ^*i*^PrOH (2.5 mL). RuCl_2_[(*R*)-xylBinap][(*R*)-DAIPEN] (26 mg, 0.0212 mmol, 0.048 mmol), *^t^*BuOK (12 mg, 0.107 mmol, 0.24 equiv), and tri*iso*propyl borate (0.02 mL) were added, and the reaction mixture was stirred under 25 bar of H_2_ for 18 h. The mixture was concentrated and purified by silica gel column chromatography (20:1 → 10:1 cyclohexane/EtOAc), and product (1*S*)-**29** was isolated as a pale-yellow solid (75 mg, 69%). [α]_D_ = −41.16 (*c* = 2 in CHCl_3_); *ee* = 98% as determined by SFC using a Chiralpak IC column (CO_2_:MeCN, gradient 99:1 0–1 min and then gradient to 60:40 until 5 min, 3 mL/min), *R*_t_ = 3.02 min (*S*)-enantiomer, 3.45 min (*R*)-enantiomer; identical in all other physical data to the previously prepared (1*R*)-enantiomer.

##### Methyl 4-((4*S*,5*R*)-5-((*E*)-2-(3-((*R*)-1-Hydroxybutyl)quinoxalin-2-yl)vinyl)-2,2-dimethyl-1,3-dioxolan-4-yl)butanoate ((1*R*)-**31**)

*Bis*(benzonitrile)Pd(II)chloride (18 mg, 0.048 mmol, 0.15 equiv) and dppb (34 mg, 0.079 mmol, 0.25 equiv) were dissolved in toluene (1 mL) and stirred at room temperature for 30 min to give a creamy orange solution. Quinoxaline alcohol (1*R*)-**29** (75 mg, 0.317 mmol, 1 equiv), dissolved in toluene (1 mL), and boronic ester **18** (130 mg, 0.367 mmol, 1.16 equiv), dissolved in toluene (1 mL), were added followed by EtOH (0.09 mL) and Na_2_CO_3_ (0.37 mL of a 1 M aq solution, 0.37 mmol, 1.15 equiv). The reaction mixture was heated to 110 °C and stirred for 64 h. It was diluted with H_2_O (10 mL) and extracted with EtOAc (3 × 10 mL), and the extracts were washed with brine (15 mL), dried with MgSO_4_, filtered, and concentrated. The crude product was purified by silica gel column chromatography (10:1 → 3:1 cyclohexane/EtOAc) to give product (1*R*)-**31** as a pale-yellow oil (36 mg, 27%). *R_f_* = 0.15 (6:1 pentane/EtOAc); ^1^H NMR (400 MHz, CDCl_3_) δ 8.09–7.99 (m, 2H), 7.75–7.68 (m, 2H), 7.16 (dd, *J* = 15.0, 6.0 Hz, 1H), 6.99 (d, *J* = 15.0 Hz, 1H), 5.16 (td, *J* = 7.0, 3.0 Hz, 1H), 4.85 (t, *J* = 6.0 Hz, 1H), 4.62 (d, *J* = 7.0 Hz, 1H), 4.32 (dt, *J* = 8.5, 6.0 Hz, 1H), 3.63 (s, 3H), 2.39–2.32 (m, 2H), 1.91–1.72 (m, 3H), 1.62–1.50 (m, 8H), 1.44 (s, 3H), 0.98 (t, *J* = 7.0 Hz, 3H); ^13^C NMR (101 MHz, CDCl_3_) δ 173.9, 155.3, 147.0, 142.0, 140.1, 136.1, 129.9, 129.8, 129.3, 128.4, 125.5, 108.9, 78.5, 78.5, 69.7, 51.7, 40.4, 33.8, 30.3, 28.4, 25.8, 22.0, 18.9, 14.0; IR (CHCl_3_) (ν_max_, cm^–1^) 3316, 2960, 1729, 1650, 1216; [α]_D_ = +12.14 (*c* = 1.0 in CHCl_3_); HRMS (ESI) [M + H]^+^ calcd 429.2389 for C_24_H_33_N_2_O_5_; found 429.2396.

##### Methyl 4-((4*S*,5*R*)-5-((*E*)-2-(3-((*S*)-1-Hydroxybutyl)quinoxalin-2-yl)vinyl)-2,2-dimethyl-1,3-dioxolan-4-yl)butanoate ((1*S*)-**31**)

*Bis*(benzonitrile)Pd(II)chloride (15 mg, 0.038 mmol, 0.15 equiv) and dppb (27 mg, 0.063 mmol, 0.25 equiv) were dissolved in toluene (1 mL) and stirred at room temperature for 30 min to give a creamy orange solution. Quinoxaline alcohol (1*S*)-**29** (60 mg, 0.253 mmol, 1 equiv), dissolved in toluene (1 mL), and boronic ester **18** (113 mg, 0.319 mmol, 1.26 equiv), dissolved in toluene (1 mL), were added followed by EtOH (0.08 mL) and Na_2_CO_3_ (0.29 mL of a 1 M aq solution, 0.29 mmol, 1.15 equiv). The reaction mixture was heated to 110 °C and stirred for 48 h. The reaction mixture was diluted with H_2_O (10 mL) and extracted with EtOAc (3 × 10 mL), and the extracts were washed with brine (15 mL), dried with MgSO_4_, filtered, and concentrated. The crude product was purified by silica gel column chromatography (6:1 → 3:1 cyclohexane/EtOAc) to give product (1*S*)-**31** as a pale-yellow oil (30 mg, 28%). *R_f_* = 0.15 (6:1 pentane/EtOAc); ^1^H NMR (400 MHz, CDCl_3_) δ 8.10–7.98 (m, 2H), 7.77–7.67 (m, 2H), 7.17 (dd, *J* = 15.0, 5.5 Hz, 1H), 6.98 (dd, *J* = 15.0, 1.5 Hz, 1H), 5.21–5.13 (m, 1H), 4.86 (td, *J* = 6.5, 1.0 Hz, 1H), 4.69 (d, *J* = 7.0 Hz, 1H), 4.32 (ddd, *J* = 8.5, 6.5, 5.5 Hz, 1H), 3.62 (s, 3H), 2.40–2.31 (m, 2H), 1.92–1.81 (m, 2H), 1.78–1.68 (m, 1H), 1.59 (s, 3H), 1.59–1.50 (m, 5H), 1.44 (s, 3H), 0.96 (t, *J* = 7.0 Hz, 3H); ^13^C NMR (101 MHz, CDCl_3_) δ 173.7, 155.1, 146.9, 141.8, 139.8, 135.7, 129.7, 129.7, 129.1, 128.2, 125.1, 108.7, 78.3, 78.3, 69.6, 51.5, 40.3, 33.7, 30.2, 28.1, 25.6, 21.8, 18.9, 13.9; IR (CHCl_3_) (ν_max_, cm^–1^) 3316, 2960, 1729, 1650, 1216; [α]_D_ = −94.53 (*c* = 1.5 in CHCl_3_); HRMS (ESI) [M + H]^+^ calcd 429.2389 for C_24_H_33_N_2_O_5_; found, 429.2392.

##### Methyl (5*S*,6*R*,*E*)-5,6-Dihydroxy-8-(3-((*R*)-1-hydroxybutyl)quinoxalin-2-yl)oct-7-enoate ((1*R*)-**7**)

Acetonide (1*R*)-**31** (29 mg, 0.068 mmol, 1 equiv) was dissolved in MeOH (1.5 mL), camphorsulfonic acid (16 mg, 0.068 mmol, 1 equiv) was added, and the reaction mixture was stirred at room temperature for 3 h. The mixture was diluted with Et_2_O (15 mL) and washed with H_2_O (10 mL) and brine (10 mL), and the organic layer was dried with MgSO_4_, filtered, and concentrated. The crude product was purified by preparative thin-layer chromatography (96:4 CH_2_Cl_2_/MeOH). Product (1*R*)-**7** was isolated as a yellow oil (12 mg, 46%). *R_f_* = 0.41 (96:4 CH_2_Cl_2_/MeOH); ^1^H NMR (400 MHz, CDCl_3_) δ 8.05–7.93 (m, 2H), 7.72–7.65 (m, 2H), 7.22 (dd, *J* = 15.0, 5.5 Hz, 1H), 7.01 (dd, *J* = 15.0, 1.5 Hz, 1H), 5.14 (dd, J = 7.5, 6.5 Hz, 1H), 4.65 (d, *J* = 6.5 Hz, 1H), 4.49–4.42 (m, 1H), 3.88–3.82 (m, 1H), 3.65 (s, 3H), 2.94 (s, 1H), 2.79 (s, 1H), 2.37 (t, *J* = 7.2 Hz, 2H), 2.02–1.40 (m, 8H), 0.94 (t, *J* = 7.1 Hz, 3H); ^13^C NMR (101 MHz, CDCl_3_) δ 174.3, 155.3, 147.3, 141.8, 140.0, 138.1, 130.0, 129.9, 129.1, 128.4, 125.4, 75.3, 74.2, 69.8, 51.8, 40.1, 33.8, 31.7, 21.3, 18.8, 14.0; IR (CHCl_3_) (ν_max_, cm^–1^) 3020, 2976, 1734, 1216, 1095; [α]_D_ = +27.63 (*c* = 1.2 in CHCl_3_); HRMS (ESI) [M + H]^+^ calcd 389.2076 for C_21_H_29_N_2_O_5_; found, 389.2096.

##### Methyl (5*S*,6*R*,*E*)-5,6-Dihydroxy-8-(3-((*S*)-1-hydroxybutyl)quinoxalin-2-yl)oct-7-enoate ((1*S*)-**7**)

Acetonide (1*S*)-**31** (28 mg, 0.065 mmol, 1 equiv) was dissolved in MeOH (1.5 mL), camphorsulfonic acid (16 mg, 0.069 mmol, 1.05 equiv) was added, and the reaction mixture was stirred at room temperature for 16 h The mixture was diluted with Et_2_O (15 mL) and washed with H_2_O (10 mL) and brine (10 mL), and the organic layer was dried with MgSO_4_, filtered, and concentrated. The crude product was purified by preparative thin-layer chromatography (96:4 CH_2_Cl_2_/MeOH). Product (1*S*)-**7** was isolated as a yellow oil (14 mg, 56%). *R_f_* = 0.41 (96:4 CH_2_Cl_2_/MeOH); ^1^H NMR (400 MHz, CDCl_3_) δ 8.07–7.95 (m, 2H), 7.75–7.64 (m, 2H), 7.21 (dd, *J* = 15.5, 5.5 Hz, 1H), 7.04 (d, *J* = 15.5 Hz, 1H), 5.22–5.10 (m, 1H), 4.72–4.61 (m, 1H), 4.53–4.45 (m, 1H), 3.90–3.81 (m, 1H), 3.65 (s, 3H), 2.93–2.55 (m, 2H), 2.37 (t, *J* = 7.0 Hz, 2H), 1.96–1.67 (m, 4H), 1.59–1.48 (m, 4H), 0.95 (t, *J* = 7.0 Hz, 3H); ^13^C NMR (101 MHz, CDCl_3_) δ 174.2, 155.2, 147.2, 141.6, 139.8, 137.8, 129.8, 129.7, 128.9, 128.3, 125.2, 75.1, 74.0, 69.8, 51.6, 40.0, 33.6, 31.3, 21.1, 18.7, 13.9; IR (CHCl_3_) (ν_max_, cm^–1^) 3020, 2976, 1734, 1216, 1095; [α]_D_ = −60.02 (*c* = 1.4 in CHCl_3_); HRMS (ESI) [M + H]^+^ calcd 389.2076 for C_21_H_29_N_2_O_5_; found 389.2076.

##### 1-(3-Bromoquinoxalin-2-yl)octan-1-one (**28**)

2-Bromoquinoxaline (350 mg, 1.67 mmol, 1 equiv) was dissolved in EtOAc (16 mL). Octanal (1.05 mL, 6.715 mmol, 4 equiv) and TMSN_3_ (0.44 mL, 6.698 mmol, 2 equiv) were added. PhI(OCOCF_3_)_2_ (1.44 g, 6.698 mmol, 2 equiv) was added portionwise over 10 min, and the mixture turned orange in color. The mixture was stirred at room temperature for 2 h. Further, TMSN_3_ (0.44 mL, 6.698 mmol, 2 equiv) and PhI(OCOCF_3_)_2_ (1.44 g, 6.698 mmol, 2 equiv) were added and the reaction was stirred for 18 h. Triethylamine (2 mL) was added dropwise, and the mixture was stirred for 15 min. The reaction mixture was concentrated and the crude mixture was purified by silica gel column chromatography (50:1 pentane/EtOAc) to afford product **28** as a yellow solid (259 mg, 61%). *R_f_* = 0.38 (10:1 cyclohexane/EtOAc); mp = 43–48 °C; ^1^H NMR (400 MHz, CDCl_3_) δ 8.16–8.04 (m, 2H), 7.90–7.81 (m, 2H), 3.18 (t, *J* = 7.5 Hz, 2H), 1.83–1.75 (m, 2H), 1.44–1.28 (m, 8H), 0.89 (t, *J* = 7.0 Hz, 3H); ^13^C NMR (101 MHz, CDCl_3_) δ 201.5, 149.8, 143.3, 139.6, 134.8, 132.6, 131.2, 129.8, 128.61, 40.7, 31.8, 29.3, 29.2, 23.7, 22.8, 14.2; IR (CHCl_3_) (ν_max_, cm^–1^) 3429, 1708, 1560; HRMS (ESI) [M + H]^+^ calcd 335.0759 for C_16_H_20_N_2_O^79^Br; found 335.0759.

##### (*R*)-1-(3-Bromoquinoxalin-2-yl)octan-1-ol ((1*R*)-**30**)

Quinoxaline ketone **28** (90 mg, 0.268 mmol, 1 equiv) was dissolved in ^*i*^PrOH (3 mL). RuCl_2_[(*S*)-xylBinap][(*S*)-DAIPEN] (16 mg, 0.0134 mmol, 0.05 mmol), *^t^*BuOK (8 mg, 0.067 mmol, 0.25 equiv), and tri*iso*propyl borate (0.02 mL) were added, and the reaction mixture was stirred under 25 bar of H_2_ for 18 h. The mixture was concentrated and purified by silica gel column chromatography (10:1 pentane/EtOAc), and product (1*R*)-**30** was isolated as a pale-yellow solid (37 mg, 41%). Mp = 48–54 °C; *R_f_* = 0.41 (10:1 cyclohexane/EtOAc); ^1^H NMR (300 MHz, CDCl_3_) δ 8.15–8.04 (m, 2H), 7.88–7.76 (m, 2H), 5.24–5.15 (m, 1H), 4.04 (d, *J* = 8.5 Hz, 1H), 2.13–2.01 (m, 1H), 1.65–1.57 (m, 3H), 1.29 (s, 8H), 0.89 (t, *J* = 6.5 Hz, 3H); ^13^C NMR (101 MHz, CDCl_3_) δ 157.0, 142.5, 139.7, 138.1, 130.7, 130.7, 128.5, 128.3, 71.4, 37.3, 31.8, 29.3, 29.2, 25.7, 22.6, 14.1; IR (CHCl_3_) (ν_max_, cm^–1^) 3417, 1641; [α]_D_ = +12.48 (*c* = 1.7 in CHCl_3_); [M + H]^+^ calcd 337.0915 for C_16_H_22_N_2_O^79^Br; found 337.0900; *ee* = 99% as determined by SFC using a Chiralpak IC column (CO_2_/MeCN, gradient 99:1 0–1 min and then gradient to 60:40 until 5 min, 3 mL/min), *R*_t_ = 3.43 min (*S*)-enantiomer, 4.18 min (*R*)-enantiomer.

##### (*S*)-1-(3-Bromoquinoxalin-2-yl)octan-1-ol ((1*S*)-**30**)

Quinoxaline ketone **28** (100 mg, 0.298 mmol, 1 equiv) was dissolved in ^*i*^PrOH (3 mL). RuCl_2_[(*S*)-xylBinap][(*S*)-DAIPEN] (18 mg, 0.0145 mmol, 0.05 mmol), *^t^*BuOK (8 mg, 0.0745 mmol, 0.25 equiv), and tri*iso*propyl borate (0.02 mL) were added, and the reaction mixture was stirred under 25 bar of H_2_ for 18 h. The mixture was concentrated and purified by silica gel column chromatography (10:1 pentane/EtOAc), and product (1*S*)-**30** was isolated as a pale-yellow solid (40 mg, 40%). [α]_D_ = −21.63 (*c* = 0.5 in CHCl_3_); *ee* = 98% as determined by SFC using a Chiralpak IC column (CO_2_/MeCN, gradient 99:1 0–1 min and then gradient to 60:40 until 5 min, 3 mL/min), *R*_t_ = 3.43 min (*S*)-enantiomer, 4.18 min (*R*)-enantiomer; identical in all other physical data to the previously prepared (1*R*)-enantiomer.

##### Methyl 4-((4*S*,5*R*)-5-((*E*)-2-(3-((*R*)-1-Hydroxyoctyl)quinoxalin-2-yl)vinyl)-2,2-dimethyl-1,3-dioxolan-4-yl)butanoate ((1*R*)-**32**)

*Bis*(benzonitrile)Pd(II)chloride (11 mg, 0.029 mmol, 0.15 equiv) and dppb (21 mg, 0.048 mmol, 0.25 equiv) were dissolved in toluene (1 mL) and stirred at room temperature for 30 min to give a creamy orange solution. Quinoxaline alcohol (1*R*)-**30** (65 mg, 0.193 mmol, 1 equiv), dissolved in toluene (1 mL), and boronic ester **18** (81 mg, 0.231 mmol, 1.2 equiv), dissolved in toluene (1 mL), were added followed by EtOH (0.09 mL) and Na_2_CO_3_ (0.22 mL of a 1 M aq solution, 0.222 mmol, 1.15 equiv). The reaction mixture was heated to 110 °C and stirred for 46 h. The reaction mixture was diluted with H_2_O (10 mL) and extracted with EtOAc (3 × 10 mL), and the extracts were washed with brine (15 mL), dried with MgSO_4_, filtered, and concentrated. The crude product was purified by silica gel column chromatography (10:1 → 6:1 pentane/EtOAc) to give product (1*R*)-**32** as a pale-yellow oil (38 mg, 40%). *R_f_* = 0.19 (6:1 pentane/EtOAc); ^1^H NMR (400 MHz, CDCl_3_) δ 8.10–7.99 (m, 2H), 7.77–7.69 (m, 2H), 7.16 (dd, *J* = 15.0, 6.0 Hz, 1H), 6.99 (dd, *J* = 15.0, 1.0 Hz, 1H), 5.14 (td, *J* = 7.5, 3.0 Hz, 1H), 4.85 (td, *J* = 6.0, 1.0 Hz, 1H), 4.61 (d, *J* = 7.5 Hz, 1H), 4.31 (ddd, *J* = 9.0, 6.0, 5.0 Hz, 1H), 3.63 (s, 3H), 2.41–2.30 (m, 2H), 1.94–1.81 (m, 2H), 1.78–1.68 (m, 1H), 1.59 (s, 3H), 1.58–1.52 (m, 4H), 1.44 (s, 3H), 1.37–1.19 (m, 9H), 0.87 (t, *J* = 7.0 Hz, 3H); ^13^C NMR (101 MHz, CDCl_3_) δ 173.9, 155.3, 147.0, 142.0, 140.0, 136.1, 129.9, 129.8, 129.3, 128.4, 125.5, 108.9, 78.5, 78.5, 70.0, 51.6, 38.3, 33.8, 31.9, 30.3, 29.5, 29.4, 28.4, 25.8, 25.7, 22.8, 22.0, 14.2; IR (CHCl_3_) (ν_max_, cm^–1^) 3461, 2910, 1737, 1645, 1381; [α]_D_ = +11.48 (*c* = 0.9 in CHCl_3_); HRMS (ESI) [M + H]^+^ calcd 485.3015 for C_28_H_41_N_2_O_5_; found, 485.3017.

##### Methyl 4-((4*S*,5*R*)-5-((*E*)-2-(3-((*S*)-1-Hydroxyoctyl)quinoxalin-2-yl)vinyl)-2,2-dimethyl-1,3-dioxolan-4-yl)butanoate ((1*S*)-**32**)

*Bis*(benzonitrile)Pd(II)chloride (7 mg, 0.018 mmol, 0.15 equiv) and dppb (13 mg, 0.03 mmol, 0.25 equiv) were dissolved in toluene (1 mL) and stirred at room temperature for 30 min to give a creamy orange solution. Quinoxaline alcohol (1*S*)-**30** (40 mg, 0.119 mmol, 1 equiv), dissolved in toluene (1 mL), and boronic ester **18** (57 mg, 0.161 mmol, 1.35 equiv), dissolved in toluene (1 mL), were added followed by EtOH (0.06 mL) and Na_2_CO_3_ (0.14 mL of a 1 M aq solution, 0.14 mmol, 1.15 equiv). The reaction mixture was heated to 110 °C and stirred for 42 h. The reaction mixture was diluted with H_2_O (10 mL) and extracted with EtOAc (3 × 10 mL), and the extracts were washed with H_2_O (10 mL) and brine (15 mL), dried with MgSO_4_, filtered, and concentrated. The crude product was purified by silica gel column chromatography (10:1 → 3:1 pentane/EtOAc) to give product (1*S*)-**32** as a pale-yellow oil (17 mg, 29%). *R_f_* = 0.19 (6:1 pentane/EtOAc); ^1^H NMR (400 MHz, CDCl_3_) δ 8.09–7.99 (m, 2H), 7.76– 7.68 (m, 2H), 7.17 (dd, *J* = 15.0, 6.0 Hz, 1H), 6.97 (dd, *J* = 15.0, 1.0 Hz, 1H), 5.15 (td, *J* = 7.5, 3.0 Hz, 1H), 4.86 (td, *J* = 6.5, 1.0 Hz, 1H), 4.68 (d, *J* = 7.5 Hz, 1H), 4.32 (dt, *J* = 8.0, 6.5 Hz, 1H), 3.62 (s, 3H), 2.43–2.27 (m, 2H), 1.93–1.82 (m, 2H), 1.78–1.69 (m, 1H), 1.59 (s, 3H), 1.57–1.51 (m, 5H), 1.44 (s, 3H), 1.32–1.24 (m, 8H), 0.86 (t, *J* = 7.0 Hz, 3H); ^13^C NMR (101 MHz, CDCl_3_) δ 173.9, 155.3, 147.0, 142.0, 140.0, 136.0, 129.9, 129.9, 129.3, 128.4, 125.3, 108.9, 78.5, 78.5, 70.0, 51.7, 38.5, 33.8, 32.0, 30.4, 29.7, 29.4, 28.3, 25.9, 25.8, 22.8, 22.0, 14.2; IR (CHCl_3_) (ν_max_, cm^–1^) 3461, 2910, 1737, 1645, 1381; [α]_D_ = −75.51 (*c* = 0.9 in CHCl_3_); HRMS (ESI) [M + H]^+^ calcd 485.3015 for C_28_H_41_N_2_O_5_; found, 485.2994.

##### Methyl (5*S*,6*R*,*E*)-5,6-Dihydroxy-8-(3-((*R*)-1-hydroxyoctyl)quinoxalin-2-yl)oct-7-enoate ((1*R*)-**8**)

Acetonide (1*R*)-**32** (33 mg, 0.068 mmol, 1 equiv) was dissolved in MeOH (1.5 mL), camphorsulfonic acid (13 mg, 0.055 mmol, 0.8 equiv) was added, and the reaction mixture was stirred at room temperature for 2 h. Further, camphorsulfonic acid (13 mg, 0.055 mmol, 0.8 equiv) was added and the reaction mixture was stirred for 1 h. The mixture was concentrated and was purified by preparative thin-layer chromatography (96:4 CH_2_Cl_2_/MeOH). Product (1*R*)-**8** was isolated as a yellow oil (11 mg, 37%). *R_f_* = 0.31 (96:4 CH_2_Cl_2_/MeOH); ^1^H NMR (400 MHz, CDCl_3_) δ 8.06–7.97 (m, 2H), 7.74–7.67 (m, 2H), 7.24 (dd, *J* = 15.0, 5.5 Hz, 1H), 7.02 (dd, *J* = 15.0, 1.5 Hz, 1H), 5.18–5.11 (m, 1H), 4.63 (d, *J* = 7.0 Hz, 1H), 4.51–4.43 (m, 1H), 3.89–3.82 (m, 1H), 3.66 (s, 3H), 2.65 (s, 1H), 2.54 (s, 1H), 2.38 (t, *J* = 7.5 Hz, 2H), 1.95–1.84 (m, 2H), 1.81–1.66 (m, 2H), 1.63–1.52 (m, 4H), 1.29–1.21 (m, 8H), 0.85 (t, *J* = 7.0 Hz, 3H); ^13^C NMR (101 MHz, CDCl_3_) δ 174.1, 155.1, 147.0, 141.7, 139.9, 137.7, 129.8, 129.7, 129.0, 128.3, 125.3, 75.1, 74.0, 69.9, 51.6, 38.0, 33.6, 31.8, 31.4, 29.4, 29.2, 25.4, 22.6, 21.1, 14.1; IR (CHCl_3_) (ν_max_, cm^–1^) 3386, 3020, 2254, 1720, 1216; [α]_D_ = +33.52 (*c* = 0.7 in CHCl_3_); HRMS (ESI) [M + H]^+^ calcd 445.2702 for C_25_H_37_N_2_O_5_; found, 445.2704.

##### Methyl (5*S*,6*R*,*E*)-5,6-Dihydroxy-8-(3-((*S*)-1-hydroxyoctyl)quinoxalin-2-yl)oct-7-enoate ((1*S*)-**8**)

Acetonide (1*S*)-**32** (20 mg, 0.041 mmol, 1 equiv) was dissolved in MeOH (1.5 mL), camphorsulfonic acid (8 mg, 0.034 mmol, 0.8 equiv) was added, and the reaction mixture was stirred at room temperature for 3.5 h. The mixture was concentrated and was purified by preparative thin-layer chromatography (96:4 CH_2_Cl_2_/MeOH). Product (1*S*)-**8** was isolated as a yellow oil (7 mg, 39%). *R_f_* = 0.31 (96:4 CH_2_Cl_2_/MeOH); ^1^H NMR (400 MHz, CDCl_3_) δ 8.08–7.98 (m, 2H), 7.75–7.68 (m, 2H), 7.22 (dd, *J* = 15.0, 6.5 Hz, 1H), 7.03 (dd, *J* = 15.0, 1.5 Hz, 1H), 5.19–5.14 (m, 1H), 4.66 (d, *J* = 6.5 Hz, 1H), 4.54–4.45 (m, 1H), 3.91–3.80 (m, 1H), 3.66 (s, 3H), 2.58 (s, 1H), 2.47 (s, 1H), 2.37 (td, *J* = 7.0, 1.5 Hz, 2H), 1.94–1.84 (m, 2H), 1.81–1.65 (m, 2H), 1.61–1.51 (m, 4H), 1.31–1.23 (m, 8H), 0.86 (t, *J* = 7.0 Hz, 3H); ^13^C NMR (101 MHz, CDCl_3_) δ 174.2, 155.2, 147.1, 141.7, 139.9, 137.7, 129.8, 129.7, 129.0, 128.3, 125.2, 75.1, 73.9, 70.0, 51.6, 38.0, 33.6, 31.8, 31.3, 29.4, 29.2, 25.5, 22.6, 21.0, 14.1; IR (CHCl_3_) (ν_max_, cm^–1^) 3386, 3020, 2254, 1720, 1216; [α]_D_ = −69.52 (*c* = 0.7 in CHCl_3_); HRMS (ESI) [M + H]^+^ calcd 445.2702 for C_25_H_37_N_2_O_5_; found, 445.2722.

### Biology Materials and Methods

#### Materials

##### *In Vitro* Study

THP-1 monocytes were purchased from LGC Promochem (Middlesex, U.K.). The Vybrant Phagocytosis Assay kit (V-6694) was obtained from Molecular Probes. THP-1-Lucia nuclear factor (NF)-κB cells, heat-killed Listeria monocytogenes (HKLM), Zeocin, Normocin, QUANTI-Luc, Geneticin (G418), and Hygromycin were purchased from Invivogen (CA); the Pierce BCA Protein Assay kit was from Thermo Scientific (Ireland); the Human Pro-Inflammatory multi-Plex Tissue Culture kit was from MSD (Maryland); and the cytotoxicity detection kit was from Roche (Germany). Transfecting SMCs with an NF-κB reporter plasmid pNF-κB-SEAP vector were purchased from Takara/Clontech (Ireland). HEK-293-transfected and wild-type cell lines were kindly provided by Actelion Pharmaceuticals Ltd. (Switzerland). For calcium measurement, sterile plates black-sided with optically clear glass flat bottoms were purchased from Perkin Elmer (U.K.). LXA_4_**1** was obtained from Calbiochem (U.K.), and dexamethasone was from Sigma-Aldrich (Ireland).

##### *In Vivo* Studies

C57BL/6J mice were purchased from Charles River (U.K.). Zymosan was obtained from Sigma-Aldrich (Ireland) and used in both *in vivo* studies. Flow cytometry mAb’s were obtained from BD Biosciences or eBioscience.

### Methods

#### Study Design

##### *In Vitro* Screening

In this study, we initially screened the quinoxaline-containing sLXms (QNX-sLXms) for safety and anti-inflammatory bioactions using a THP-1 monocyte cell line. The eight candidate molecules screened were divided into four groups, based on the synthetic strategy adopted (Figure 2).

- **Group A**: (*R*)- and (*S*)-epimers of the QNX derivatives display an acetonide on the upper chain and maintain the lower chain of the same length as the LXA_4_ native compound (six-carbon chain, -**6**C-). We refer to them as (*R*)-**17** and (*S*)-**17**.
- **Group B**: (*R*)- and (*S*)-epimers of the QNX derivatives display a diol on the upper chain and maintain the lower chain of the same length as LXA_4_ (six-carbon chain, -**6**C-). We refer to them as (*R*)-**6** and (*S*)-**6**.
- **Group C**: (*R*)- and (*S*)-epimers of the QNX derivatives display a diol on the upper chain and shorten the lower chain of two carbons compared to the native compound (four-carbon chain, -**4**C-). We refer to them as (*R*)-**7** and (*S*)-**7**.
- **Group D**: (*R*)- and (*S*)-epimers of the QNX derivatives display a diol on the upper chain and extend the lower chain of two extra carbons compared to the native compound (eight-carbon chain, -**8**C-). We refer to them as (*R*)-**8** and (*S*)-**8**.

#### Cell Culture

##### THP-1-Lucia Human Monocytes

Mycoplasma-free THP-1-Lucia NF-κB cells were grown in an RPMI 1640 (Gibco) medium containing 2 mM l-glutamine, 1.50 g/L sodium bicarbonate, 4.50 g/L glucose, 10 mM *N*-(2-hydroxyethyl)piperazine-*N*′-ethanesulfonic acid (HEPES), and 1 mM sodium pyruvate. Then, 10% heat-inactivated fetal bovine serum (FBS) (30 min at 56 °C), 100 μg/mL Normocin, and Pen-Strep (50 U/mL to 50 μg/mL) were also added to the growth media. As THP-1-Lucia cells were resistant to Zeocin, the latter was used as a selective antibiotic (a high dose, i.e., 100 μg/mL, was alternated every 2 weeks with a low dose, i.e., 10 μg/mL).

LXA_4_**1** and all of the tested sLXms were dissolved in ethanol and further diluted in a culture medium (final ethanol concentration 0.1%; equivalent concentrations were used as the vehicle control). Prior to *in vitro* experiments, THP-1-Lucia NF-κB cells were harvested and plated at 1 × 10^5^ cells/well on 96-well plates and left to settle in the incubator at 37 °C, 95% humidity, and 5% CO_2_, in 0.1% FBS-containing media, pretreated for 30 min with increasing doses of LXA_4_**1**, sLXms (from 10^–12^ to 10^–**6**^ M), or appropriate controls, followed by stimulation with 50 ng/mL LPS for 24 h. Untreated (Unt) or vehicle-treated (Veh) cells were used as negative controls; 1 pM (1*R*)-**5**, the (1*R*)-epimer of the benzo-mimetic, was used as a positive control for any aromatic mimetic; and, finally, we compared the observed effects to the conventional glucocorticoid dexamethasone (Dex) treatment, at 1 μM.

Notably, the same conditioned media was used for measuring NF-κB activity, cytotoxicity, and cytokine levels, strengthening the power of our findings.

##### THP-1 Human Monocytes and Derived Macrophages

Mycoplasma-free THP-1 monocytes were grown in an RPMI 1640 (Gibco) medium containing 2 mM l-glutamine, 2.00 g/L sodium bicarbonate, and 2.00 g/L glucose. Then, 10% (v/v) heat-inactivated FBS (30 min at 56 °C) and Pen-Strep (100 U/mL to 100 μg/mL) were supplemented to the growth media.

Monocytes were used for the relevant assays or, alternatively, were differentiated to an MF0 macrophage over 4 days, using 16 nM phorbol 12-myristate 13-acetate [PMA; dissolved in 0.02% dimethyl sulfoxide (DMSO)] for 48 h, followed by 24 h of resting, prior to replacement of media containing 0.1% FBS and subsequent treatment with LXA_4_, sLXms, or appropriate controls (see details ahead).

##### Mouse Primary Vascular Smooth Muscle Cells (SMCs)

Mouse primary vascular SMCs were cultured at 37 °C in a humidified atmosphere of 95% air/5% CO_2_ and maintained in Dulbecco’s modified Eagle’s medium (DMEM; Life Technologies) supplemented with 25 mmol/L glucose with 10% (v/v) FBS. For treatments, media contained only 1% FBS. After serum restriction for 24 h, cells were stimulated with LXA_4_, sLXms, or appropriate controls (see details ahead).

##### HEK-293 Human Embryonic Kidney

HEK-293-transfected and wt cell lines were cultured in Dulbecco’s modified Eagle’s medium (DMEM Glutamax, 4 mM l-glu and 4.50 g/L glucose—Gibco, Ireland) supplemented with heat-inactivated (30 min at 56C) 10% (v/v) FBS (Invitrogen, U.K.), 50 U penicillin, and 50 μg of streptomycin (Invitrogen, U.K.). As stably double-transfected FPR2^+^/Gα_q_^+^ HEK-293 cells were resistant to Geneticin and Hygromycin, these were used as selective antibiotics (500 and 100 μg/mL, respectively) to maintain the stable double transfection with the ALX/FPR2 receptor and the Gα_q_ subunit coupled to it. Immediately prior to calcium measurement, fluorescently labeled cells were treated with 10^–11/–9/–7^M LXA_4_**1** or sLXms using an injection system coupled to the fluorescence reader.

#### Cell Treatment

For *in vitro* screening and *in vivo* testing, depending on the specific assay requirements, the following treatments were used.

- *Vehicle*. 0.1% Ethanol (EtOH) was used as the vehicle control. Highly concentrated LXA_4_ and sLXms were dissolved in pure EtOH to make stocks and then were added to cells at the required working concentration, keeping EtOH to a safe concentration of 0.1%.
- *ALX/FPR2 agonists*. Depending on the specific assay, the native compound LXA_4_ (within 1 pM–1 μM range) or W-peptide (2 nM) was used as the control for, respectively, endogenous or exogenous ALX/FPR2 receptor agonism.
- *TLR-dependent/-independent NF-κB pathway inducers*. Depending on the specific assay, a series of inflammatory stimuli were adopted to challenge *in vitro* monocytes or SMCs over 24 h. Lipopolysaccharide (LPS) (50 ng/mL), an endotoxin expressed on the outer cell membrane of Gram-negative bacteria, was used to mimic a TLR-dependent infective/inflammatory stimulus.^70^ Tumor necrosis factor α (TNF-α) (1 ng/mL) was used to mimic a TLR-independent and noninfective inflammatory stimulus.^71^ Heat-killed *Listeria monocytogenes* (HKLM) (10^8^ cells/mL) was selected as a positive control for TLR2 activity and downstream NF-κB activation, in an infective scenario.^72^ To mimic *in vivo* TLR2-dependent NF-κB-driven acute and local inflammation, mice were challenged with zymosan (1 mg/mouse) to induce peritonitis^73^ or with carraggenan (50 μg/g) to induce paw edema.^74^
- *“Classical” anti-inflammatory drugs*. Dexametasone (Dex) (1 μM–1 μg/g) is a glucocorticoid used here in an *in vitro*/*in vivo* control for anti-inflammatory activity.^62^ Naproxen is a classical NSAID used here as an *in vivo* control for anti-inflammatory activity.^75^
- *Aromatic Lipoxin A*_*4*_*-mimetic*. Benzo-lipoxin [**2**] (1 pM) is the first LXA_4_ analogue asymmetrically synthesized^34^ and is here used as a control for any aromatic sLXm activity.

#### NFκB-Driven Luciferase Gene Reporter Assay in THP-1 Lucia Monocytes

NFκB activity was monitored in the human THP-1 monocytic leukemia cell line containing a stably integrated NF-κB-inducible Lucia reporter construct (as previously reported).^21^ When stimulated with LPS, the transcription factor NF-κB is activated, upregulating the synthesis of proinflammatory mediators, thus bringing about an inflammatory response. In these cells, NF-κB activation is linked to a firefly luciferase gene and can be measured using luminescence.

Prior to *in vitro* experiments, THP-1-Lucia NF-κB cells were pretreated for 30 min with increasing doses of native LXA_4_, quinoxaline analogues (from 10^–12^ to 10^–6^ M), or appropriate controls, followed by stimulation with LPS and incubation for 24 h. Heat-killed listeria monocytogenes (HKLM) (10^8^/mL) was used as a positive control.^28^ The levels of NF-κB-induced Luciferase were then measured in a culture supernatant using QUANTI-Luc as the detection reagent, and measurements were taken on a bioluminescence reader. The results are reported as a % relative to LPS treatment, representing 100% luciferase activity.

A total of 1 × 10^5^ cells/well were treated in a 96-well plate as mentioned above. After 24 h, the levels of NF-κB-induced Lucia were assayed in a cell culture supernatant using QUANTI-Luc, as a detection reagent, on a bioluminescence reader (Synergy HT, BioTek Instruments, Inc. Vermont). Aliquots of the supernatant were retained for subsequent cytokine and LDH assays. Cellular proteins were extracted with a radioimmunoprecipitation assay (RIPA) buffer (Thermo Fisher, Ireland), supplemented with protease inhibitors (Sigma-Aldrich, Ireland). Lysates were clarified by centrifugation (3200*g*, 10’, 4 °C) to remove cell debris, and the cell protein level was quantified by performing a bicinchoninic acid (BCA) assay (as per the manufacturer’s instructions) reading the protein absorbance at 570 nm, using a spectrophotometer (SPECTRAMAX M2, Molecular Devices (U.K.) Limited). A “relative luminescence unit” (RLU) was obtained using the average value of the lum/abs ratio of two replicates.

#### Multiplex Electrochemiluminescence Detection for Cytokine Quantification

After 24 h of exposure to LXA_4_**1**, sLXms, or vehicle, followed by LPS stimulation (50 ng/mL), as described above, THP-1-Lucia NF-κB-cell supernatants were collected and cleared by centrifugation. Concentrations of IFN-γ, IL-1β, IL-6, IL-8, IL-10, IL-12p70, and TNF-α in culture supernatants were assessed by electro-chemi-luminescent detection, using a commercially available human 96-well plate “multiplex” (several analytes in the same well) kit for tissue culture samples, according to the manufacturers’ guidelines.

#### Cytotoxicity Assay

Lactate dehydrogenase (LDH) was assayed in cell supernatants to investigate possible cytotoxicity of the lead compound. LDH is rapidly released into the culture medium after disruption of the plasma membrane.^67,69^ After 24 h of exposure to sLXms, vehicle, or controls, followed by LPS stimulation, as described above, THP-1-Lucia NF-κB-cell supernatants were collected and LDH was assayed. Cytotoxicity was evaluated relative to vehicle controls. Maximum releasable LDH was determined in cells lysed with 2% Triton-X-100 (Sigma-Aldrich, Ireland). LDH levels were assessed by reading the absorbance at 490 nm (protein) and 690 nm (background noise), using a spectrophotometer (SPECTRAMAX M2, Molecular Devices (U.K.) Limited).

#### NFκB-Driven Luciferase Gene Reporter Assay on vSMCs

NF-κB activity was assessed (as previously reported)^18^ by transfecting SMCs with an NF-κB reporter plasmid (pNF-κB-SEAP vector; Takara/Clontech) for 24 h and subsequently stimulating SMCs with TNF-α (1 ng/mL; R&D Systems) for 24 h in the presence or absence of the vehicle (0.1% ethanol), LXA_4_ (0.1 nmol/L; Calbiochem), or (*R*)-**6** (1 nmol/L). NF-κB activity was determined by measuring secreted alkaline phosphatase in the culture supernatant using the SEAP Reporter Gene Assay System (Roche Australia). Cell experiments were performed six to eight times, and the values presented are the mean ± SEM from independent experiments.

#### Phagocytosis Assay

After differentiation of THP-1 monocytes (Mo) to macrophage (MF0) (48 h of PMA-trigger followed by 24 h of resting), on day 4, 1 × 10^5^ cells/well in 100 μL of 0.1% fetal calf serum (FCS) containing RPMI media Mo or MF0 were plated (data not shown). Cell adherence was allowed for 1 h and 30 min at 37 °C/5% CO_2_. Cells were then treated for 30 min with controls and phagocytosis effectors for testing (vehicle, LXs, Rvs, or sLXms). Subsequently, media containing controls/effectors were vacuum-aspirated and replaced with a solution containing fluorescently prelabeled *E. coli*-derived bioparticles and cells were incubated in the dark for 2 h at 37 °C/5% CO_2_ to allow particle ingestion by monocytes/macrophages. After that, media containing bioparticles were vacuum-aspirated and replaced with a solution containing trypan blue and cells were incubated for 1 min at room temperature to allow quenching of unbound particles to ensure specificity of the fluorescent signal, deriving only from ingested particles (not shown). After aspiration of Trypan, the fluorescence reading was immediately performed at the following conditions: end point; reading from the bottom of the plate; Ex.480/Em.520 nm.

#### *In Vivo* Peritonitis Model

Male C57BL/6J mice of 9 weeks of age were purchased from Charles River (Kent, U.K.) and were housed in a specific pathogen-free facility in individually ventilated and filtered cages under positive pressure. Peritonitis was induced in mice by ip injection of zymosan (purchased from Sigma-Aldrich; 1 mg per mouse in 0.5 mL of phosphate-buffered saline (PBS)).^73^ Mice were treated with dexamethasone (1 μg/g) purchased from Sigma-Aldrich 1 h prior to zymosan injection or (*R*)**-6** (2 and 6 ng/g) (200 μL IP) 30 min prior to zymosan injection. Peritoneal cells were collected by lavage 4 h after injection of zymosan. The local Animal Use and Care Committee and The UK Home Office approved the animal experiments in accordance with the derivatives of both The Home Office Guidance on the Operation of Animals (Scientific Procedures) Act 1986 and The Guide for the Care and Use of Laboratory Animals of the National Research Council.

#### Flow Cytometry

Surface marker expression on peritoneal cells was assessed by flow cytometry with data collection on a FACSCalibur (Becton Dickinson). Data were analyzed with FlowJo analysis software (Treestar Inc.). Cells were stained with AlexaFluor488 conjugated anti-F4/80 (clone BM8; eBioscience); APC conjugated anti-CD11b (clone M1/70; eBioscience) and Pacific blue conjugated anti-Ly6G (clone 1A8; BioLegend) antibodies. Prior to surface staining, peritoneal cells were counted manually (Neubauer chamber) with Turks solution. Using appropriate fluorescence minus one (FMO) controls, quadrants were drawn, and data were plotted on logarithmic-scale density- or dot-plots.

NB:

F4/80: murine macrophage marker

Siglec F: eosinophil marker

Ly6G: neutrophil PMN marker

CD11b: granulocytes, monocytes/macrophages, dendritic cells, NK cells, and subsets of T- and B-cell (myeloid cells) marker

AF488: Alexa Fluor 488 (fluorochrome)

APC: Allophycocyanin (fluorochrome)

#### *In Vivo* Paw Edema Model

The most and the least potent and efficient QNXs tested in this study (respectively, (*R*)-**6** and (*R*)-**17**, 45 ng/mouse = 2 μg/kg, ip) or Naproxen (50 mg/kg, po) were administered 30 min before the intrapaw injection of 1% carrageenan into male C57bl/6 mice.^57,74^ Then, 50 μL of 1% carrageenan was injected with an equivalent volume of saline injected into the contralateral paw. Inflammation was presented as the difference in paw thickness over time using gauge (POCO 2T; Kroeplin, GmbH, Surrey, U.K.). Data are presented as mean ± SEM, *n* = 3 mice/treatment group.

#### Intracellular Calcium Flux Assay

To explore the mechanism of action through which the sLXm lead compound exerts the effects seen here, its interaction with the ALX/FPR2 receptor was investigated and agonist-induced intracellular calcium transients were measured using an engineered cell line stably overexpressing the ALX/FPR2 receptor and the Gα_q_ subunit coupled to it (Figure S6). Wild-type HEK cells were used as controls, to verify the specificity of the agonism toward ALX/FPR2, as previously reported.^36^ Briefly, prior to cell plating, sterile 96-well plates (black-sided with optically clear glass flat bottoms) were PDL-coated overnight. Subsequently, 2 × 10^5^ wild-type cells and overexpressed cells/well were cultured for 18 h in DMEM complete media (containing Ca^2+^ and Mg^2+^) to facilitate adherence prior to labeling with Fluo-4 dye. After 1 h of incubation at 37 °C with Fluo-4, cells were gently washed twice and placed in 100% Hanks’ balanced salt solution (HBSS; 37 °C) before reading the fluorescence (485/535 nm), using a spectrophotometer conjugated with an injection system (Clariostar BMG LABTECH plate reader), allowing addition to the cells of controls (1 mM ATP, a GPCR purinergic agonist, 2 nM Wp, a synthetic ALX/FPR2 agonist) or experimental treatments [10 pM, 1 nM, and 100 nM LXA_4_**1** or (*R*)-**6**].

During calcium flux measurement, the kinetic steps involved were the following: the baseline fluorescent signal was measured for 20 s, followed by 100 s immediately after agonist injection. Subsequently, 2% Triton-X-100 was added to lyse the cells (monitoring for 20 s the maximal release of calcium ions in the extracellular environment) and, finally, 25 mM tetraacetic acid (EGTA) was added to chelate calcium ions (monitoring for additional 20 s the minimal detection of calcium ions in the extracellular environment) (Figure S7)**.**

#### Statistical Analysis

For the human monocytic cell line, results were expressed as mean ± SEM relative to the vehicle control. Experimental points were performed in duplicate with a minimum of three independent experiments. Statistical comparisons between controls vs treated groups were made by parametric Student’s unpaired *t*-test with a two-tailed distribution, assuming equal or unequal variance (based on the outcome of the “*F* test two sample for variances”).

Data were analyzed using the Prism 8.4.2 software program for Windows (GraphPad software, San Diego, CA). Differences between two groups were analyzed by two-tailed unpaired Student’s *t*-test and by one-way ANOVA to assess differences between more than two groups using a post-test, Holm–Sidak’s multiple-comparison post hoc test. A *p* < 0.05 was considered significant. For the murine model, statistical analysis was performed using one-way ANOVA with the Newman–Keuls multiple-comparison test. For both analyses, a value of *p* ≤ 0.05 was considered significant.

#### PD Score

For the *in vitro* assays, a PD analysis was conducted for the tested molecules to determine the PD profile *per se* and relative to LXA_4_ (**1**). The “coding” of the heat-map indicates the arbitrary criteria to assign points to each single PD component (efficacy = *I*_max_ or *E*_max_; potency = IC_50_ or EC_50_; slope = HS) to generate a final (aggregate) relative PD score. If a single PD component was greater than the reference level (*x* > 1), then the single-component relative score was arbitrarily set to +1 (light green) or +2 (dark green). If a single PD component was equal to the reference level (*x* = 1), then the single-component relative score was arbitrarily set to 0 (gray). If a single PD component was smaller than the reference level (*x* < 1), then the single-component relative score was arbitrarily set to −1 (light red) or −2 (dark red). If the curve of a single PD component was best fitting to a flat line (*x* = 0), then the single-component relative score was arbitrarily set to −4 (purple) (Table S1).

## Supporting Information Available

PD analysis coding (Table S1); PD analysis of the effects of QNX-sLXms on the NF-κB activity (Table S2); PD analysis of the effects of QNX-sLXms on cytokine release (Table S3); safety index of QNX-sLXms (Table S4); PD analysis of the effects of (*R*)-**6** on macrophage phagocytosis (Table S5); effect of sLXm (*R*)-**6** on murine carrageenan-induced paw edema (Table S6); PD analysis of the effects on intracellular calcium mobilization of (*R*)-**6** QNX-sLXm lead (Table S7); effect of series (**17**), (**7**), and (**8**) of QNX-sLXms on LPS-induced NF-κB-driven luciferase activity in monocytes (Figure S1); effect of series (**7**) and (**8**) of QNX-sLXms on LPS-induced proinflammatory cytokine release in monocytes (Figure S2); effect of QNX-sLXm lead compound (*R*)-**6** on the whole panel of proinflammatory cytokine release in monocytes (Figure S3); intrinsic cytotoxic profile of series (**17**), (**7**), and (**8**) of QNX-sLXms (Figure S4); extrinsic cytotoxic profile of series (**17**), (**7**), and (**8**) of QNX-sLXms (Figure S5); cell model for intracellular calcium flux measurement (Figure S6); intracellular calcium flux kinetic traces (Figure S7) (PDF)
NMR spectra and chromatograms (CSV)

## Notes

Research involving animals was performed in accordance with institutional guidelines as defined by national regulatory authorities.

## Abbreviations

ALX/FPR2: formyl peptide receptor 2
AT-01-KG: attenuate therapeutics-01-KG522
AT-02-CT: attenuate therapeutics-02-CT443
Dex: dexamethasone
HKLM: heat-killed *Listeria monocytogenes*
HS: Hill–Slope
LDH: lactate dehydrogenase
*I*_max_/*E*_max_: maximal inhibitory/excitatory response
IC50/EC50/TC50: half-maximal inhibitory/excitatory/toxic concentration
LPS: lipopolysaccharide
LX: lipoxygenase interaction product (Lipoxin)
NF-κB: nuclear factor-kappa-light-chain-enhancer of activated B cells
PD: pharmacodynamic
PMN: polymorphonuclear cell
QNX-sLXm: quinoxaline-containing synthetic-LX-mimetic
SAR: structure–activity relationship
Si: safety index
SPM: specialized proresolving mediator
TNF-α: tumor necrosis factor-α
TM50: half-maximal time for resolution
Wp: W-peptide
vSMC: vascular smooth muscle cell
