# Supplementary material for: Asymmetric Synthesis and Biological Screening of Quinoxaline-Containing Synthetic Lipoxin A4 Mimetics (QNX-sLXms)
Source: J Med Chem. 2021 Jun 17;64(13):9193–216. doi: 10.1021/acs.jmedchem.1c00403 (PMC8279484; doi:10.1021/acs.jmedchem.1c00403)
Supplement: Supplementary file 1 — jm1c00403_si_001.pdf [file jm1c00403_si_001.pdf]

Supporting Information

Asymmetric synthesis and biological screening of quinoxaline-containing synthetic lipoxin A_4 mimetics (QNX-sLXms)

Monica de Gaetano[a, ‡], Catherine Tighe[b, ‡], Kevin Gahan[b], Andrea Zanetti[b], Jianmin Chen[c], Justine Newson[d], Antonino Cacace[a], Mariam Marai[a], Andrew Gaffney[a], Eoin Brennan[a], Phillip Kantharidis[e], Mark E. Cooper[e], Xavier Leroy[f], Mauro Perretti[c], Derek Gilroy[d], Catherine Godson[*a] and Patrick J. Guiry[*, b].

[a] School of Medicine, Diabetes Complications Research Centre, UCD Conway Institute, University College Dublin, Belfield, Dublin D04 N2E5, Ireland.

[b] Centre for Synthesis and Chemical Biology, School of Chemistry, UCD Conway Institute, University College Dublin, Belfield, Dublin D04 N2E5, Ireland.

[c] William Harvey Research Institute, Queen Mary University London, London, EC1M 6BQ, UK.

[d] Centre for Clinical Pharmacology, UCL, London, WC1E 6JF, UK.

[e] Department of Diabetes, Central Clinical School, Monash University, Melbourne, VIC 3004, Australia.

[f] Domain Therapeutics SA, 67400 Strasbourg - Illkirch - France.

‡ *These authors contributed equally to this body of work.*

* *Corresponding authors: p.guiry@ucd.ie; catherine.godson@ucd.ie*

Table of Contents

^{1}H and ^{13}C NMR Spectra Pages 3-20

Chromatograms Pages 21-29

Supplementary Tables (ST) and Figures (SF) List Page 30

ST1: PD Analysis Coding Page 31
ST2: PD Analysis of the Effects of QNX-sLXms on NF-κB Activity Page 32
ST3: PD Analysis of the Effects of QNX-sLXms on Cytokine Release Pages 33-34
ST4: Safety Index of QNX-sLXms Page 35
ST5: PD Analysis of the Effects of (*R*)-**6** on Macrophage Phagocytosis Page 36
ST6: Effect of QNX-sLXm (*R*)-**6** on Murine Carregeenan-induced Phagocytosis Page 37
ST7: PD Analysis of the Effects on Intracellular Ca Mobilization of (*R*)-**6** Page 38
ST6: Effect of QNX-sLXm (*R*)-**6** on Murine Carregeenan-induced Phagocytosis Page 37

SF1: Effect of Series (**17**), (**7**), (**8**) of of QNX-sLXms on LPS-induced NF-κB Driven Luciferase Actvity in Monocytes Pages 39-40
SF2: Effect of Series (**6**) of of QNX-sLXms on LPS-induced Pro-inflammatory Cytokine Release in Monocytes Pages 41-42
SF3: Effect of Series (**7**) and (**8**) of of QNX-sLXms on LPS-induced NF-κB Driven Luciferase Actvity in Monocytes Pages 43-44
SF4: Intrinsic Cytotoxic Profile of Series (**17**), (**7**), (**8**) of of QNX-sLXms Pages 45-46
SF5: Extrinsic Cytotoxic Profile of Series (**17**), (**7**), (**8**) of of QNX-sLXms Pages 47-48
SF6: Cell Model for Intracellular Calcium Flux Measurement Pages 49-50
SF7: Intracellular Calcium Flux Kinetic Traces Pages 51-53

^{1}H and ^{13}C NMR Spectra

^{1}H NMR (400 MHz, $CDCl_3$)

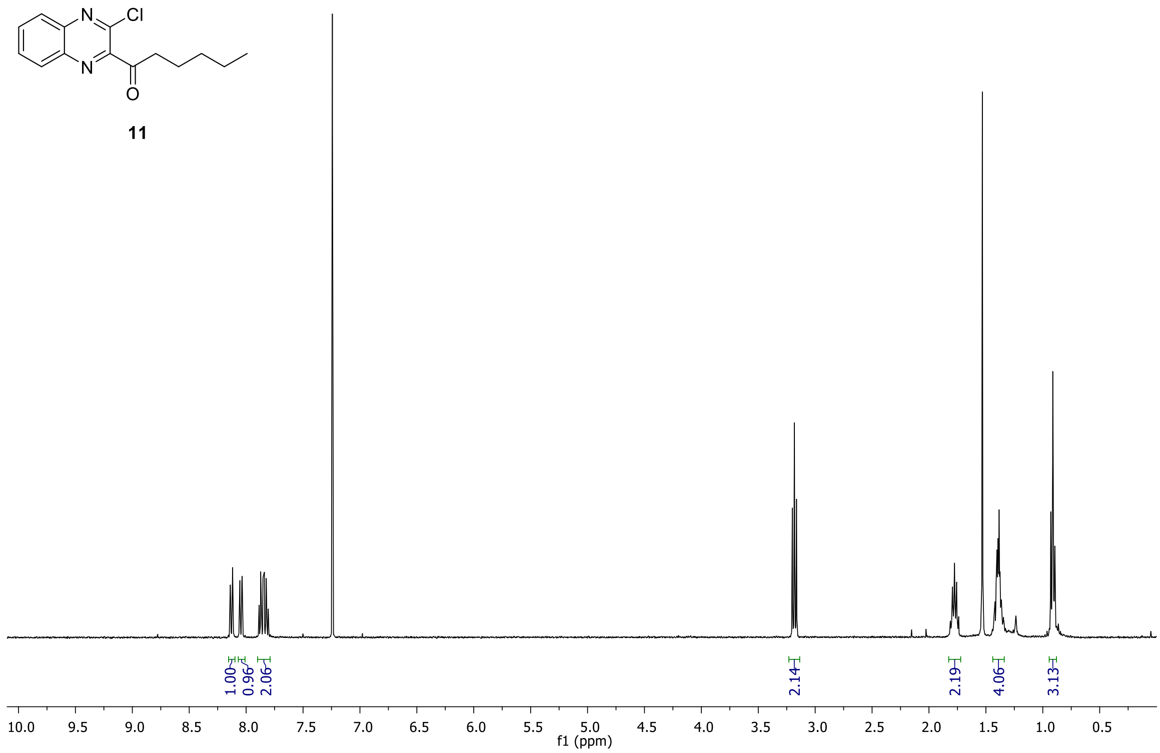

^{13}C NMR (101 MHz, $CDCl_3$)

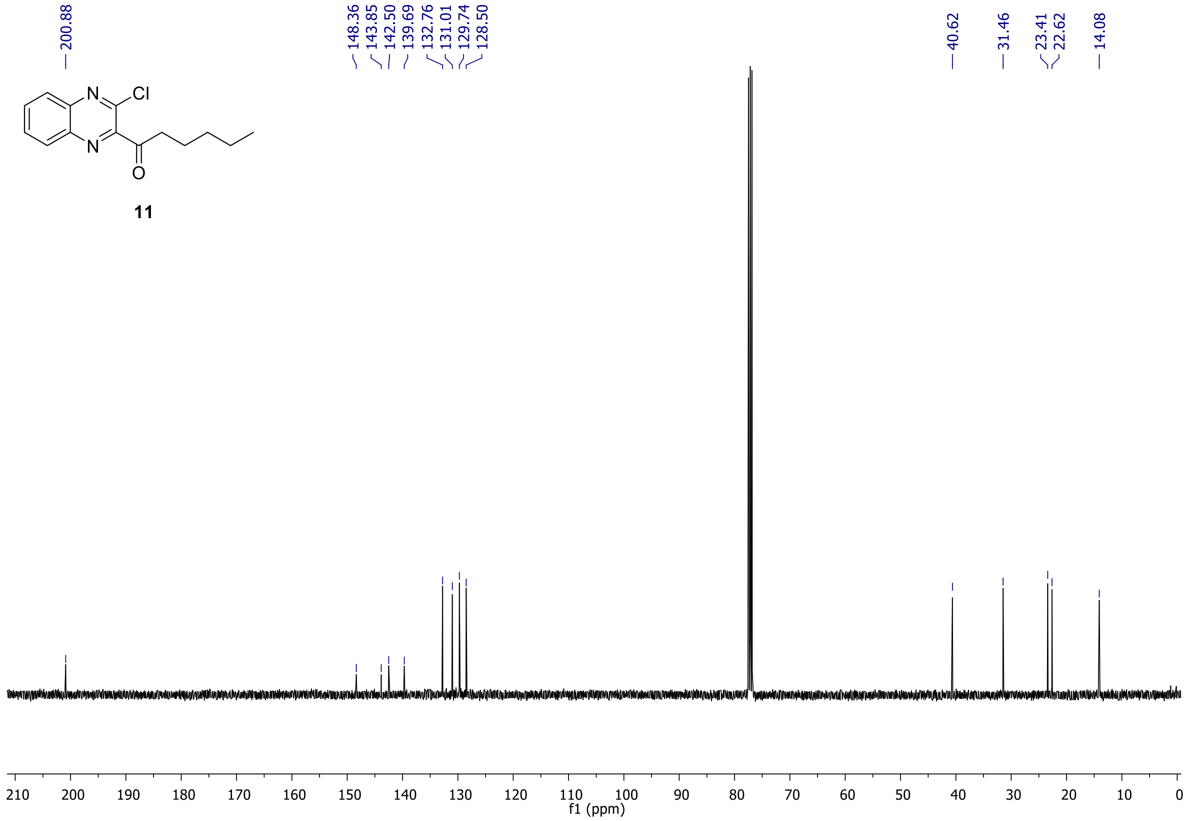

^{1}H NMR (400 MHz, $CDCl_3$)

14

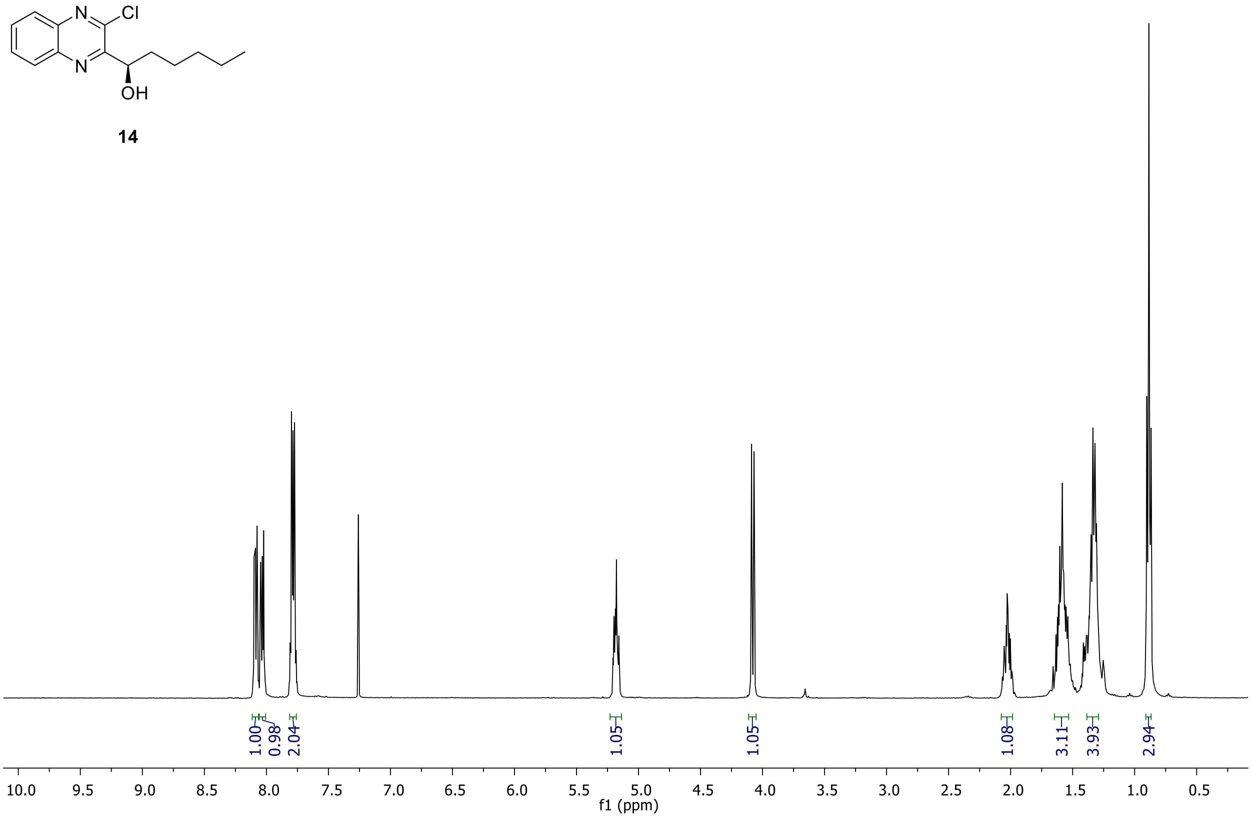

^{13}C NMR (101 MHz, $CDCl_3$)

14

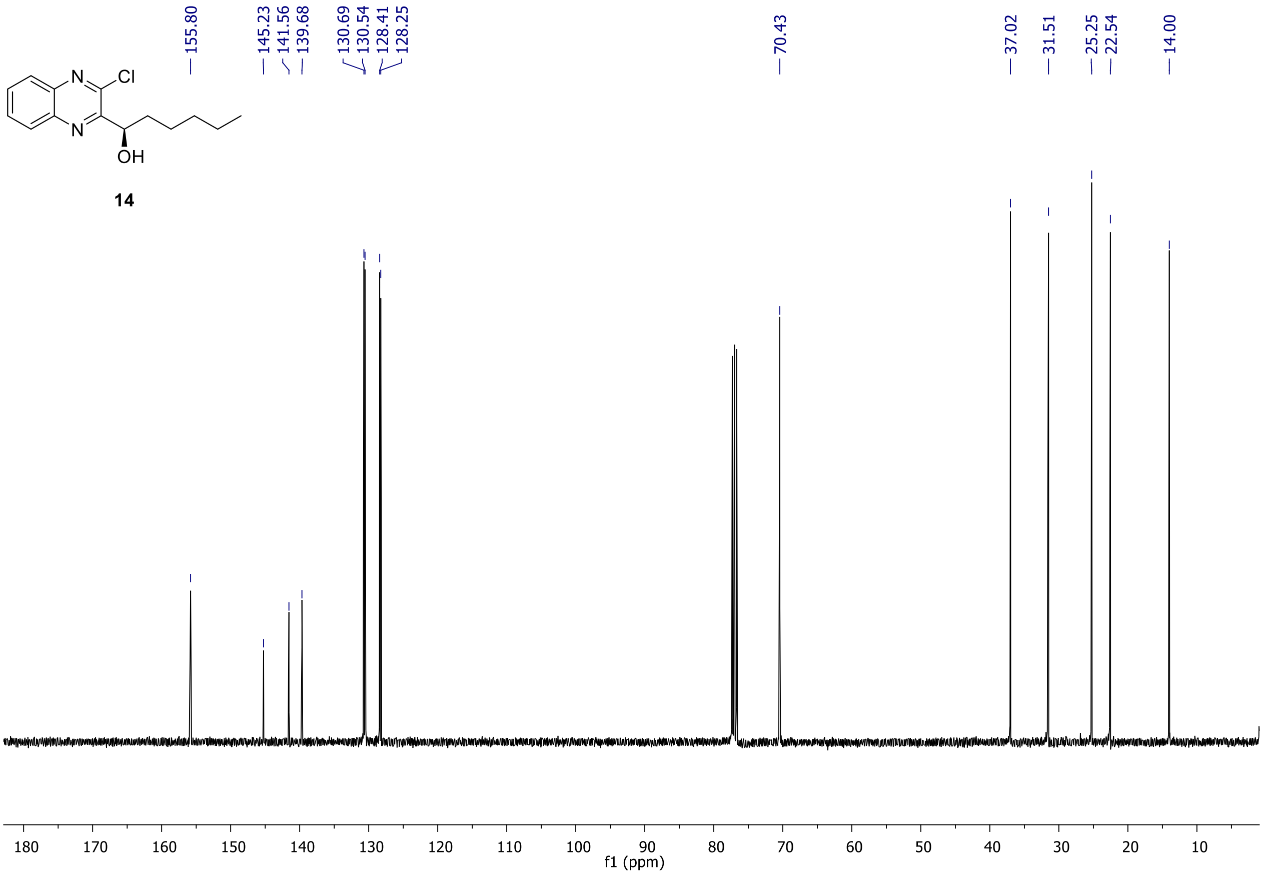

^{1}H NMR (400 MHz, $CDCl_3$)

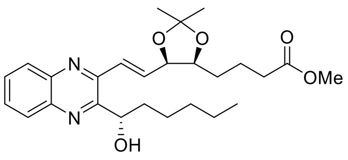

(1*S*)-**17**

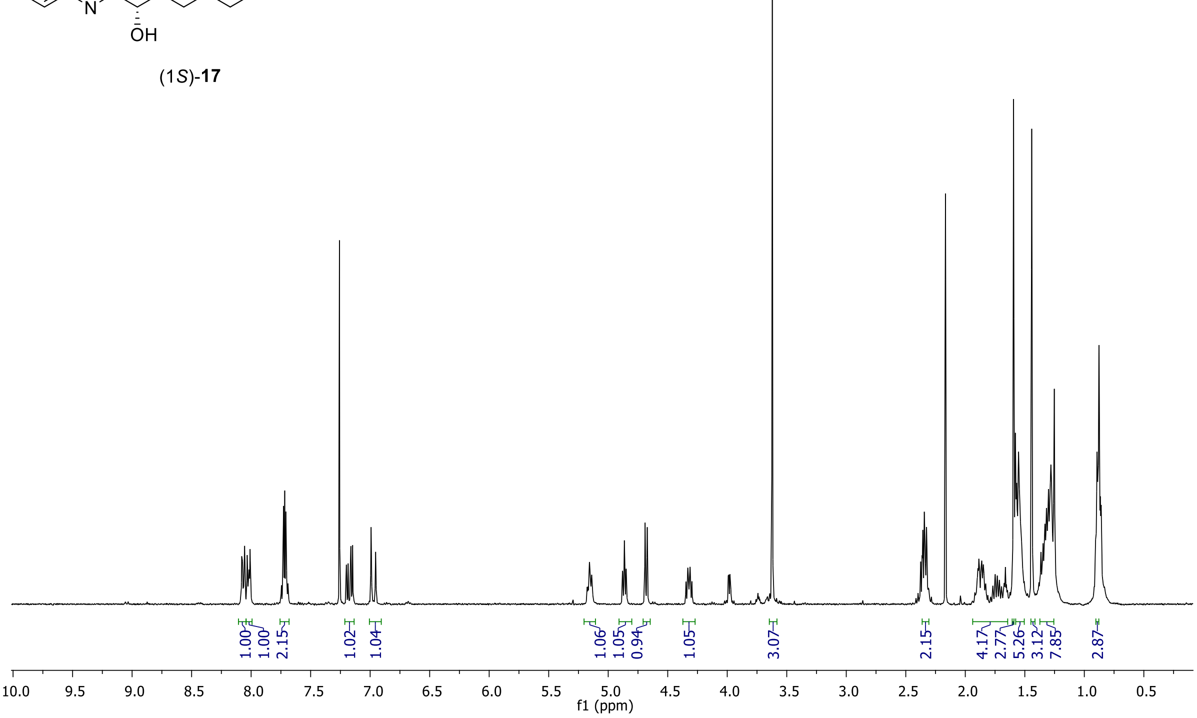

^{13}C NMR (101 MHz, $CDCl_3$)

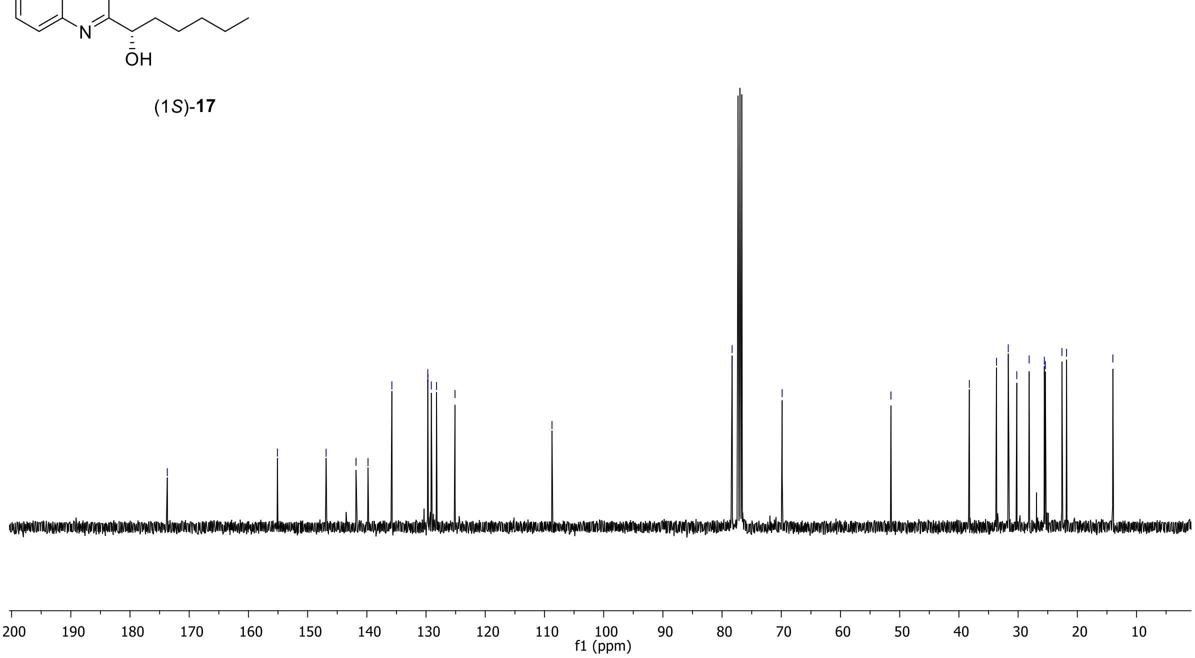

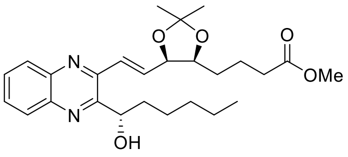

(1*S*)-**17**

^{1}H NMR (400 MHz, $CDCl_3$)

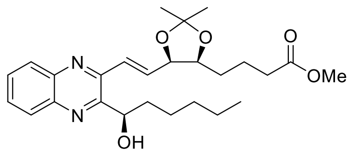

(1*R*)-**17**

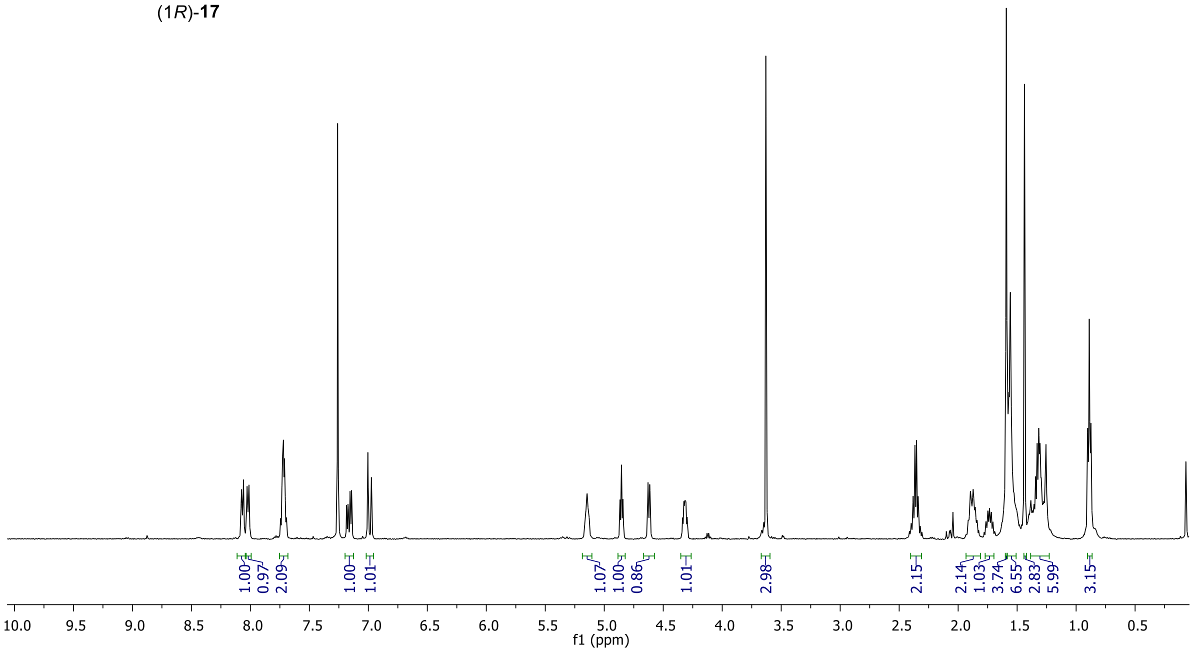

^{13}C NMR (101 MHz, $CDCl_3$)

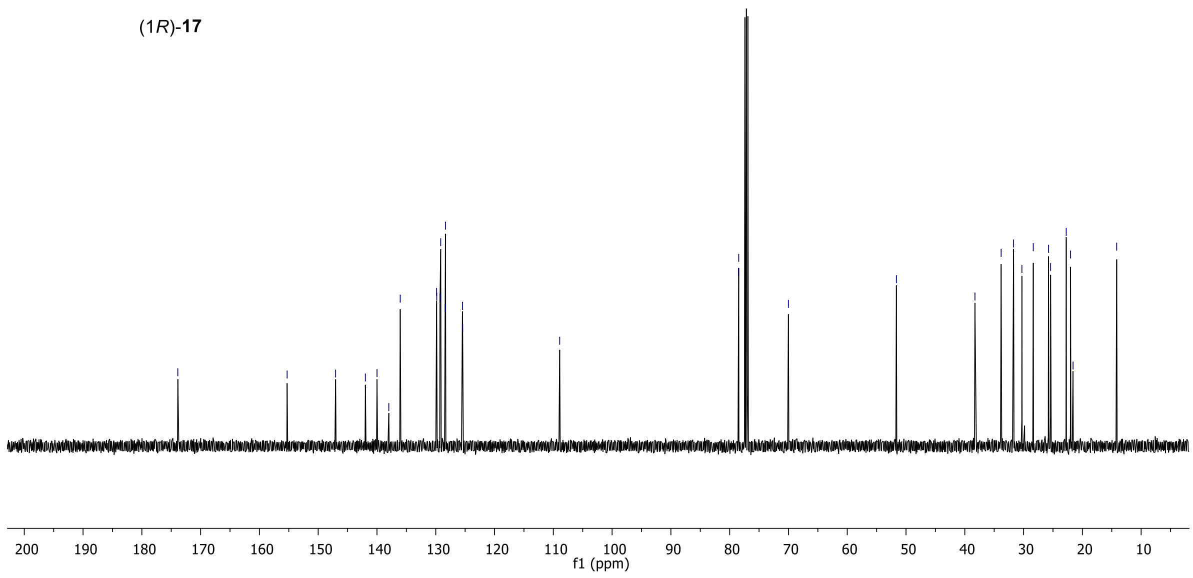

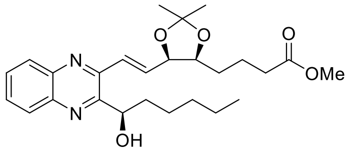

(1*R*)-**17**

^{1}H NMR (400 MHz, $CDCl_3$)

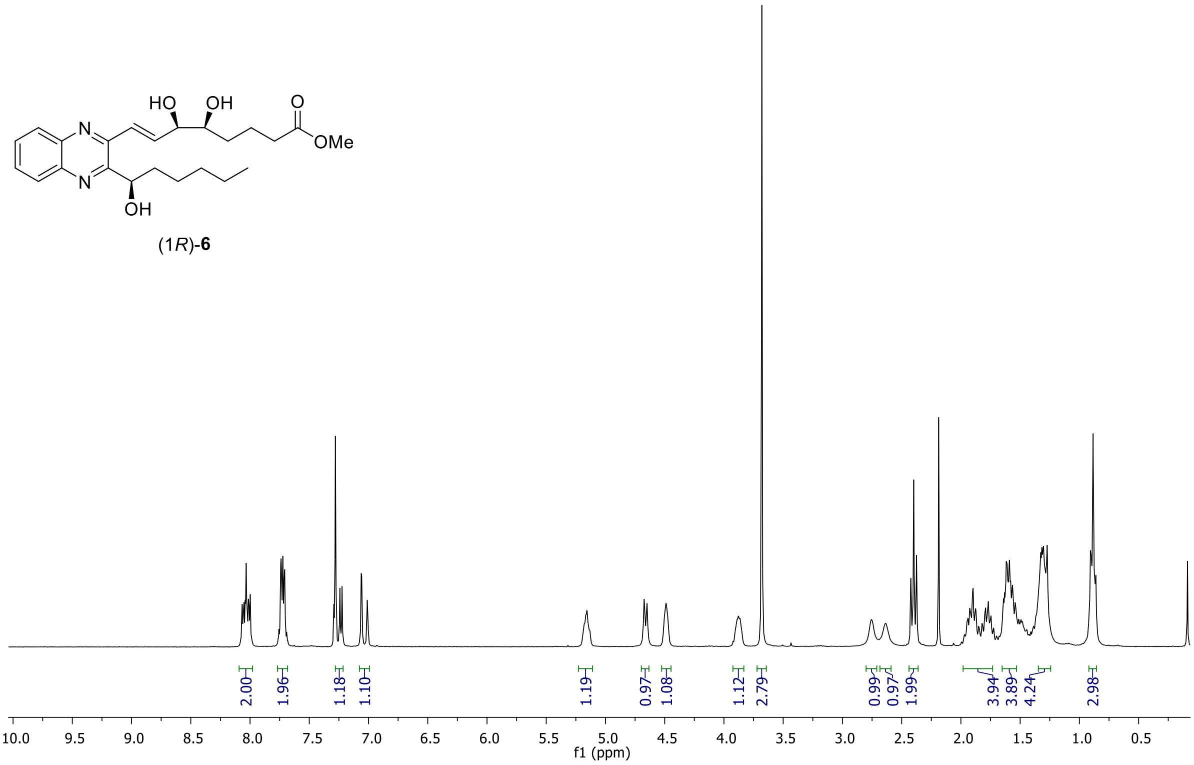

^{13}C NMR (101 MHz, $CDCl_3$)

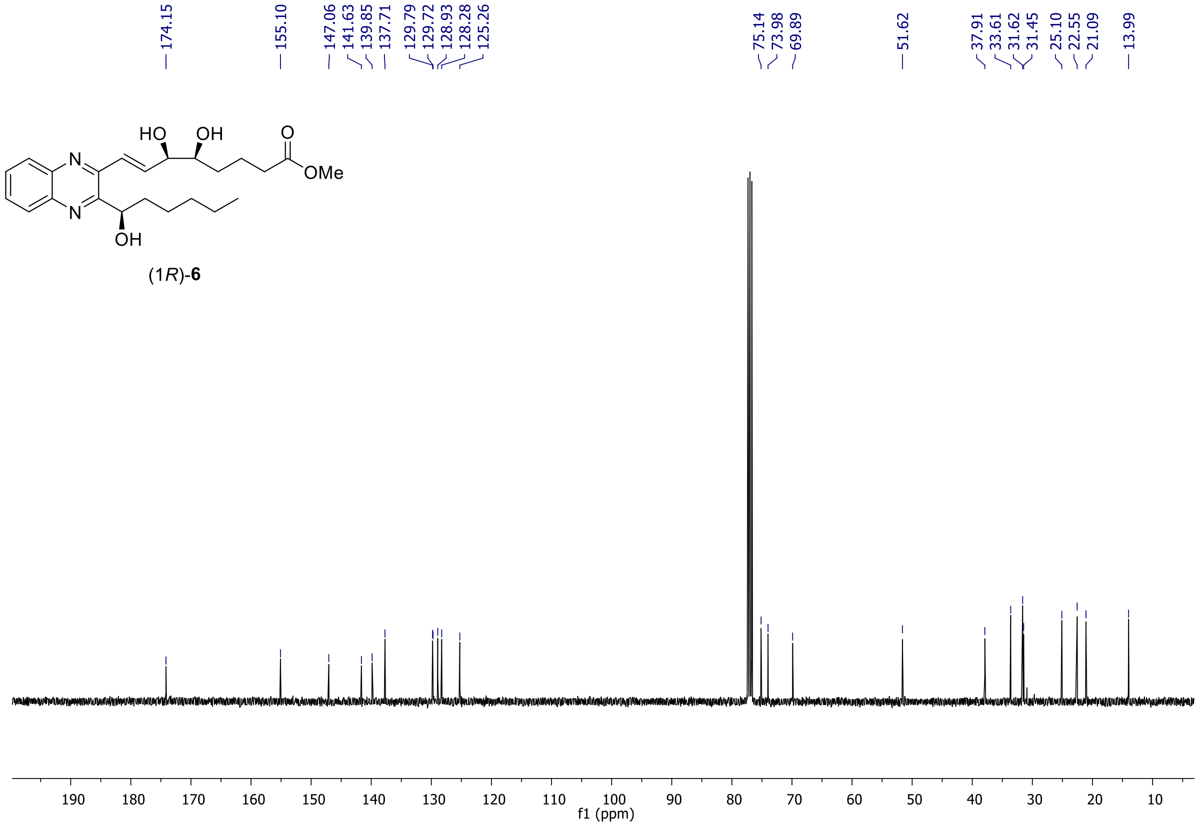

^{1}H NMR (500 MHz, $CDCl_3$)

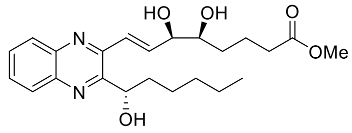

(1*S*)-**6**

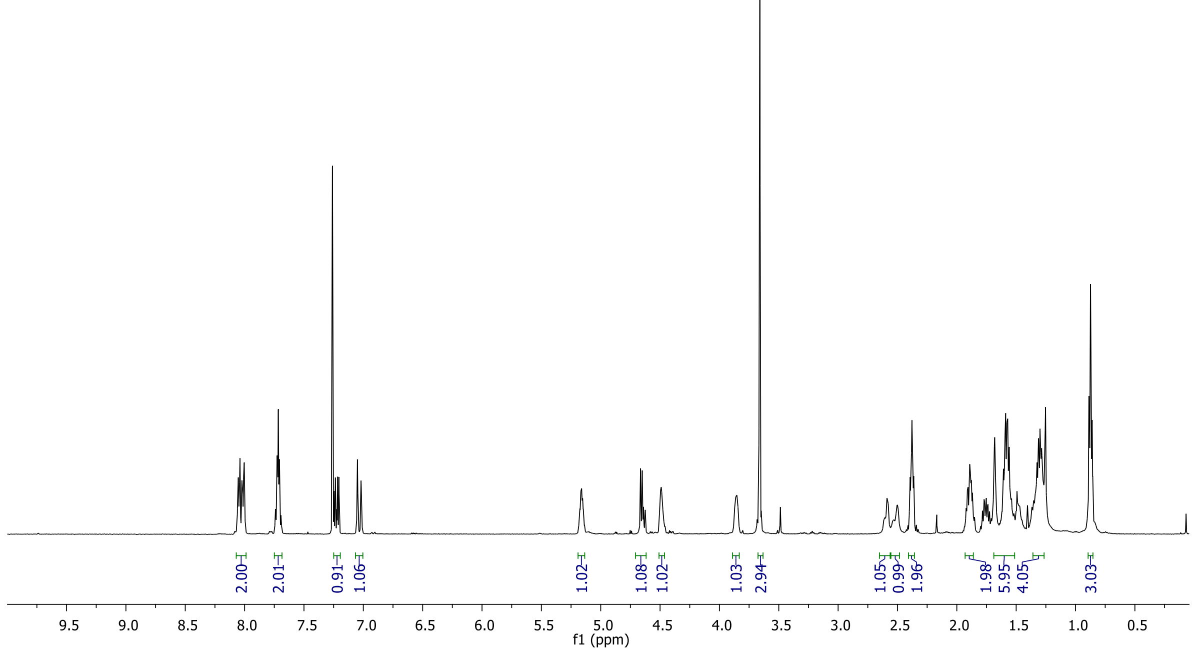

^{13}C NMR (126 MHz, $CDCl_3$)

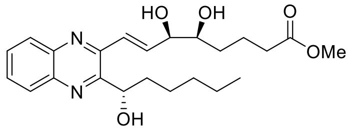

(1*S*)-**6**

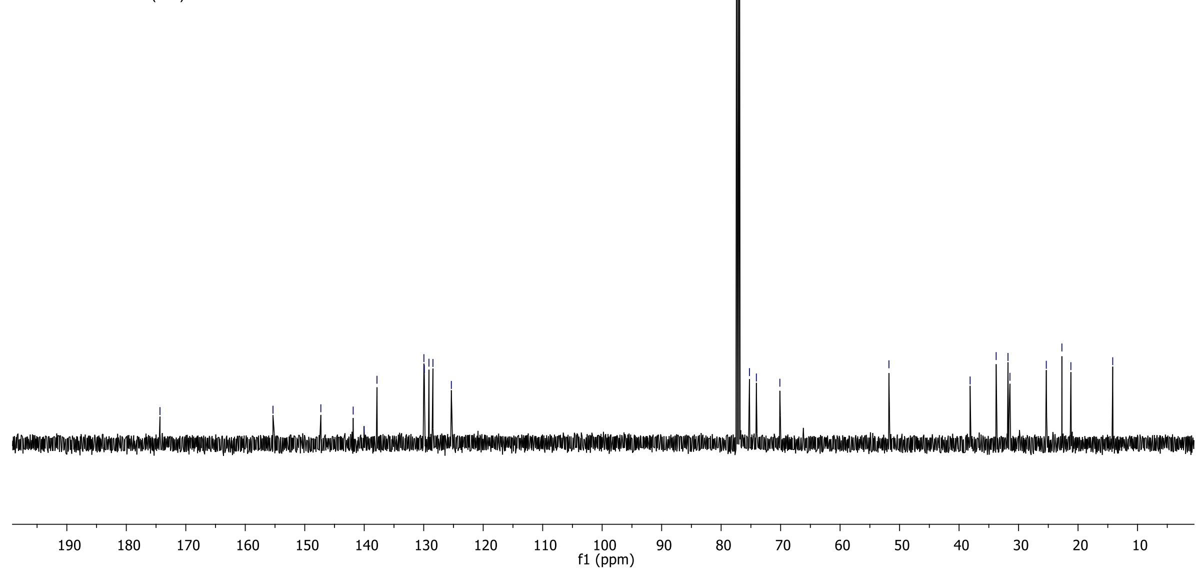

^{1}H NMR (400 MHz, CDCl$_3$)

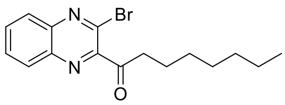

28

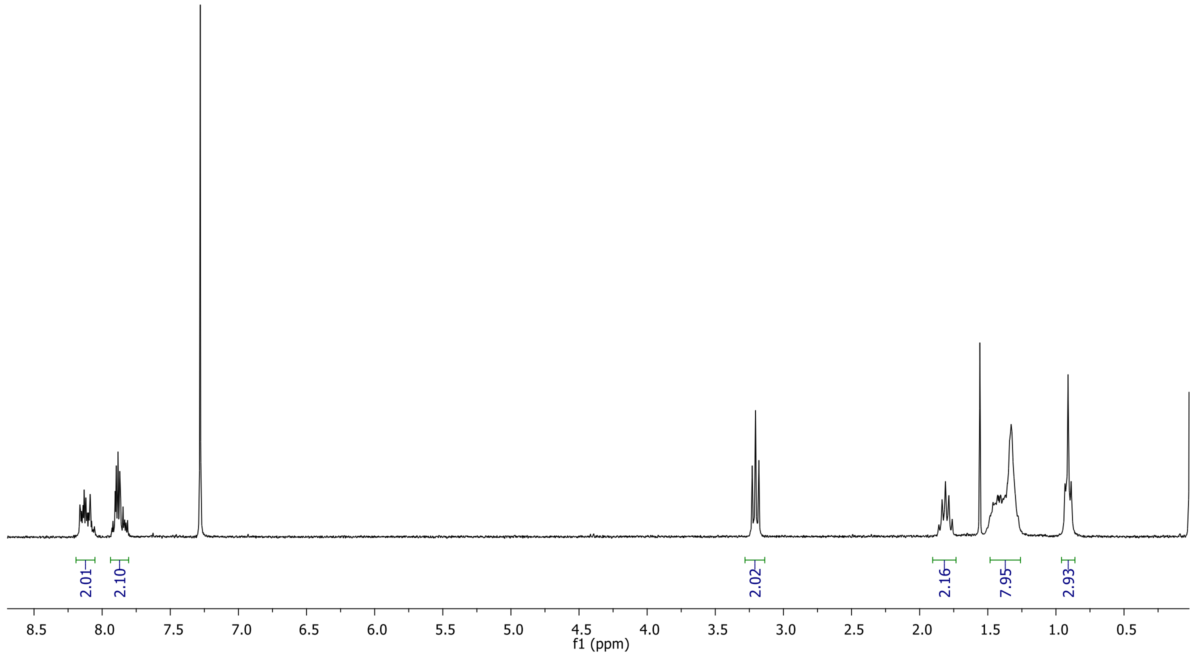

^{13}C NMR (101 MHz, CDCl$_3$)

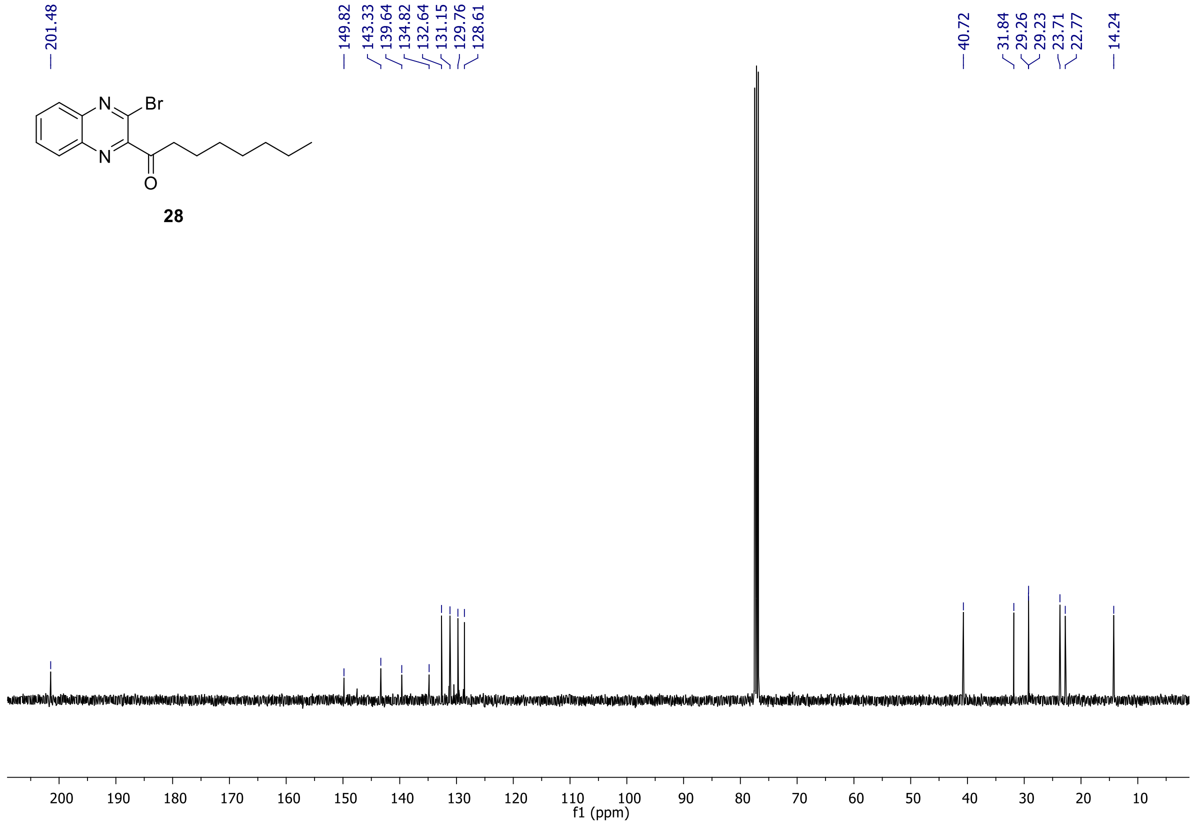

28

^1H NMR (101 MHz, $CDCl_3$)

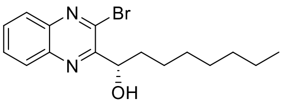

30

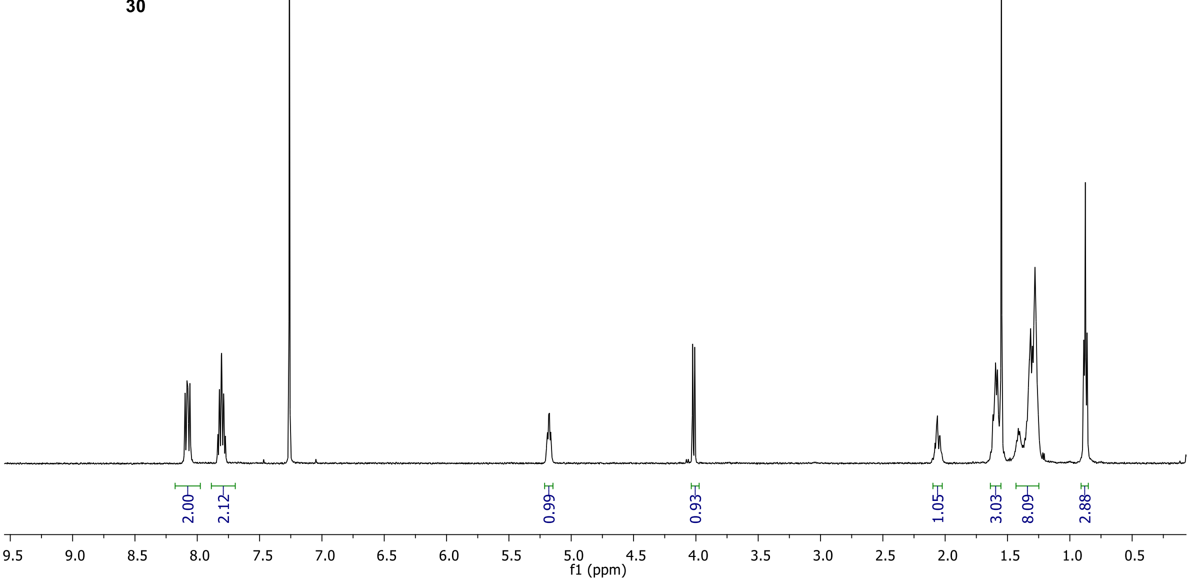

^{13}C NMR (101 MHz, $CDCl_3$)

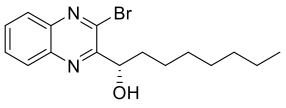

30

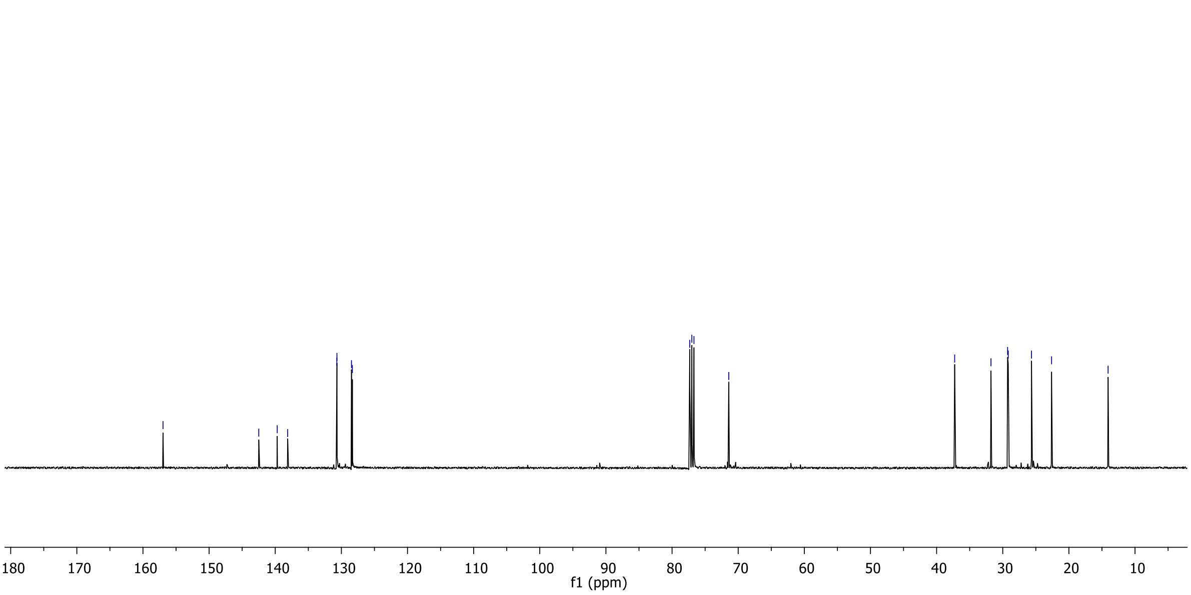

^{1}H NMR (400 MHz, CDCl$_3$)

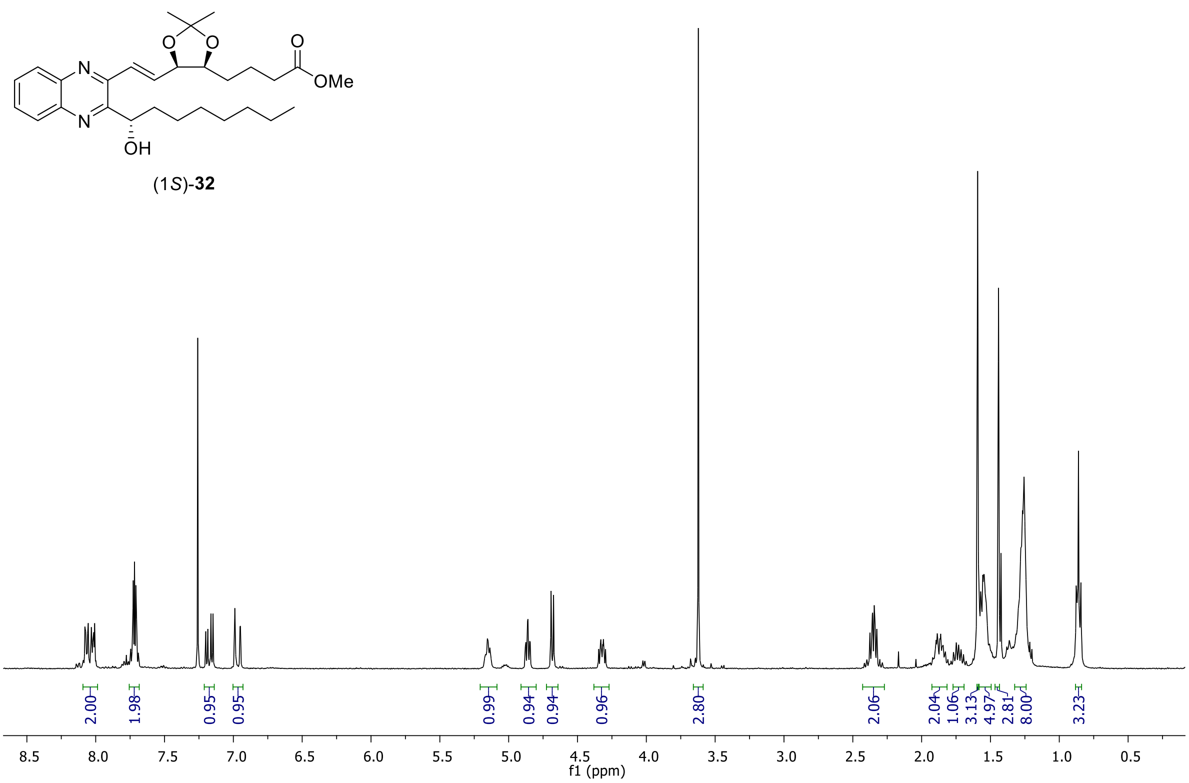

^{13}C NMR (101 MHz, CDCl$_3$)

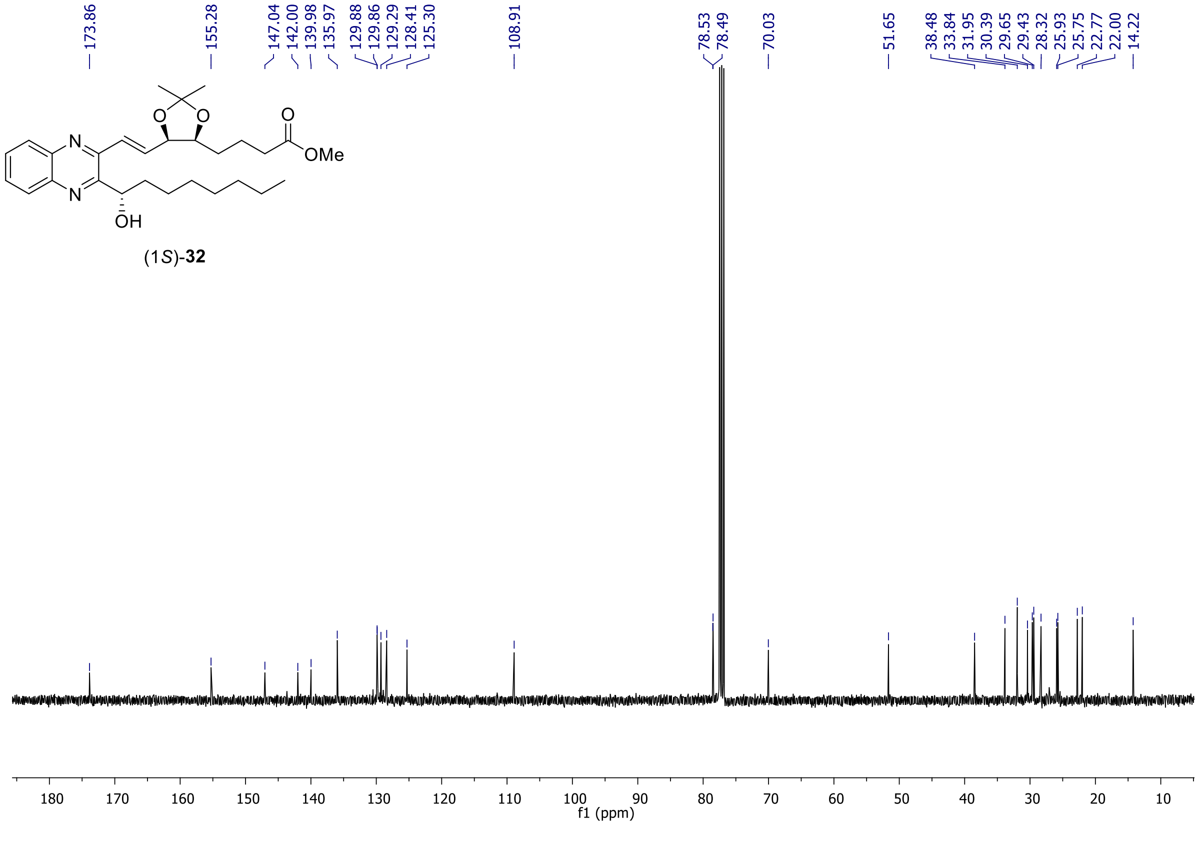

^{1}H NMR (400 MHz, $CDCl_3$)

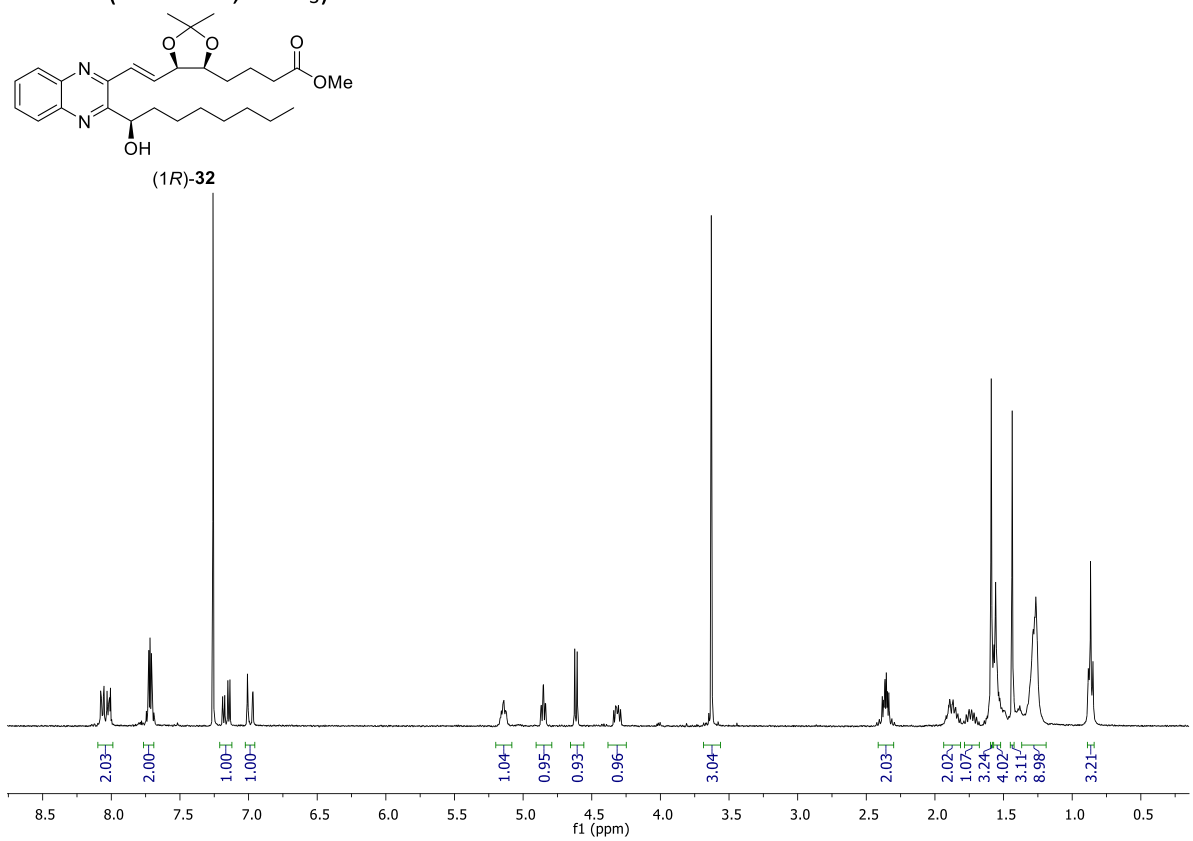

^{13}C NMR (101 MHz, $CDCl_3$)

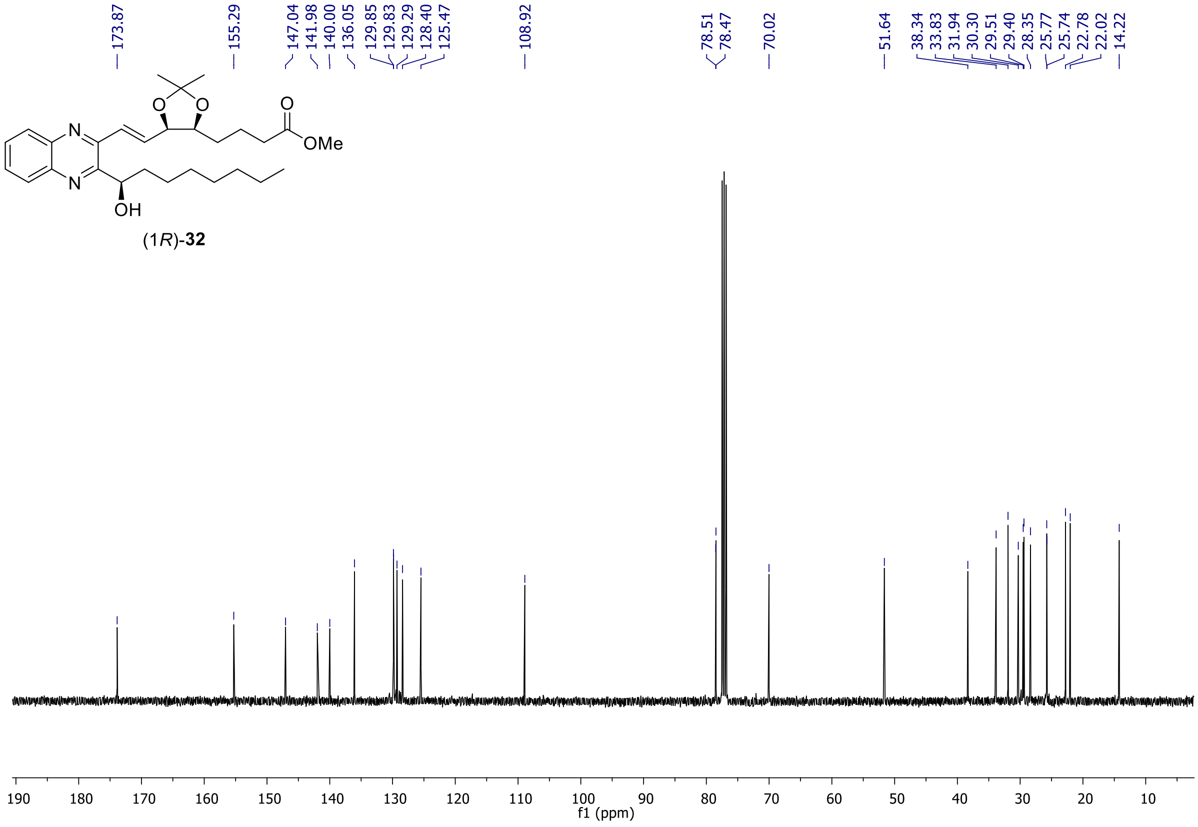

^{1}H NMR (400 MHz, $CDCl_3$)

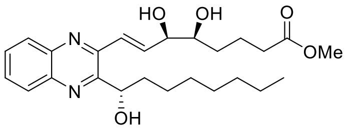

(1*S*)-**8**

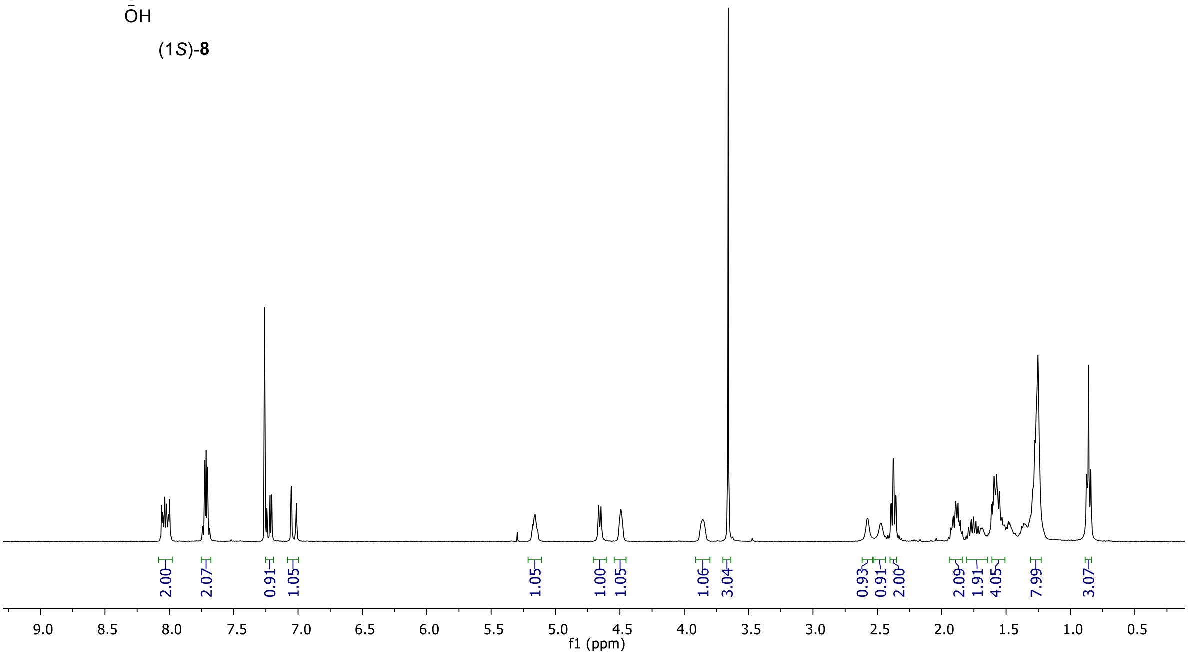

^{13}C NMR (101 MHz, $CDCl_3$)

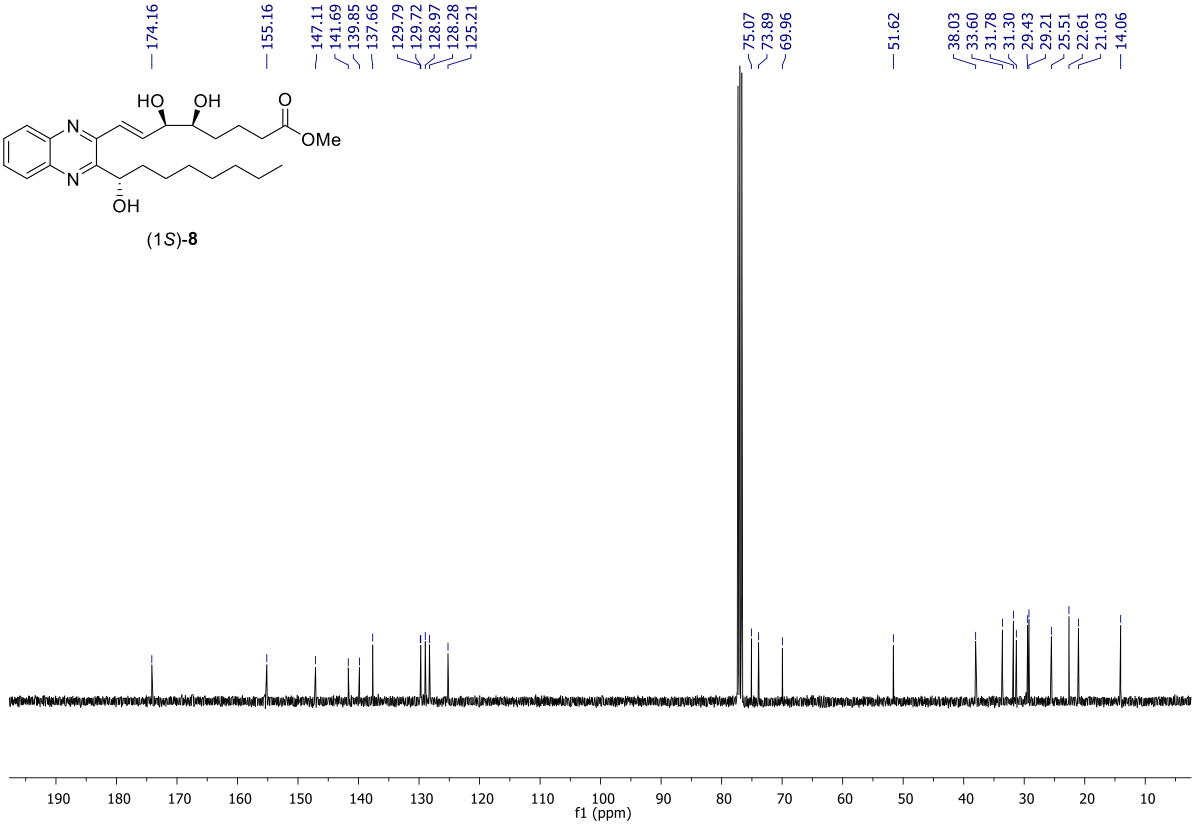

^{1}H NMR (400 MHz, $CDCl_3$)

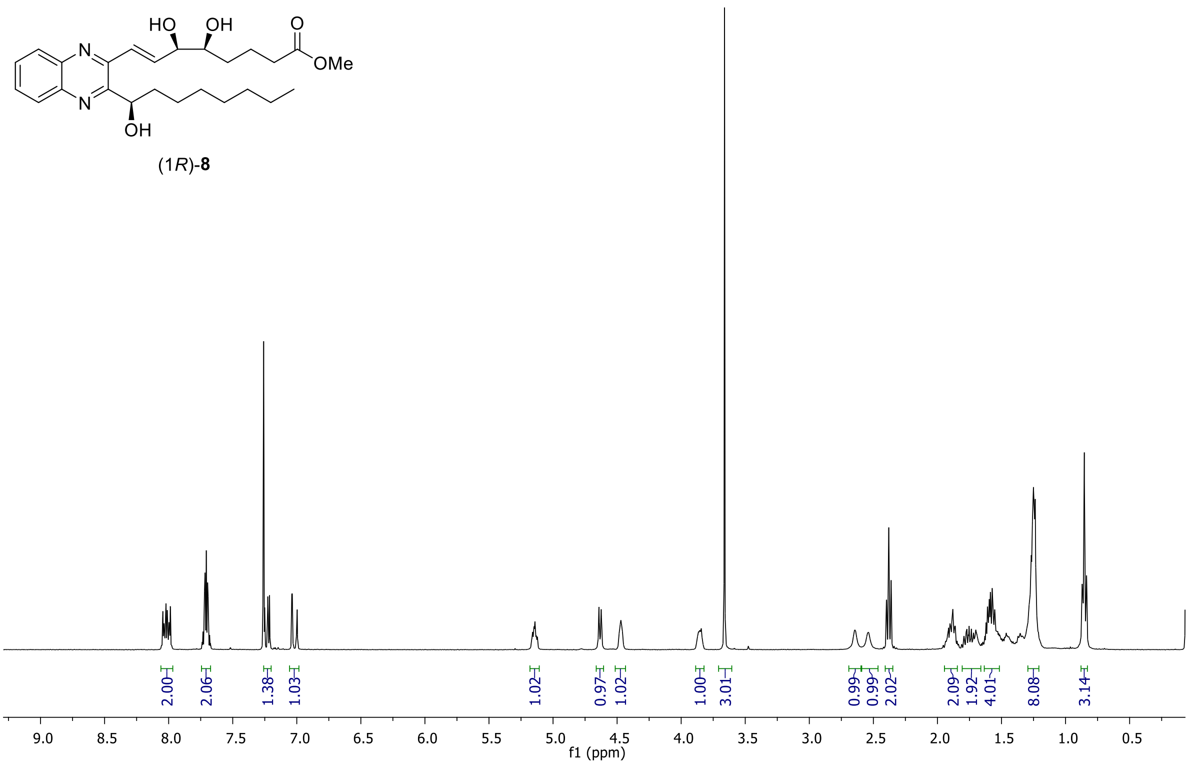

^{13}C NMR (101 MHz, $CDCl_3$)

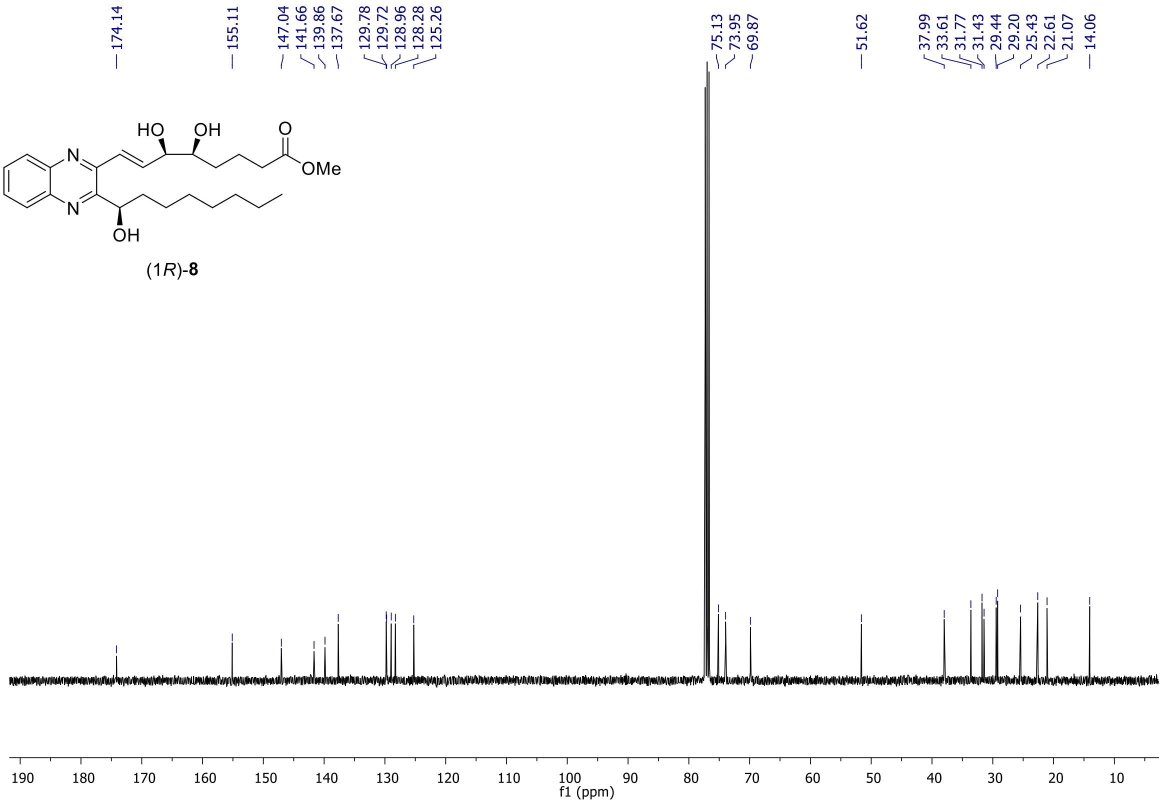

^{1}H NMR (400 MHz, $CDCl_3$)

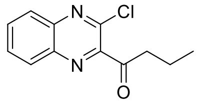

27

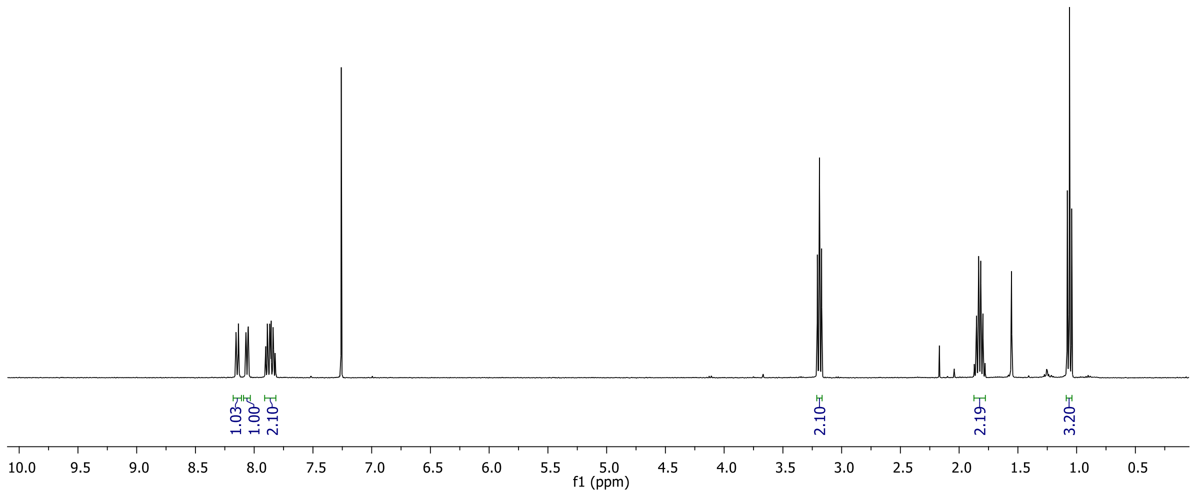

^{13}C NMR (101 MHz, $CDCl_3$)

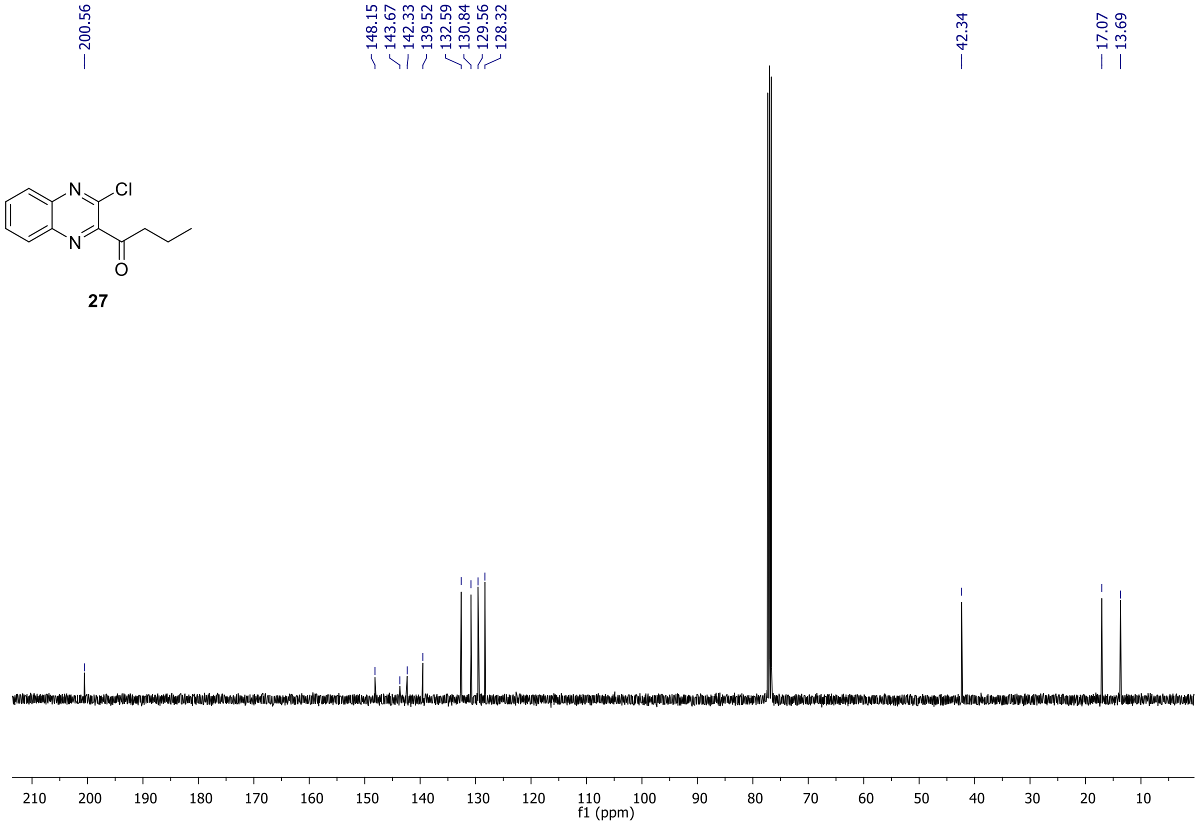

27

^{1}H NMR (400 MHz, $CDCl_3$)

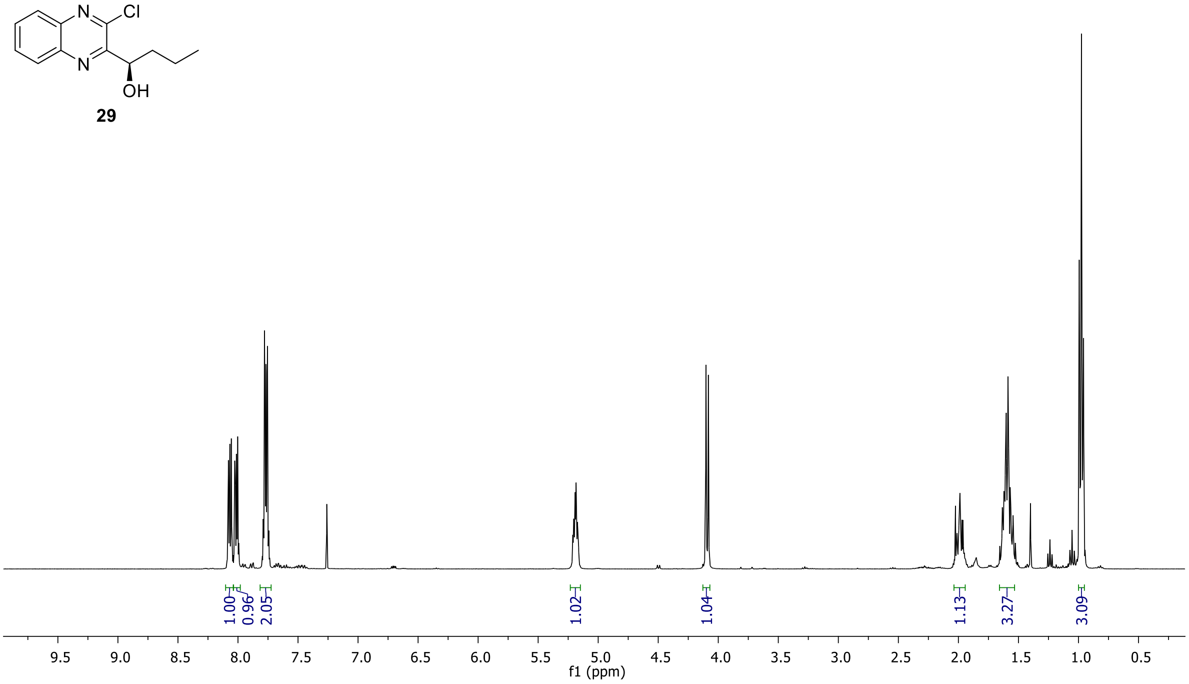

^{13}C NMR (101 MHz, $CDCl_3$)

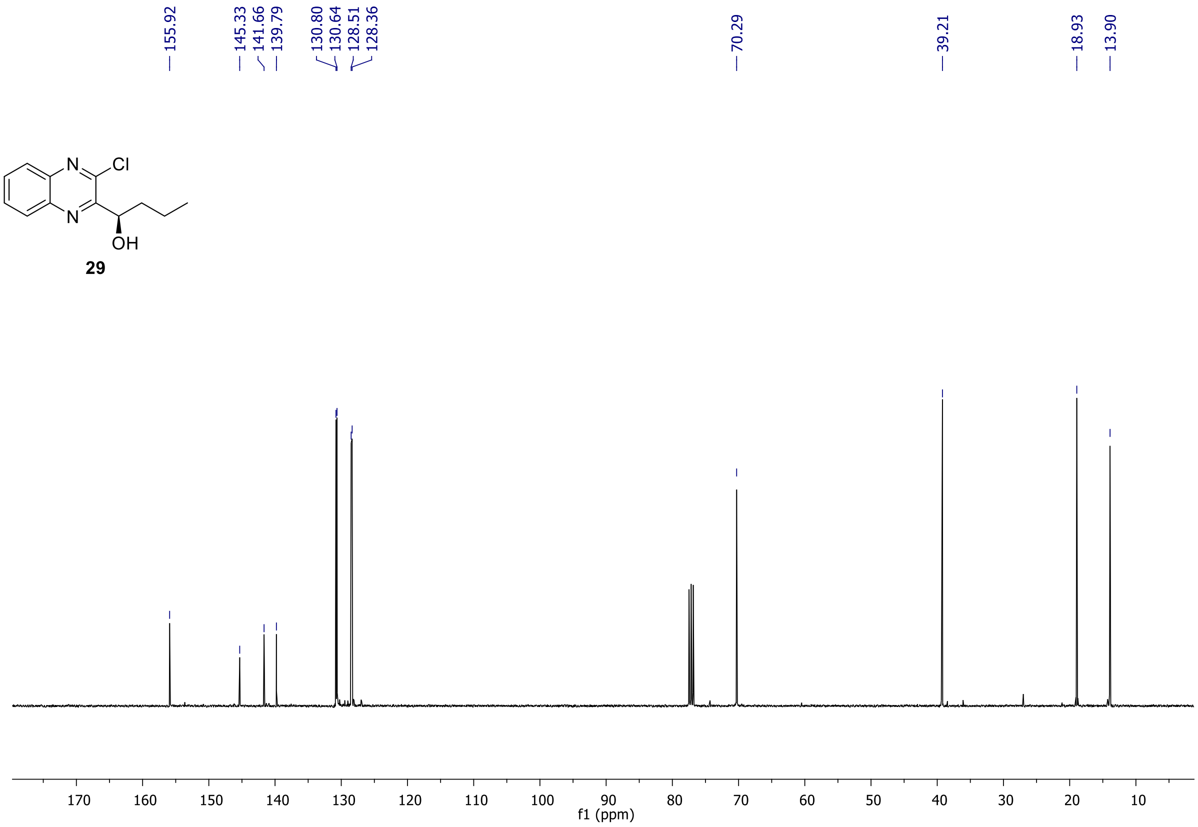

^{1}H NMR (400 MHz, $CDCl_3$)

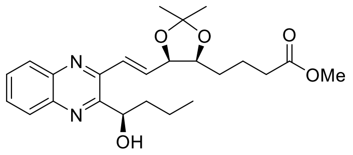

(1*R*)-**31**

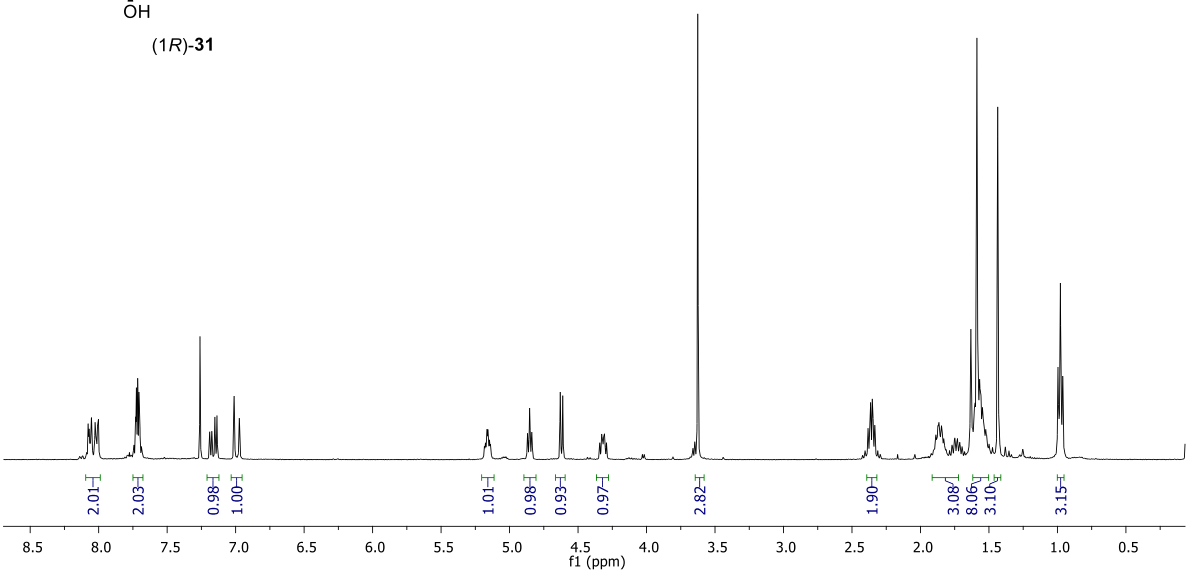

^{13}C NMR (101 MHz, $CDCl_3$)

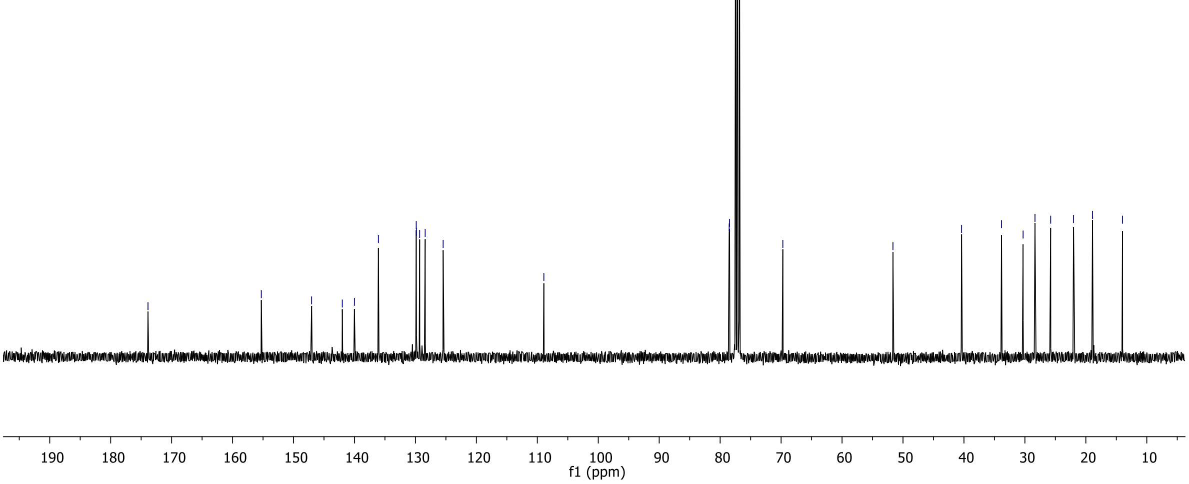

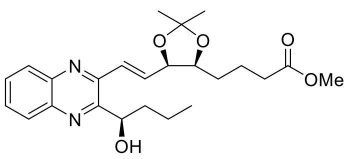

(1*R*)-**31**

^{1}H NMR (400 MHz, $CDCl_3$)

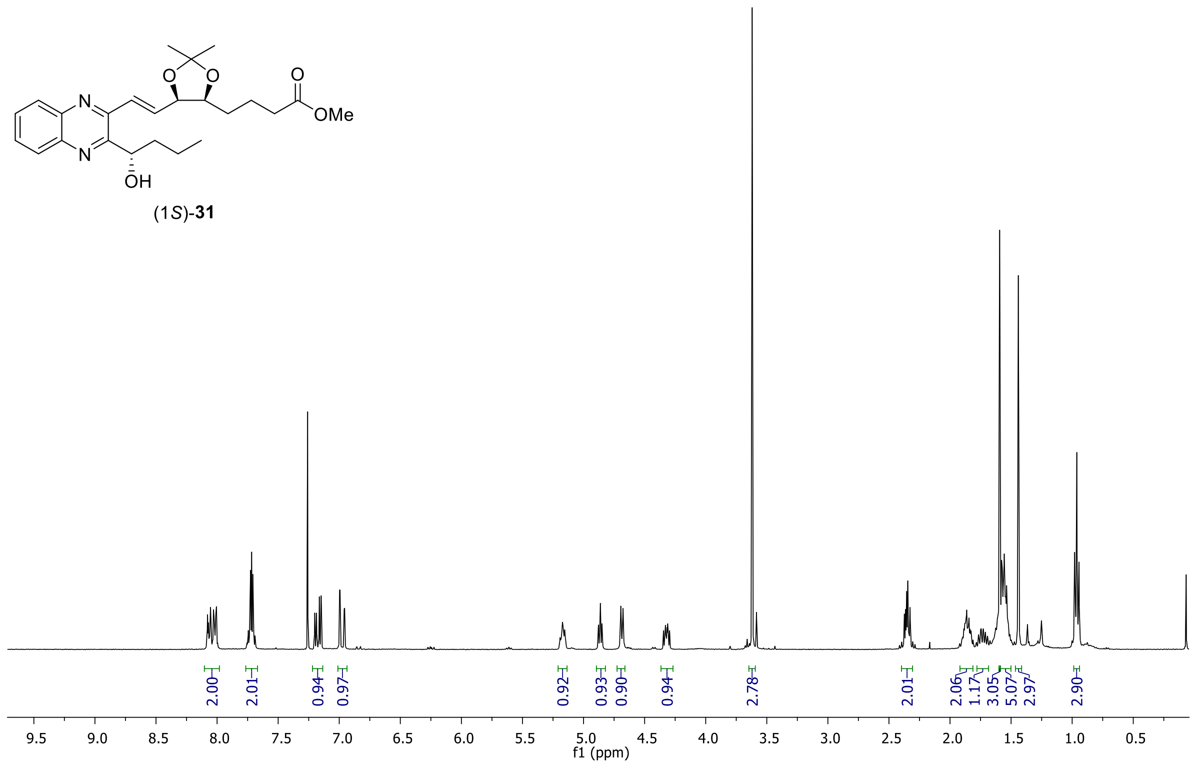

(1*S*)-**31**

^{13}C NMR (101 MHz, $CDCl_3$)

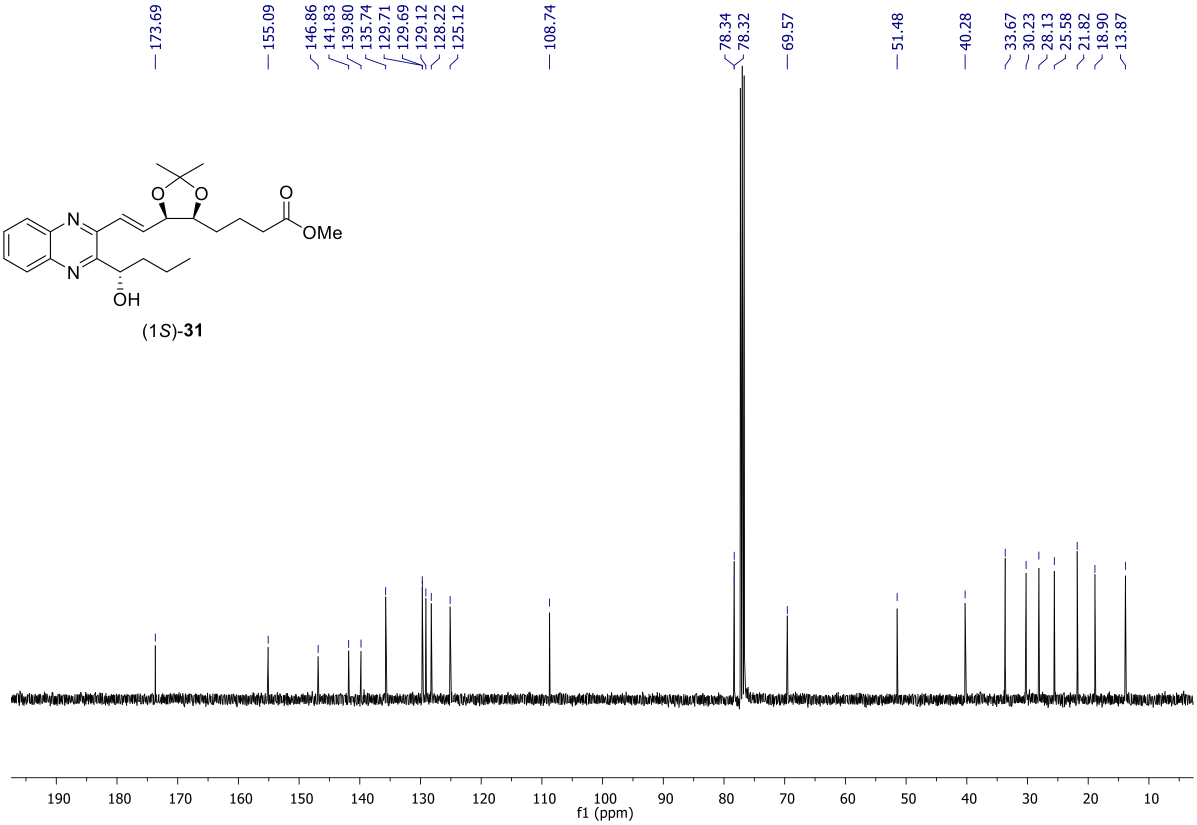

(1*S*)-**31**

^1H NMR (400 MHz, $CDCl_3$)

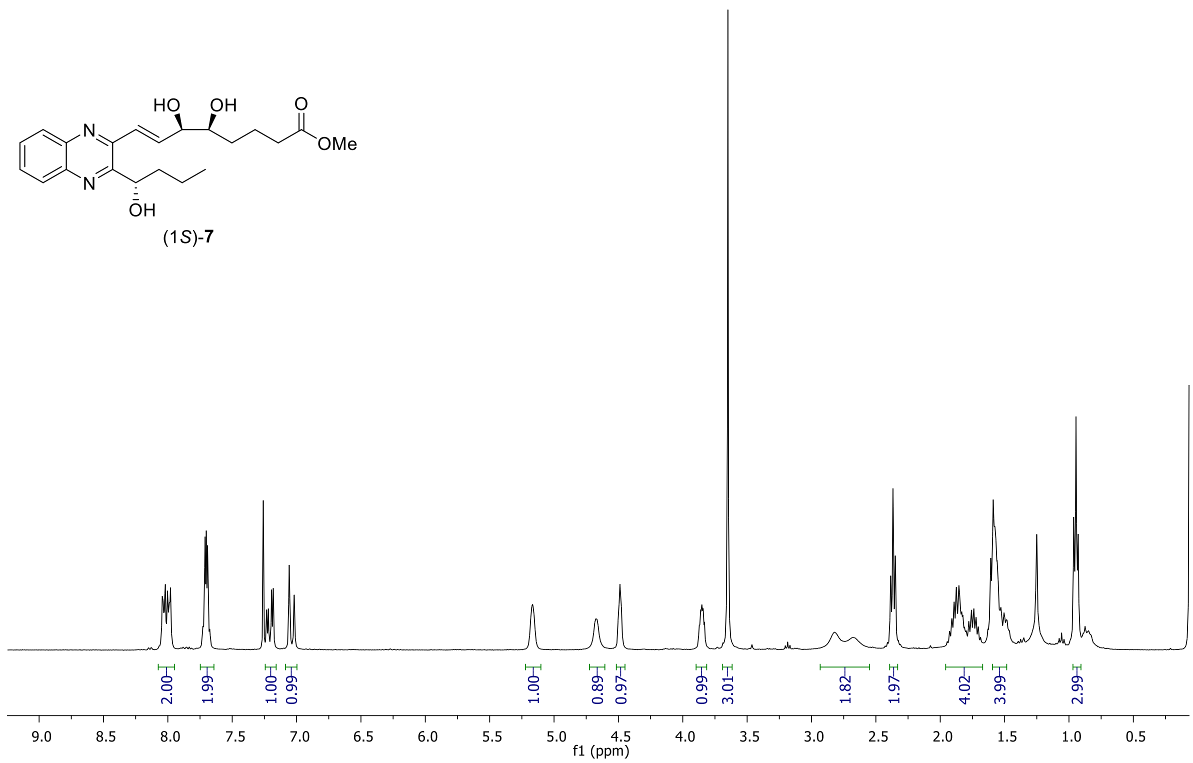

^{13}C NMR (101 MHz, $CDCl_3$)

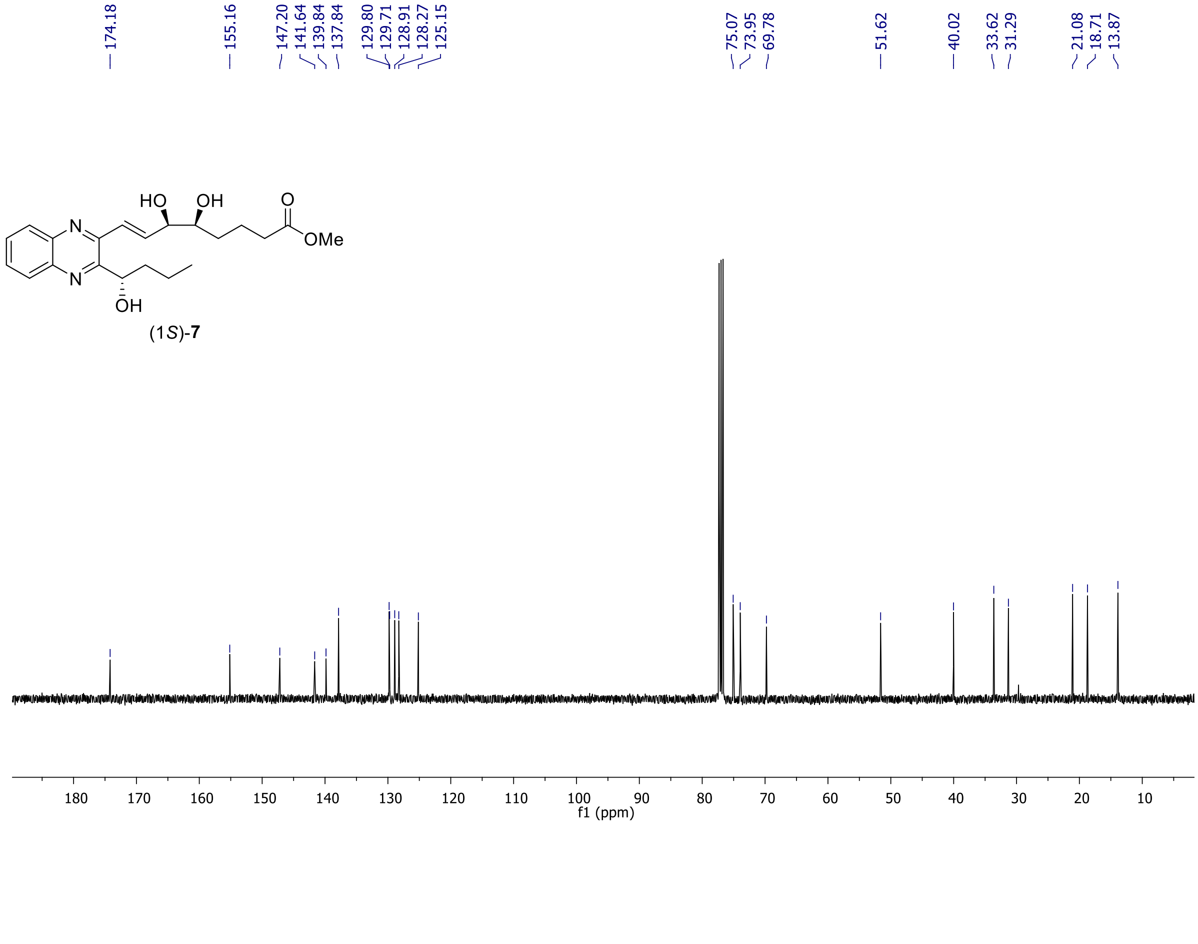

^{1}H NMR (400 MHz, $CDCl_3$)

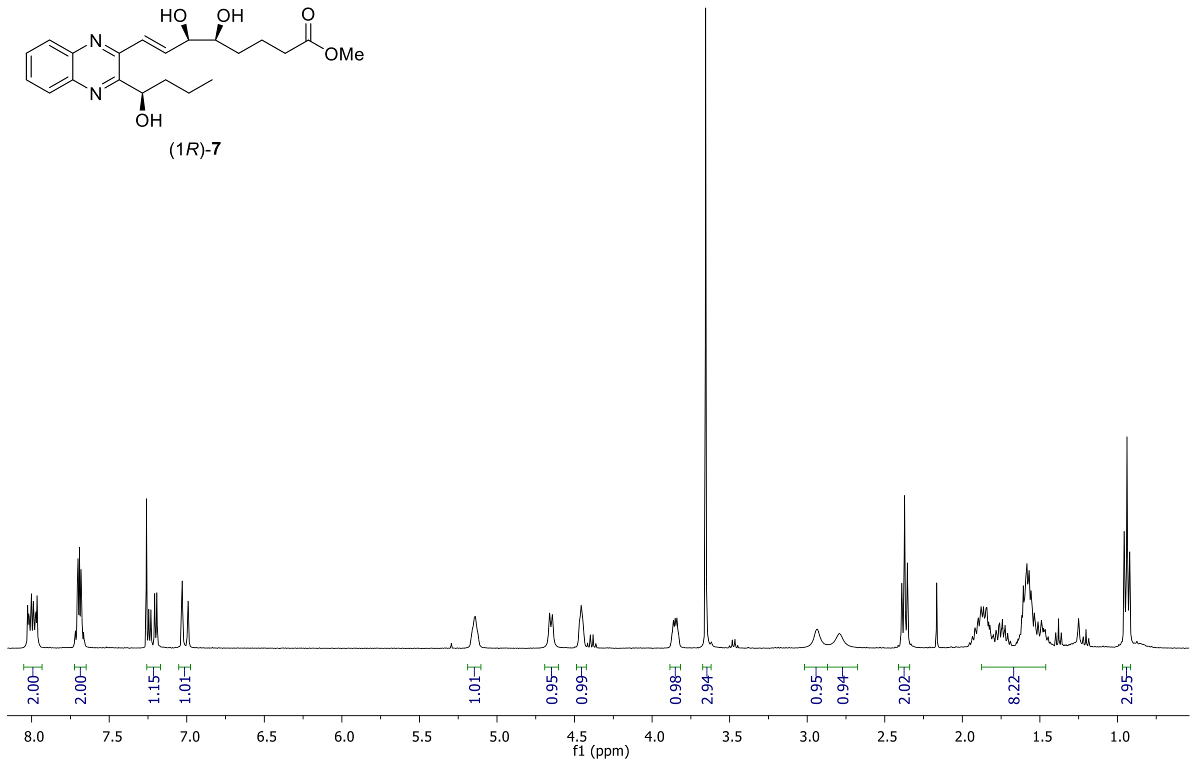

^{13}C NMR (101 MHz, $CDCl_3$)

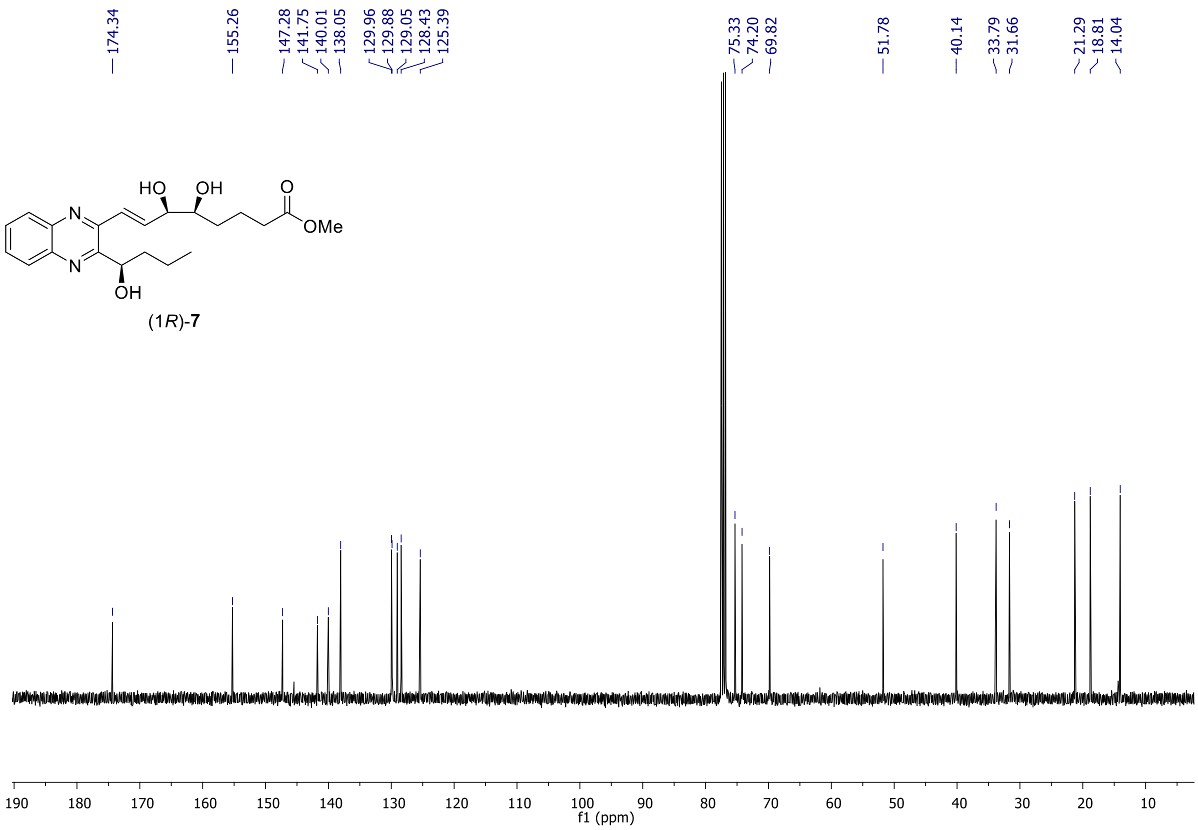

Chromatograms

1-(3-Chloroquinoxalin-2-yl)hexan-1-ol (14)

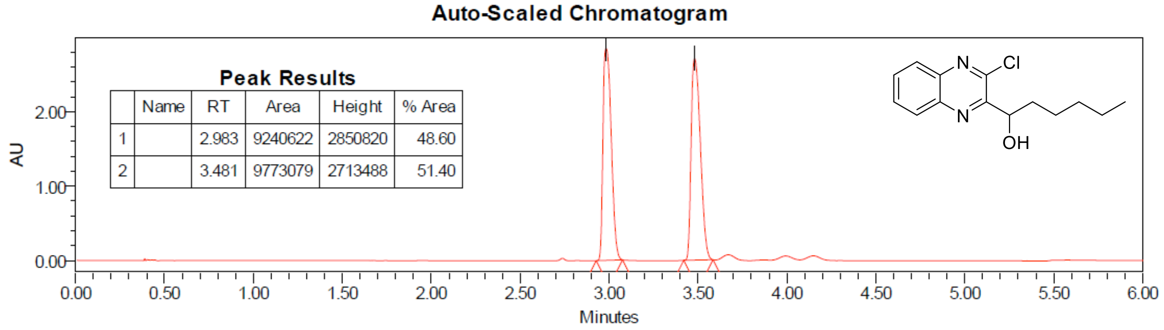

	Name	RT	Area	Height	% Area
1		2.983	9240622	2850820	48.60
2		3.481	9773079	2713488	51.40

(*R*)-1-(3-Chloroquinoxalin-2-yl)hexan-1-ol ((1*R*)-14)

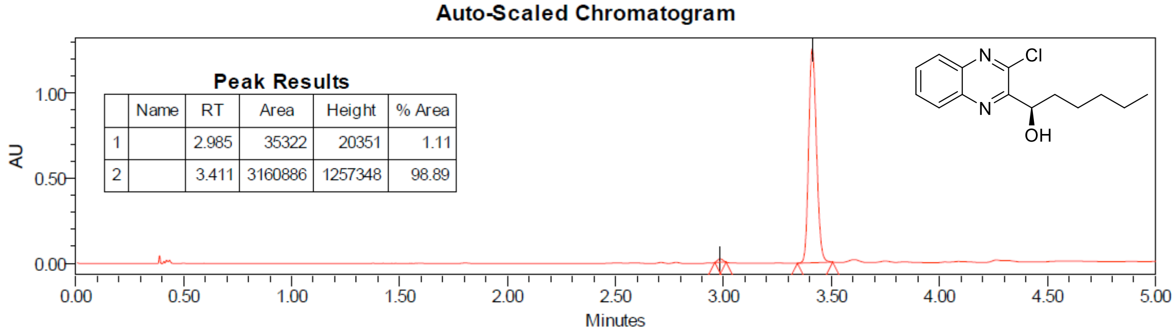

	Name	RT	Area	Height	% Area
1		2.985	35322	20351	1.11
2		3.411	3160886	1257348	98.89

ee = 98% as determined by SFC using a Chiralpak IC column (ACN:CO_2, gradient 99:1 0-1 min, then gradient to 60:40 until 5 min, 3mL/min), R_t = 3.00min (*S*)-enantiomer, 3.41 (*R*)-enantiomer.

(*S*)-1-(3-Chloroquinoxalin-2-yl)hexan-1-ol ((1*S*)-14)

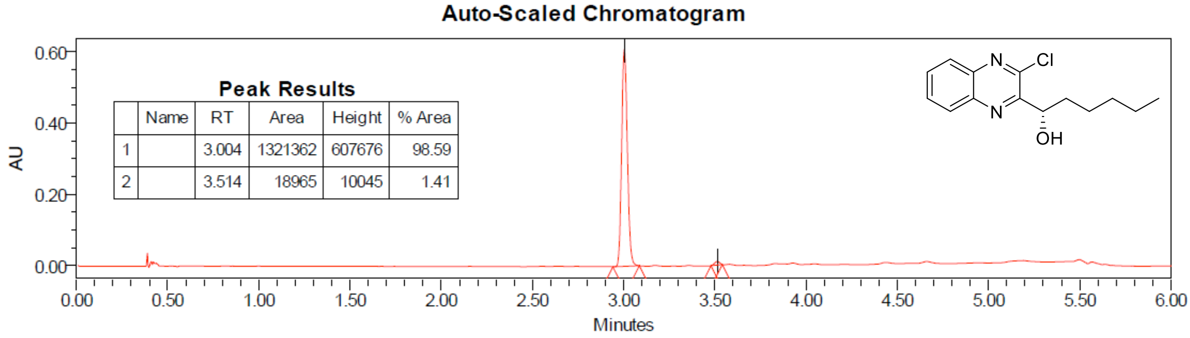

	Name	RT	Area	Height	% Area
1		3.004	1321362	607676	98.59
2		3.514	18965	10045	1.41

ee = 97% as determined by SFC using a Chiralpak IC column (ACN:CO_2, gradient 99:1 0-1 min, then gradient to 60:40 until 5 min, 3mL/min), R_t = 3.00min (*S*)-enantiomer, 3.41 (*R*)-enantiomer.

(*R*)-1-(3-Bromoquinoxalin-2-yl)butan-1-ol ((1*R*)-29)

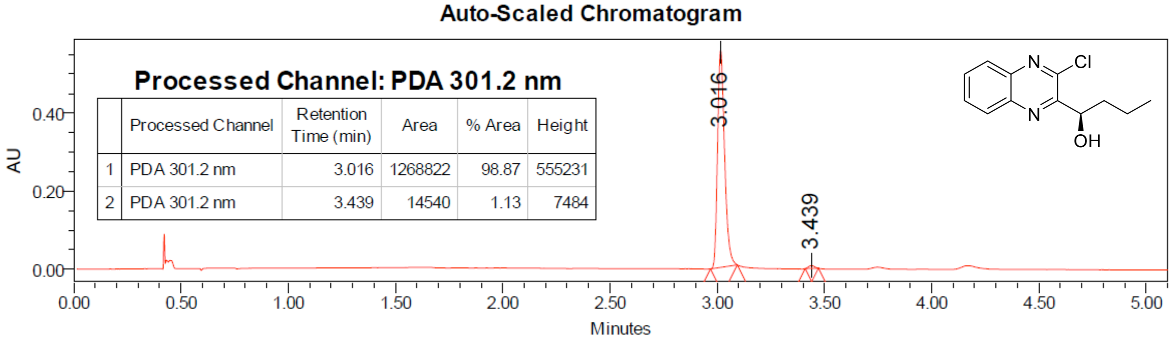

	Processed Channel	Retention Time (min)	Area	% Area	Height
1	PDA 301.2 nm	3.016	1268822	98.87	555231
2	PDA 301.2 nm	3.439	14540	1.13	7484

ee = 98 % as determined by SFC using a Chiralpak IC column (CO_2:ACN, gradient 99:1 0-1 min, then gradient to 60:40 until 5 min, 3mL/min), R_t = 3.02 min (*S*)-enantiomer, 3.45 min (*R*)-enantiomer.

(*S*)-1-(3-Bromoquinoxalin-2-yl)butan-1-ol ((1*S*)-29)

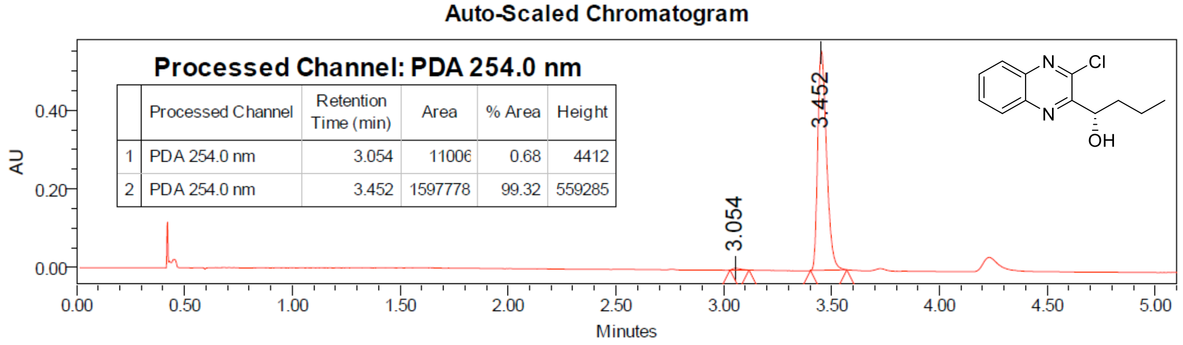

	Processed Channel	Retention Time (min)	Area	% Area	Height
1	PDA 254.0 nm	3.054	11006	0.68	4412
2	PDA 254.0 nm	3.452	1597778	99.32	559285

ee = 98 % as determined by SFC using a Chiralpak IC column (CO_2:ACN, gradient 99:1 0-1 min, then gradient to 60:40 until 5 min, 3mL/min), R_t = 3.02 min (*S*)-enantiomer, 3.45 min (*R*)-enantiomer.

(*R*)-1-(3-Bromoquinoxalin-2-yl)octan-1-ol ((1*R*)-30)

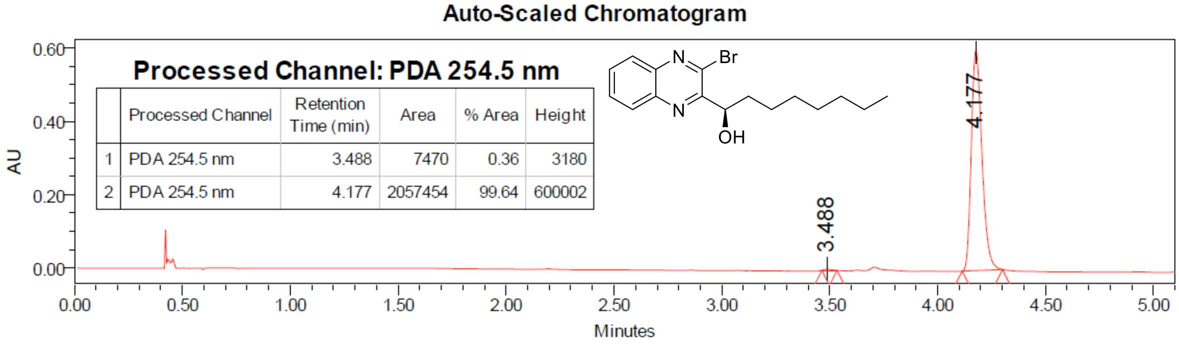

	Processed Channel	Retention Time (min)	Area	% Area	Height
1	PDA 254.5 nm	3.488	7470	0.36	3180
2	PDA 254.5 nm	4.177	2057454	99.64	600002

ee = 99 % as determined by SFC using a Chiralpak IC column (CO_2:ACN, gradient 99:1 0-1 min, then gradient to 60:40 until 5 min, 3mL/min), R_t = 3.43min (*S*)-enantiomer, 4.18 min (*R*)-enantiomer.

(*S*)-1-(3-Bromoquinoxalin-2-yl)octan-1-ol ((1*S*)-30)

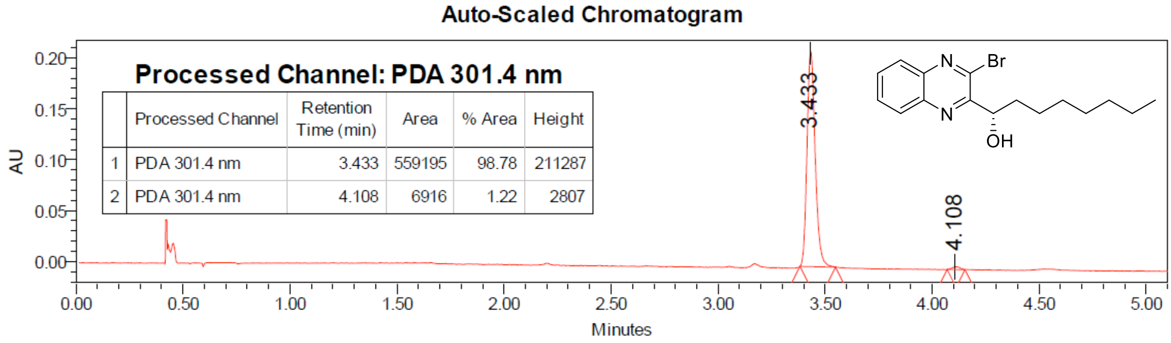

ee = 98 % as determined by SFC using a Chiralpak IC column (CO_2:ACN, gradient 99:1 0-1 min, then gradient to 60:40 until 5 min, 3mL/min), R_t = 3.43 min (*S*)-enantiomer, 4.18 min (*R*)-enantiomer.

Chromatograms of analogues submitted for biological evaluation:

Method: Purity determined by SFC using a Chiralpak IC column (MeCN:CO_2, gradient 99:1 0-1 min, then gradient to 60:40 until 10 min, 3mL/min),

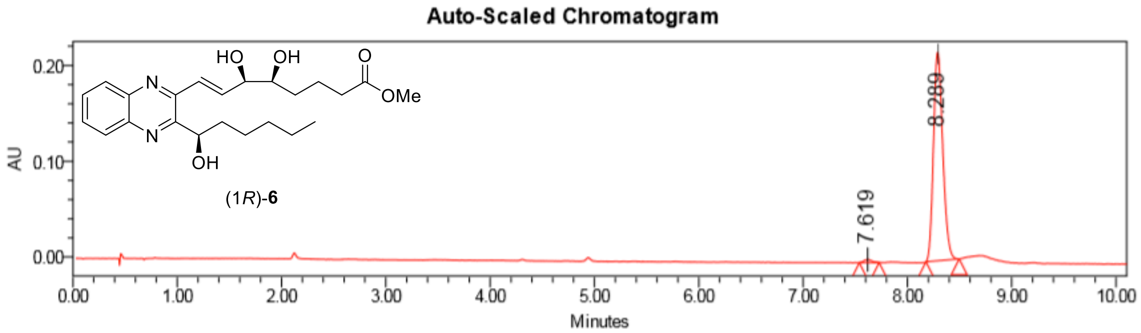

Processed Channel: PDA 281.0 nm

	Processed Channel	Retention Time (min)	Area	% Area	Height
1	PDA 281.0 nm	7.619	16398	1.20	3212
2	PDA 281.0 nm	8.289	1345165	98.80	217536

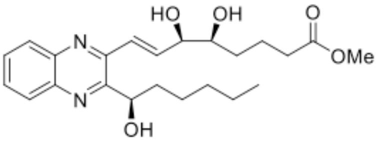

(1*R*)-**6**

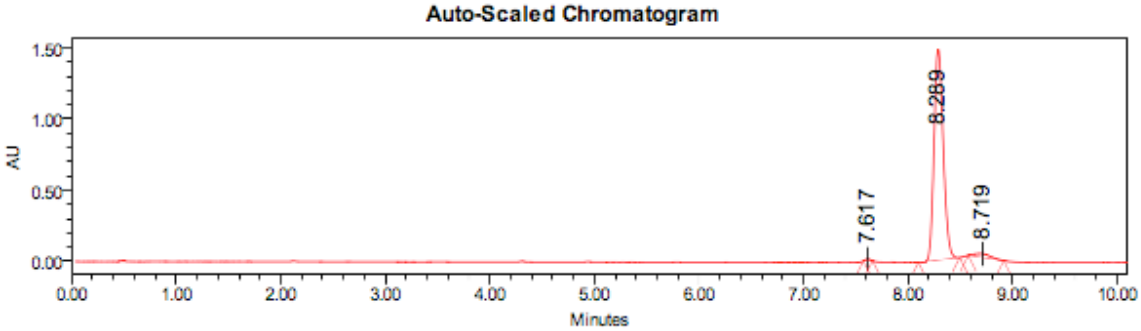

Processed Channel: PDA 254.0 nm

	Processed Channel	Retention Time (min)	Area	% Area	Height
1	PDA 254.0 nm	7.617	38344	0.41	1193[illegible]
2	PDA 254.0 nm	8.289	9016729	96.57	1480195
3	PDA 254.0 nm	8.719	281965	3.02	26952

Auto-Scaled Chromatogram

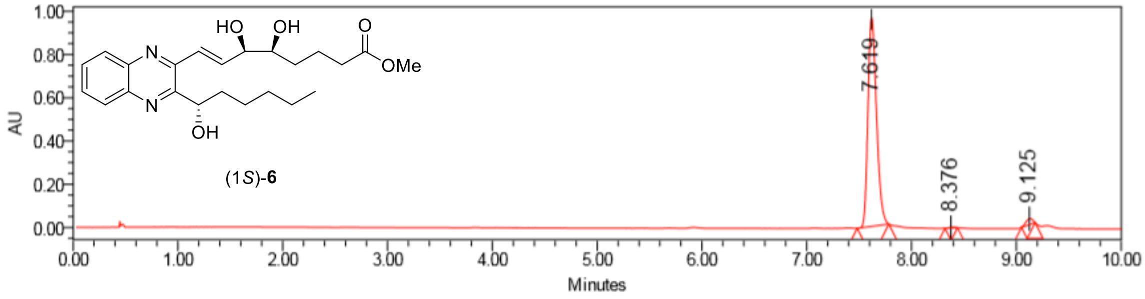

Processed Channel: PDA 268.5 nm

	Processed Channel	Retention Time (min)	Area	% Area	Height
1	PDA 268.5 nm	7.619	5452089	97.61	965491
2	PDA 268.5 nm	8.376	12305	0.22	2967
3	PDA 268.5 nm	9.125	121136	2.17	27646

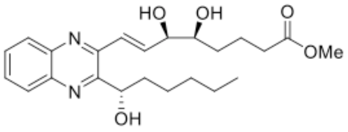

(1*S*)-**6**

Auto-Scaled Chromatogram

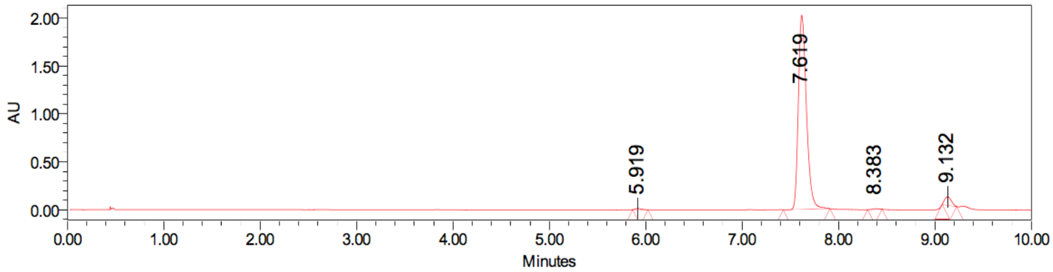

Processed Channel: PDA 254.0 nm

	Processed Channel	Retention Time (min)	Area	% Area	Height
1	PDA 254.0 nm	5.919	53709	0.44	11891
2	PDA 254.0 nm	7.619	11775558	95.81	2022987
3	PDA 254.0 nm	8.383	35860	0.29	7423
4	PDA 254.0 nm	9.132	425216	3.46	88540

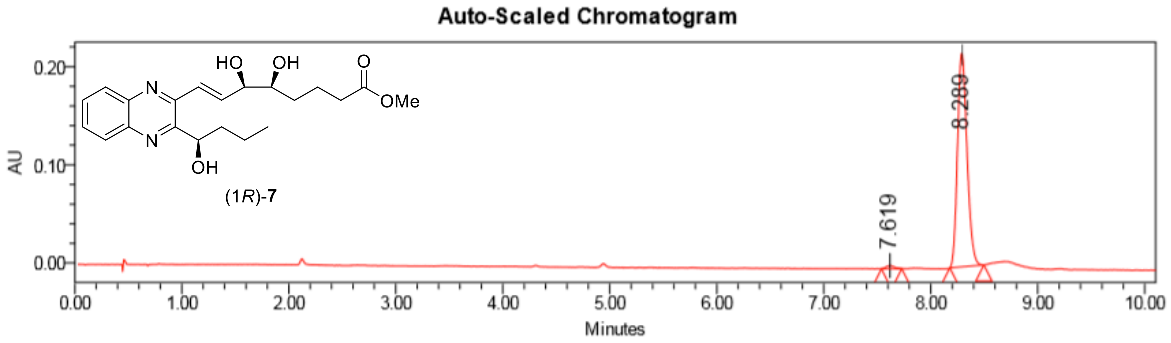

Processed Channel: PDA 281.0 nm

	Processed Channel	Retention Time (min)	Area	% Area	Height
1	PDA 281.0 nm	7.619	16398	1.20	3212
2	PDA 281.0 nm	8.289	1345165	98.80	217536

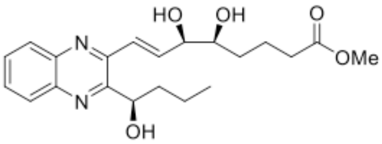

(1*R*)-**7**

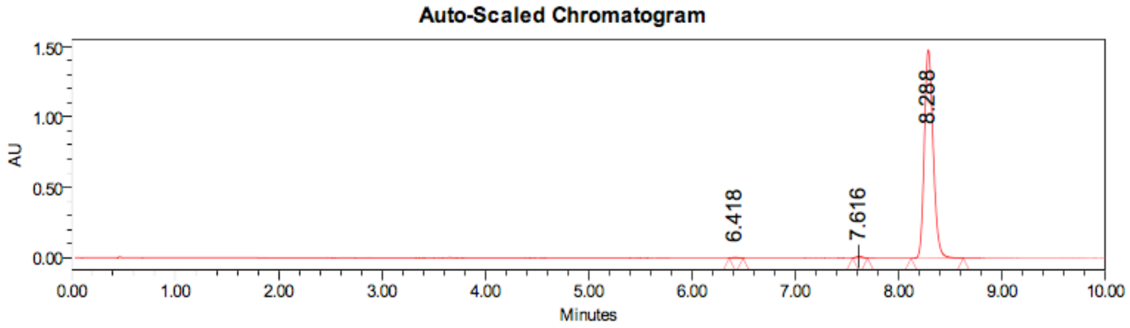

Processed Channel: PDA 254.0 nm

	Processed Channel	Retention Time (min)	Area	% Area	Height
1	PDA 254.0 nm	6.418	22887	0.26	5274
2	PDA 254.0 nm	7.616	53820	0.61	12150
3	PDA 254.0 nm	8.288	8803825	99.14	1481634

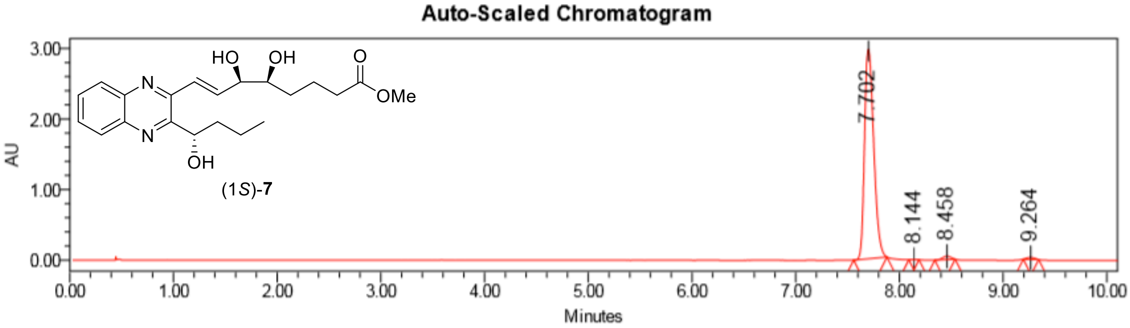

Processed Channel: PDA 254.0 nm

	Processed Channel	Retention Time (min)	Area	% Area	Height
1	PDA 254.0 nm	7.702	18243920	97.76	2971477
2	PDA 254.0 nm	8.144	13637	0.07	3832
3	PDA 254.0 nm	8.458	269875	1.45	48605
4	PDA 254.0 nm	9.264	134743	0.72	26059

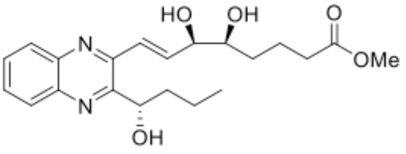

(1*S*)-**7**

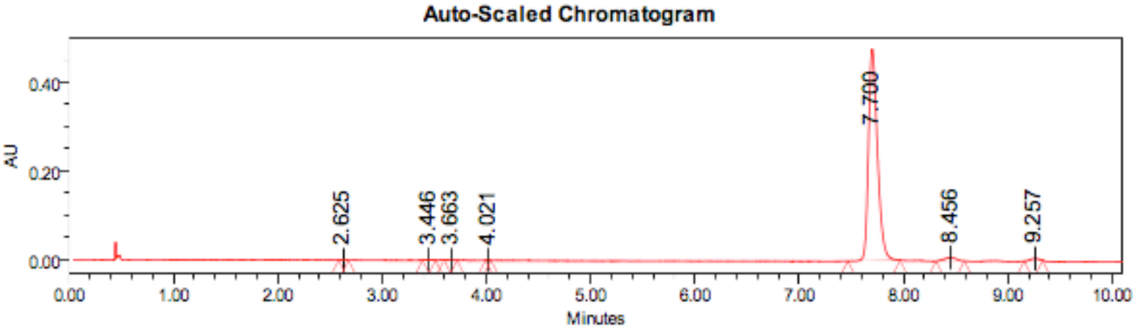

Processed Channel: PDA 281.0 nm

	Processed Channel	Retention Time (min)	Area	% Area	Height
1	PDA 281.0 nm	2.625	1209	0.04	384
2	PDA 281.0 nm	3.446	5585	0.19	1497
3	PDA 281.0 nm	3.663	2385	0.08	663
4	PDA 281.0 nm	4.021	1310	0.04	802
5	PDA 281.0 nm	7.700	2837188	96.54	477519
6	PDA 281.0 nm	8.456	61807	2.10	8215
7	PDA 281.0 nm	9.257	29455	1.00	5238

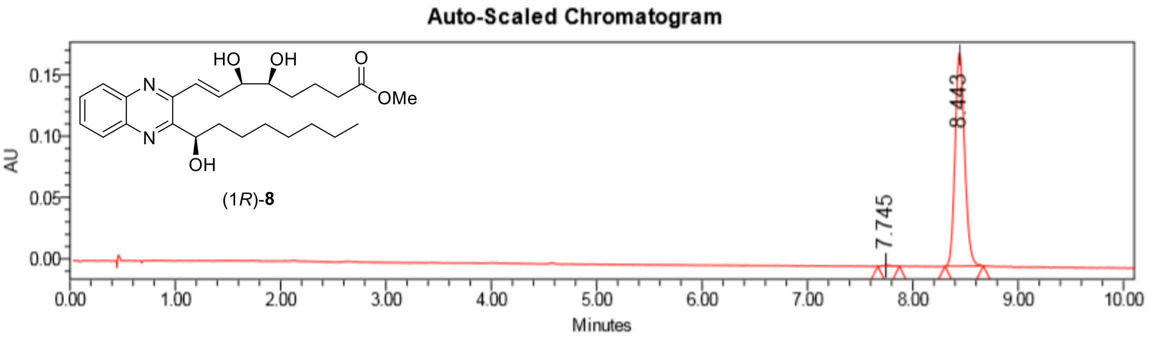

Processed Channel: PDA 281.0 nm

	Processed Channel	Retention Time (min)	Area	% Area	Height
1	PDA 281.0 nm	7.745	4270	0.39	848
2	PDA 281.0 nm	8.443	1095276	99.61	173775

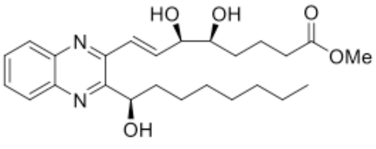

(1*R*)-**8**

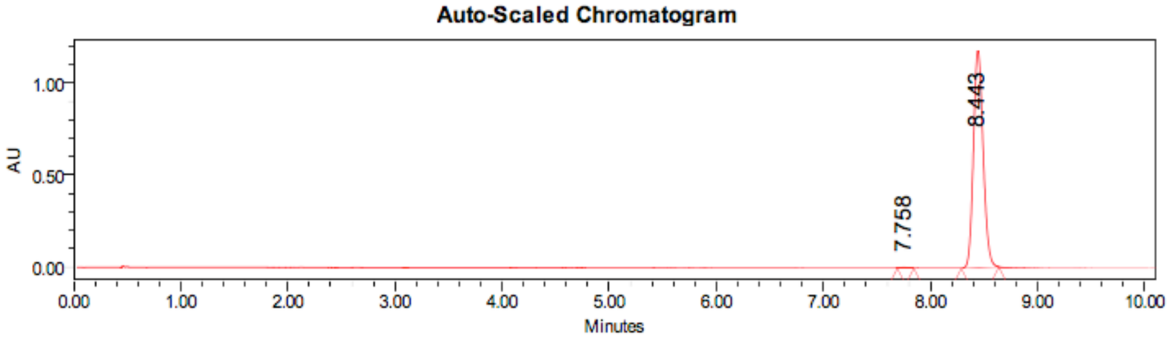

Processed Channel: PDA 254.0 nm

	Processed Channel	Retention Time (min)	Area	% Area	Height
1	PDA 254.0 nm	7.758	21538	0.29	4540
2	PDA 254.0 nm	8.443	7397535	99.71	1181227

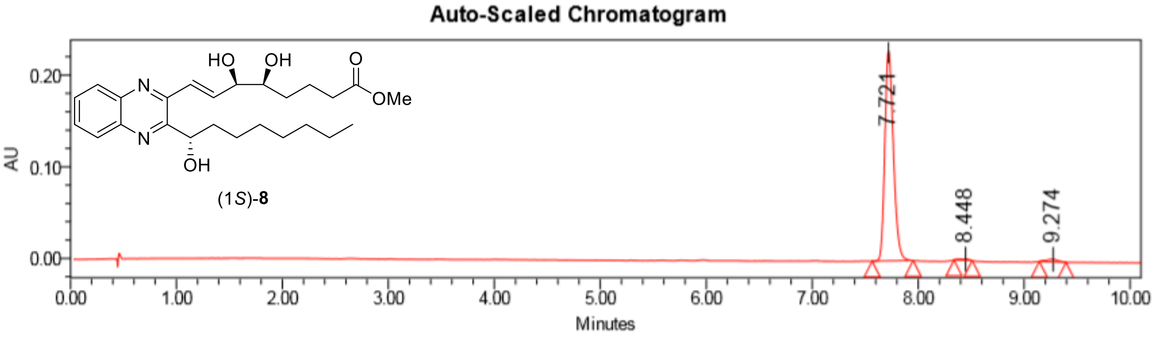

Processed Channel: PDA 281.0 nm

	Processed Channel	Retention Time (min)	Area	% Area	Height
1	PDA 281.0 nm	7.721	1283251	97.62	229069
2	PDA 281.0 nm	8.448	9185	0.70	1379
3	PDA 281.0 nm	9.274	22046	1.68	2778

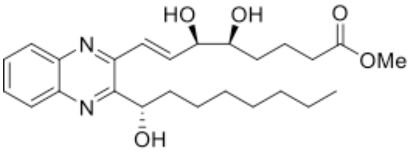

(1*S*)-**8**

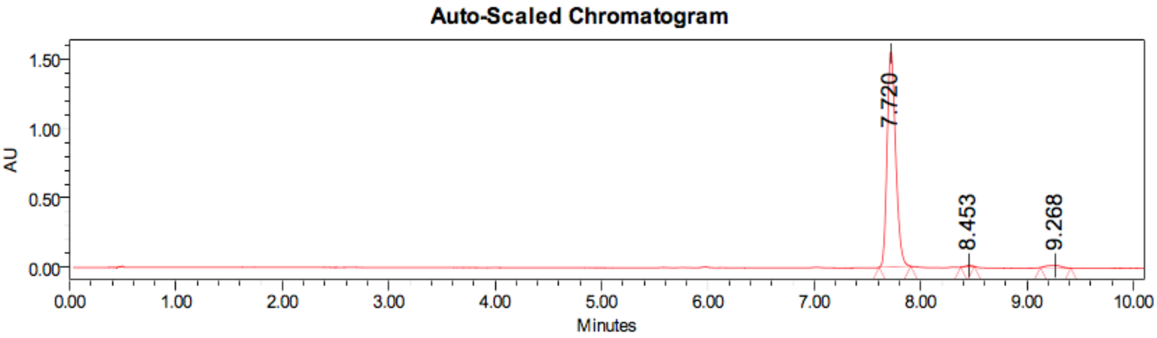

Processed Channel: PDA 254.0 nm

	Processed Channel	Retention Time (min)	Area	% Area	Height
1	PDA 254.0 nm	7.720	8630693	97.76	1551194
2	PDA 254.0 nm	8.453	41366	0.47	10083
3	PDA 254.0 nm	9.268	156625	1.77	17262

Supplementary Tables

Supplementary Table 1. PD analysis coding.

Supplementary Table 2. PD analysis of the effects of QNX-sLXms on NF-κB activity.

Supplementary Table 3. PD analysis of the effects of QNX-sLXms on cytokine release.

Supplementary Table 4. Safety Index of QNX-sLXms.

Supplementary Table 5. PD analysis of the effects of (*R*)-**6** on macrophage phagocytosis.

Supplementary Table 6. Effect of sLXm (*R*)-**6** on murine carrageenan-induced paw oedema.

Supplementary Table 7. PD analysis of the effects on intracellular calcium mobilization of (*R*)-**6** QNX-sLXm lead.

Supplementary Figures

Supplementary Figure 1. Effect of series (**17**), (**7**), (**8**) of QNX-sLXms on LPS-induced NF-κB-driven luciferase activity in monocytes.

Supplementary Figure 2. Effect of series (**6**) of QNX-sLXms on LPS-induced pro-inflammatory cytokine release in monocytes.

Supplementary Figure 3. Effect of series (**7**) and (**8**) of QNX-sLXms on LPS-induced pro-inflammatory cytokine release in monocytes.

.

Supplementary Figure 4. Intrinsic cytotoxic profile of series (**17**), (**7**), (**8**) of QNX-sLXms.

Supplementary Figure 5. Extrinsic cytotoxic profile of series (**17**), (**7**), (**8**) of QNX-sLXms.

Supplementary Figure 6. Cell model for intracellular calcium flux measurement.

Supplementary Figure 7. Intracellular calcium flux kinetic traces.

Supplementary Tables

RELATIVE "PD Score"		
***Rel. Efficacy* (*I max* or *E max*)**	0.5 < x < 1	-1
	x = 1	0
	1 < x < 1.5	1
	x ≥ 1.5	2
***Rel. Potency* (*IC 50* or *E 50*)**	x < 0.1	-2
	0.1 < x < 1	-1
	x = 1	0
	1 < x < 5	1
	x ≥ 5	2
***Rel. Slope* (*HS*)**	x = 0	-4
	x < 0.01	-2
	0.01 < x < 1	-1
	x = 1	0
	1 < x < 10	1
	x ≥ 10	2

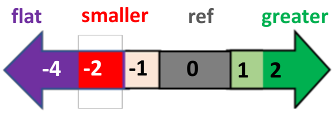

Supplementary Table 1 - PD analysis coding. For the *in vitro* assays, a PD analysis was conducted for the tested molecules to determine the PD profile *per se* and relative to LXA_4 (**1**). The ‘coding’ of the heat-map indicates the arbitrary criteria to assign points to each single PD component [efficacy, potency and slope], in order to generate a final (aggregate) ‘*Relative PD score*’.

Cell Line	Differentiation stage	Assay	Class	Group	Compound	Efficacy		Potency		Slope		(Single component) Rel. Score			*(Aggreagate)*	
						Imax (%)	**Rel. Eff.**	IC50 (nM)	**Rel. Pot.**	HS	**Rel. Slope**	*Rel. Eff.*	*Rel. Pot.*	*Rel. Sl.*	*Rel. PD Score*	
THP-1 Lucia	Monocytes	NFkB activity	*Native*	*Ref*	*1*	*24 ± 1*	*1*	*0.060*	*1*	*-10*	*1*	*ref*	*ref*	*ref*	*ref*	*ref*
			IMZ	Ctr	5	44 ± 9	1.83	0.0002	300	90	-9	2	2	1	5	●●●
			QNX	A	(*R*)-**17**	18 ± 7	**0.75**	0.001	**60**	0	**0**	-1	2	-4	**-3**	
					(*S*)-**17**	19 ± 3	**0.79**	0.003	**24**	0	**0**	-1	2	-4	**-3**	
				B	(*R*)-**6**	38 ± 9	**1.58**	0.025	**2.4**	-566	**57**	2	1	2	**5**	●●●
					(*S*)-**6**	34 ± 9	**1.42**	na	**na**	-4.7	**0.47**	1	na	1	**2**	●●
				C	(*R*)-**7**	29 ± 7	**1.21**	4.000	**0.015**	-5.5	**0.55**	1	-2	-1	**-2**	
					(*S*)-**7**	36 ± 9	**1.50**	1.000	**0.1**	-6	**0.60**	2	-2	-1	**-1**	
				D	(*R*)-**8**	23 ± 7	**0.96**	0.040	**1.5**	-4.9	**0.49**	0	1	-1	**0**	
					(*S*)-**8**	23 ± 8	**0.96**	0.010	**6**	0	**0**	0	2	-4	**-2**	

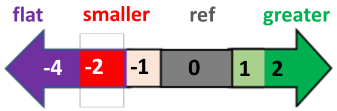

Supplementary Table 2 - PD analysis of the effects of QNX-sLXms on NF-κB activity. THP-1 LUCIA® monocytes were treated with appropriate controls or QNX-sLXms, as above described. The table summarises the effects of QNX-sLXms on LPS-induced NF-κB activity in monocytes. In order to generate an aggregate *PD score*, three PD components were calculated: I_{max}, IC_{50} and HS, as a measure of efficacy, potency and slope, respectively, as absolute values or relative to LXA_4. (Refer to **Supp. Table 1** for heat-map 'coding').

Cell Line	Differentiation stage	Assay	Class	Group	Compound	Efficacy		Potency		Slope		(Single component) Rel. score			*(Aggreagate)* *Rel. PD Score*	
						Imax (%)	**Rel. Eff.**	IC50 (nM)	**Rel. Pot.**	HS	**Rel. Sl.**	*Rel. Eff.*	*Rel. Pot.*	*Rel. Sl.*		
THP-1 Lucia	Monocytes	IL6	*Native*	*Ctr*	*1*	*99 ± 1*	*1*	*0.001*	*1*	*-239*	*1*	*ref*	*ref*	*ref*	*ref*	*ref*
			QNX	B	(*R*)-**6**	84 ± 2	**0.85**	0.001	**1**	-174	**0.73**	-1	0	-1	**-2**	
					(*S*)-**6**	98 ± 1	**0.99**	0.001	**1**	-576	**2.41**	0	0	2	**2**	●
				C	(*R*)-**7**	138 ± 36	**1.39**	0.100	**0.01**	0	**0**	-2	-2	-4	**-8**	
					(*S*)-**7**	19 ± 13	**0.19**	15.000	**0.0001**	0	**0**	-2	-2	-4	**-8**	
				D	(*R*)-**8**	21 ± 4	**0.21**	0.001	**1**	0	**0**	-2	0	-4	**-6**	
					(*S*)-**8**	28 ± 5	**0.28**	0.004	**0.250**	-80	**0.33**	-2	-1	-1	**-4**	
		IL1β	*Native*	*Ctr*	*1*	*95 ± 1*	*1*	*0.001*	*1*	*-188*	*1*	*ref*	*ref*	*ref*	*ref*	*ref*
			QNX	B	(*R*)-**6**	73 ± 2	**0.77**	0.001	**1**	5.5	**-0.03**	-1	0	-1	**-2**	
					(*S*)-**6**	126 ± 42	**1.33**	1.000	**0.001**	0.16	**-0.001**	-2	-2	-2	**-6**	
				C	(*R*)-**7**	67 ± 3	**0.71**	0.002	**0.5**	-90.8	**0.48**	-1	-1	-1	**-3**	
					(*S*)-**7**	54 ± 14	**0.57**	0.002	**0.5**	-95.1	**0.51**	-2	-1	-1	**-4**	
				D	(*R*)-**8**	57 ± 12	**0.60**	0.001	**1**	-90.7	**0.48**	-2	0	-1	**-3**	
					(*S*)-**8**	59 ± 20	**0.62**	0.001	**1**	-95.1	**0.51**	-2	0	-1	**-3**	
		INFγ	*Native*	*Ctr*	*1*	*72 ± 4*	*1*	*0.001*	*1*	*-448*	*1*	*ref*	*ref*	*ref*	*ref*	*ref*
			QNX	B	(*R*)-**6**	62 ± 1	**0.86**	0.001	**1**	-494	**1**	-1	0	0	**-1**	
					(*S*)-**6**	74 ± 4	**1.03**	1.800	**0.001**	-0.6	**0.001**	0	-2	-2	**-4**	
				C	(*R*)-**7**	90 ± 2	**1.25**	0.001	**1**	-431	**1**	1	0	0	**1**	●
					(*S*)-**7**	85 ± 4	**1.18**	0.002	**0.500**	-0.23	**0.001**	1	-1	-2	**-2**	
				D	(*R*)-**8**	60 ± 8	**0.83**	0.001	**1**	-457	**1**	-1	0	0	**-1**	
					(*S*)-**8**	61 ± 8	**0.85**	0.001	**1**	-97	**0.22**	-1	0	-1	**-2**	

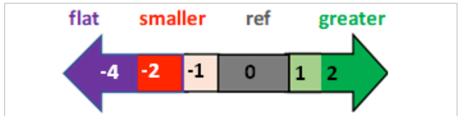

Supplementary Table 3 - PD analysis of the effects of QNX-sLXms on cytokine release. THP-1 LUCIA® monocytes were treated with appropriate controls or QNX-sLXms, as above described. The tables summarise the effects of QNX-sLXms on LPS-induced release of pivotal pro-inflammatory cytokines in monocytes. In order to generate an aggregate PD score, three pharmacodynamic components were calculated: observed or predicted I_{max}; IC_{50} and HS, as a measure, respectively, of efficacy, potency and slope, as absolute values or relative to LXA_4. (Refer to **Supp. Table 1** for the heat-map 'coding').

		SAFETY PROFILE			
		Extrinsic Activity	Intrinsic Toxicity		*Safety Index*
		hIC50 (pM)	TC50 (pM)	Level	*TC 50 /IC 50*
**Group A**	(*R*)-**17**	10	100	Moderate	***10***
	(*S*)-**17**	3	100	Moderate	***33***
**Group B**	(*R*)-**6**	25	500	Low	***20***
	(*S*)-**6**	20	200	Low-Mod.	***10***
**Group C**	(*R*)-**7**	100	2,000	Low-Mod.	***20***
	(*S*)-**7**	15,000	110,000	Low-Mod.	***7***
**Group D**	(*R*)-**8**	200	10,000	Low-Mod.	***50***
	(*S*)-**8**	10	100	Low	***10***

Supplementary Table 4 - Safety Index of QNX-sLXms. The table displays the ***Safety index*** (S_i) calculated for each tested sLXm, as the ratio between the half-maximal *intrinsic LDH-associated* toxic activity (TC_{50}) and the half-maximal *extrinsic* biological activity of the same compound (the highest IC_{50} among the various LPS-challenged assays, as a measure of anti-inflammatory activity).

Cell Line	Differentiation stage	Assay	Class	Group	Compound	Efficacy		Potency		Single comp. score		*Aggreagate*	
						Emax (%)	**Rel. Eff.**	EC50 (nM)	**Rel. Pot.**	***Rel Eff.***	***Rel. Pot.***	***Rel. PD Score***	
**THP-1**	**Mf0 Macrophage**	**Phagocytosis**	**SPMs**	***LXs***	***1***	*4.2 ± 0.5*	***0***	5	**1**	***ref***	***ref***	***ref***	***ref***
					**LXB4**	2.2 ± 0.4	**0.00**	5	**1**	-2	0	**-2**	
				Rvs	**RvD1**	3.6 ± 0.7	**0.00**	0.0005	**10000**	-1	2	**1**	●
					**RvE1**	1.7 ± 0.2	**0.00**	0.0010	**5000**	-2	2	**0**	
			**sLXms**	**IMZ**	**5**	4.2 ± 0.7	**0.00**	0.010	**500**	0	2	**2**	●●
				**QNX**	(*R*)-**6**	3.4 ± 1.3	**0.00**	0.050	**100**	-1	2	**1**	●

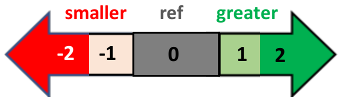

Supplementary Table 5 - PD analysis of the effects of (*R*)-6 on macrophage phagocytosis. PMA-triggered THP-1-derived-MF0 macrophages were treated with appropriate controls or sLXms, as above described. The tables summarise the effects of tested molecules on macrophage phagocytosis. In order to generate an aggregate *PD score*, three PD components were calculated: observed or predicted I_{max}; IC_{50} and *Hill Slope (HS)*, as a measure, respectively, of *efficacy*, *potency* and *slope*, as absolute values or relative to LXA_4. (Refer to **Supp. Table 1** for *heat-map 'coding')*.

	% relative to carageenan-induced level							
	Veh		Naproxen		(*R*)-**6**		(*R*)-**17**	
Time	%	% reduction	%	% reduction	%	% reduction	%	% reduction
4h	***100***	0	**118**	18	**116**	16	**103**	3
8h	***100***	0	**40**	-60	**63**	-37	**84**	-16
24h	***100***	0	**55**	-45	**72**	-28	**92**	-8
48h	***100***	0	**160**	60	**53**	-47	**149**	49
72h	***100***	0	**550**	450	**110**	10	**220**	120

Supplementary Table 6 - Effect of sLXm *(R)*-6 on murine carrageenan-induced paw oedema. *(R)*-**6**, *(R)*-**17** (2 µg / kg) or Naproxen (50 mg / kg, p.o.) were administered 30 min before the intra-paw injection of 1% carrageenan into male C57bl/6 mice. Paw swelling was monitored over time using an external lever gauge. (**a**) Graph shows paw-oedema index. 1-way ANOVA statistical analysis has been performed * = $p < 0.05$, ** = $p < 0.01$, *** = $p < 0.001$. (**b**) Table displays the index of each tested molecule relative to the carrageenan-induced levels. Data are presented as mean ± SEM, n=3 mice/treatment group.

Cell Line	Transfection	Assay	Class	Group	Compound	Efficacy		Potency		Slope		(Single component) Rel. Score			*(Aggreagate)*	
						Emax (%)	**Rel. Eff.**	E50 (nM)	**Rel. Pot.**	HS	**Rel. Slope**	***Rel. Eff.***	***Rel. Pot.***	***Rel. Sl.***	***Rel. PD Score***	
**HEK-293**	**FPR2+/Gαq+**	**ALX/FPR2**	***Native***	***Ref***	***1***	*100*	***1***	*0.0012*	***1***	*150*	***1***	***ref***	***ref***	***ref***	***ref***	***ref***
		**receptor**	**IMZ**	**ctr**	**5**	99 ± 17	**0.99**	0.0012	**1**	258	***1.72***	0	0	2	**2**	●
		**activation**	**QNX**	**B**	(*R*)-**6**	61 ± 10	**0.61**	0.0012	**1**	220	***1.46***	-2	0	1	**-1**	

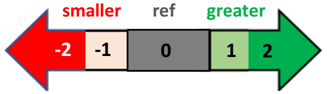

Supplementary Table 7. PD analysis of the effects on intracellular calcium mobilization of (*R*)-6 QNX-sLXm lead. Stably double transfected FPR2^{+}/Gα_q^{+} HEK-293 were treated with appropriate controls or QNX-sLXm lead, as above described. The tables summarise the effects of tested molecules on intracellular calcium flux. In order to generate an aggregate *PD score*, three PD components were calculated: observed or predicted E_{max}; EC_{50} and *HS*, as a measure, respectively, of *efficacy*, *potency* and *slope*, as absolute values or relative to LXA_4. (Refer to **Supp. Table 1** for *heat-map 'coding')*.

Supplementary Figures

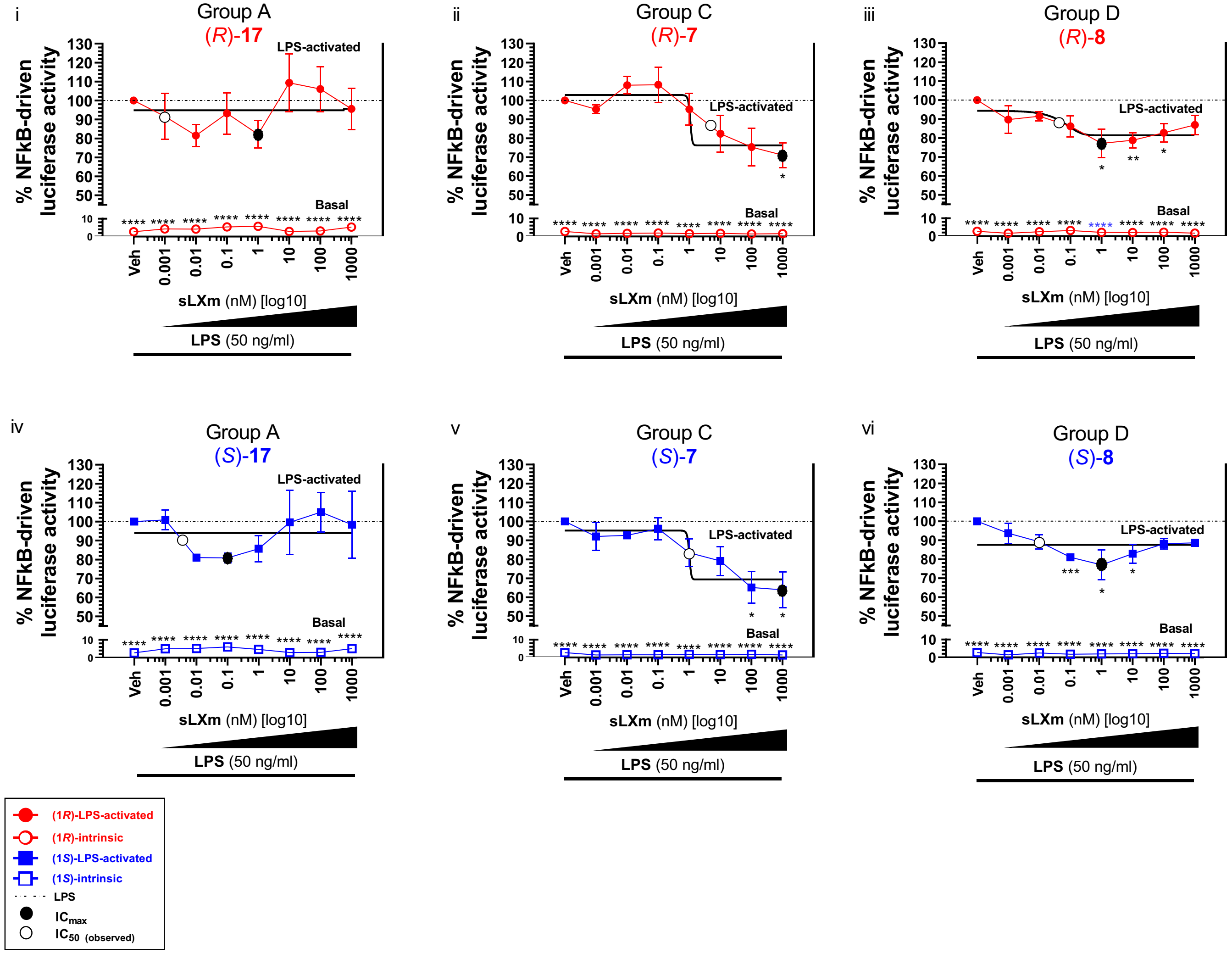

Supplementary Figure 1 - Effect of series (17), (7), (8) of QNX-sLXms on LPS-induced NF-κB-driven luciferase activity in monocytes. $1x10^5$ THP-1 LUCIA® monocytes were pre-treated for 30 min with sLXms; vehicle or appropriate controls, at indicated concentrations in the presence (LPS-activated) or absence (basal) of 50 ng/mL of LPS. After 24 h supernatants were collected and NF-κB-driven luciferase activity assayed. Concentration-response curves of QNX-sLXms from group A, C and D are here displayed. Data are expressed as % ± SEM (n=3) of Normalised Luminescence Unit relative to LPS-induced response. Best fitting curves are indicated by black solid lines. Statistical analysis was carried out by using Student's unpaired 2-tailed T-test of tested compound *vs* LPS (* = $p<0.05$; ** = $p< 0.01$; *** $p < 0.001$; **** $p < 0.0001$) or *vs* LXA_4 **1** (not shown).

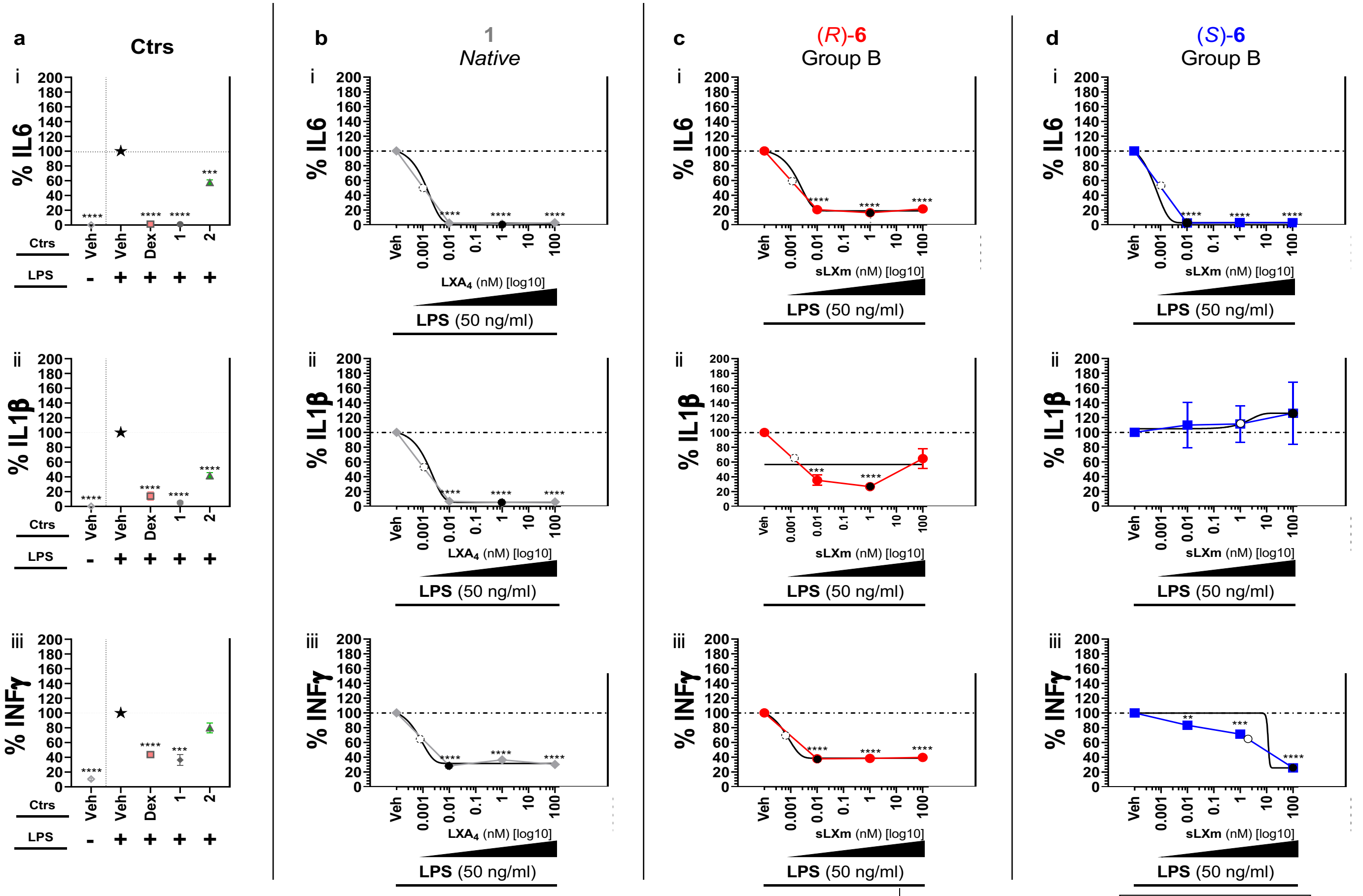

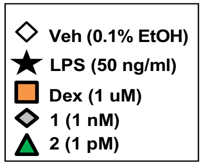

Cytokine	LPS-induced release (pg/ml)
IL6	3584 ± 122
IL1β	1162 ± 53
INFγ	145 ± 11

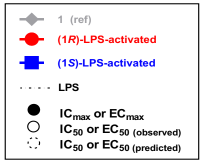

Supplementary Figure 2. Effect of series (6) of QNX-sLXms on LPS-induced pro-inflammatory cytokine release by monocytes. $1x10^5$ THP-1 LUCIA® monocytes were pre-treated for 30 min with sLXms; vehicle or appropriate controls, at indicated concentrations, challenged with 50 ng/mL of LPS. After 24 h supernatants were collected and a panel of 7 pro-inflammatory cytokine levels were assayed: IL-6, IL-1β and IFN-γ are shown. (**a**) Single point analysis of the internal controls. (**b**) Concentration-response curves of reference [**1**] and QNX-sLXms from group B are shown. Data are expressed as % cytokine secretion relative to LPS alone ± SEM (n=3). Best fitting curves are indicated by black solid lines. Statistical analysis was carried out by using Student's unpaired 2-tailed T-test of tested compound *vs* LPS (* = p<0.05; ** = p< 0.01; *** p < 0.001; **** p < 0.0001) or *vs* LXA_4 **1** (not shown).

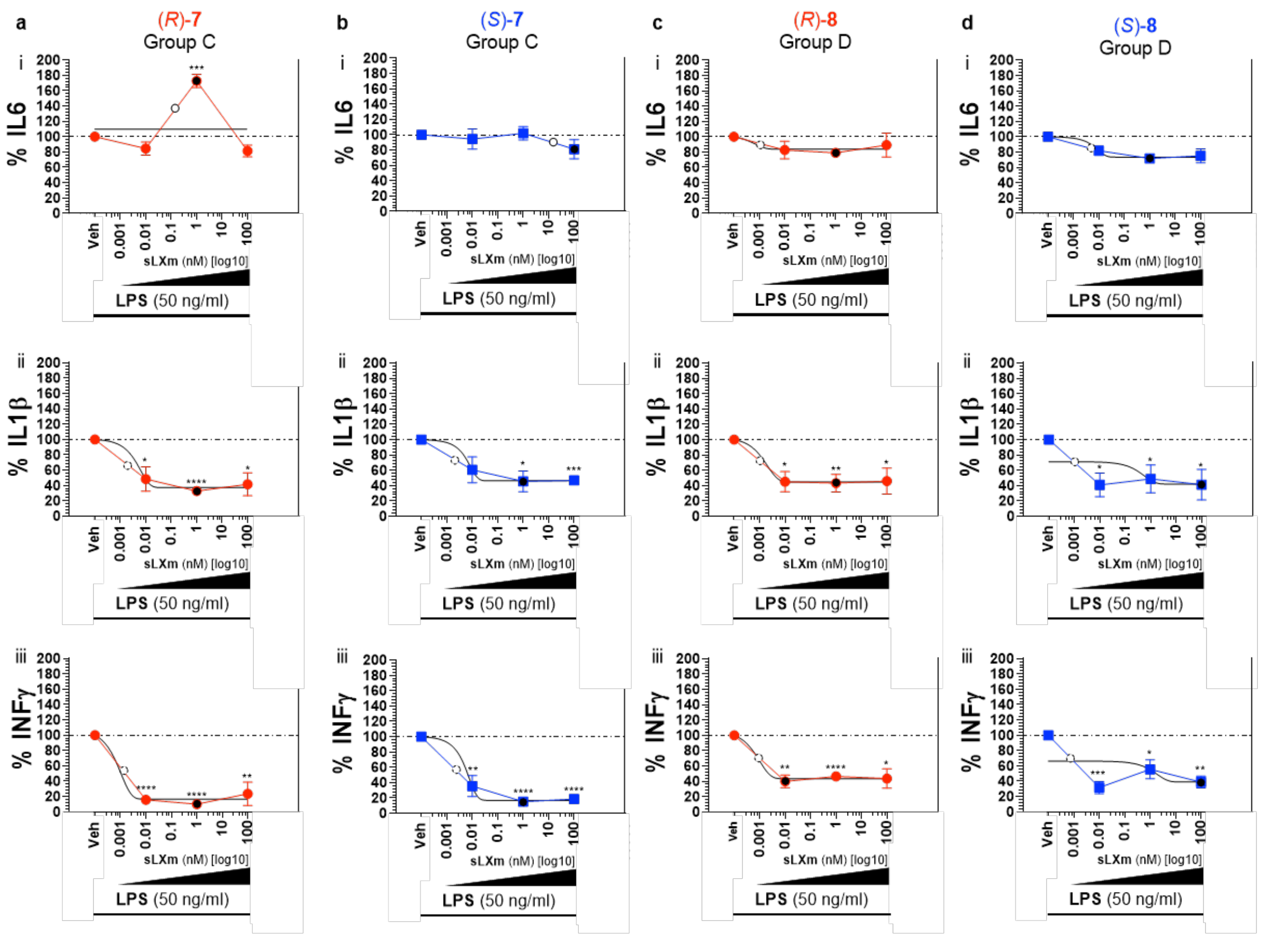

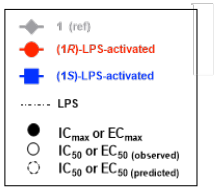

Cytokine	LPS-induced release (pg/ml)
IL6	3584 ± 122
IL1β	1162 ± 53
INFγ	145 ± 11

Supplementary Figure 3. Effect of series (7) and (8) of QNX-sLXms on LPS-induced pro-inflammatory cytokine release in monocytes. $1x10^5$ THP-1 LUCIA® monocytes were pre-treated for 30 min with sLXms; vehicle or appropriate controls, at indicated concentrations, challenged with 50 ng/mL of LPS. After 24 h supernatants were collected and a panel of 7 pro-inflammatory cytokine levels were assayed: IL-6, IL-1β and IFN-γ are shown. Concentration-response curves of QNX-sLXms from group C and D are displayed. Data are expressed as % cytokine secretion relative to LPS alone ± SEM (n=3). Best fitting curves are indicated by black solid lines. Statistical analysis was carried out by using Student's unpaired 2-tailed T-test of tested compound *vs* LPS (* = $p<0.05$; ** = $p< 0.01$; *** $p < 0.001$; **** $p < 0.0001$) or *vs* LXA_4 **1** (not shown).

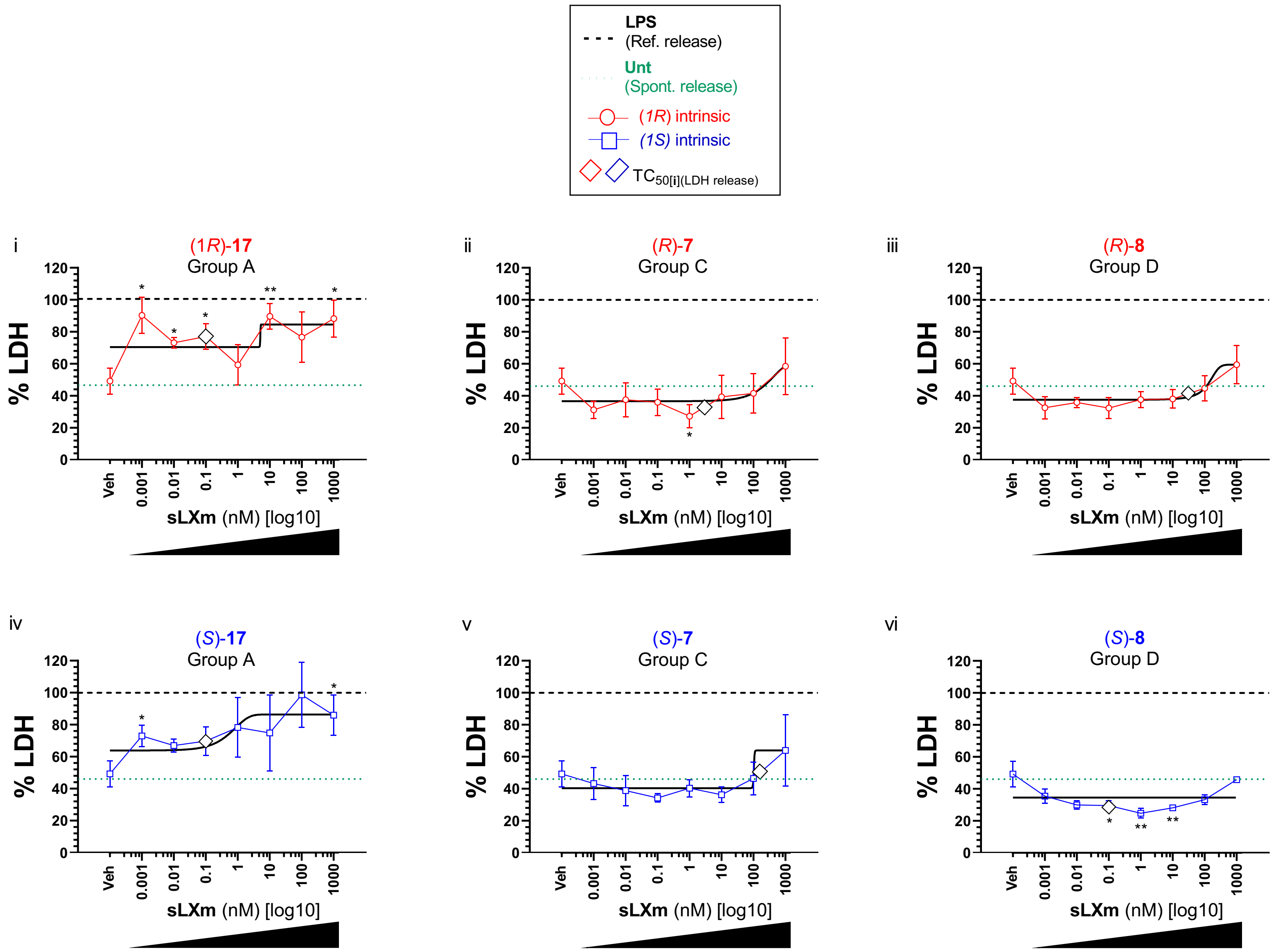

LPS
(Ref. release)
Unt
(Spont. release)
(1R) intrinsic
(1S) intrinsic
TC50[i](LDH release)
i
(1R)-17
Group A
ii
(R)-7
Group C
iii
(R)-8
Group D
iv
(S)-17
Group A
v
(S)-7
Group C
vi
(S)-8
Group D
% LDH
sLXm (nM) [log10]
Veh
0.001
0.01
0.1
1
10
100
1000

Supplementary Figure 4 - Intrinsic cytotoxic profile of series (17), (7), (8) of QNX-sLXms. $1x10^5$ THP-1 LUCIA® monocytes were treated for 24 h with QNX-sLXms, vehicle or appropriate controls [1 pM - 1 μM]. After 24 h, supernatants were collected and LDH release assayed. Concentration-response and best-fitting curves of **(17)**, **(7)**, **(8)** are shown. Data are expressed as % LDH release relative to LPS ± SEM (n=3). Statistical analysis was carried out by using Student's unpaired 2-tailed T-test of tested compound *vs* LPS (* = $p<0.05$; ** = $p< 0.01$).

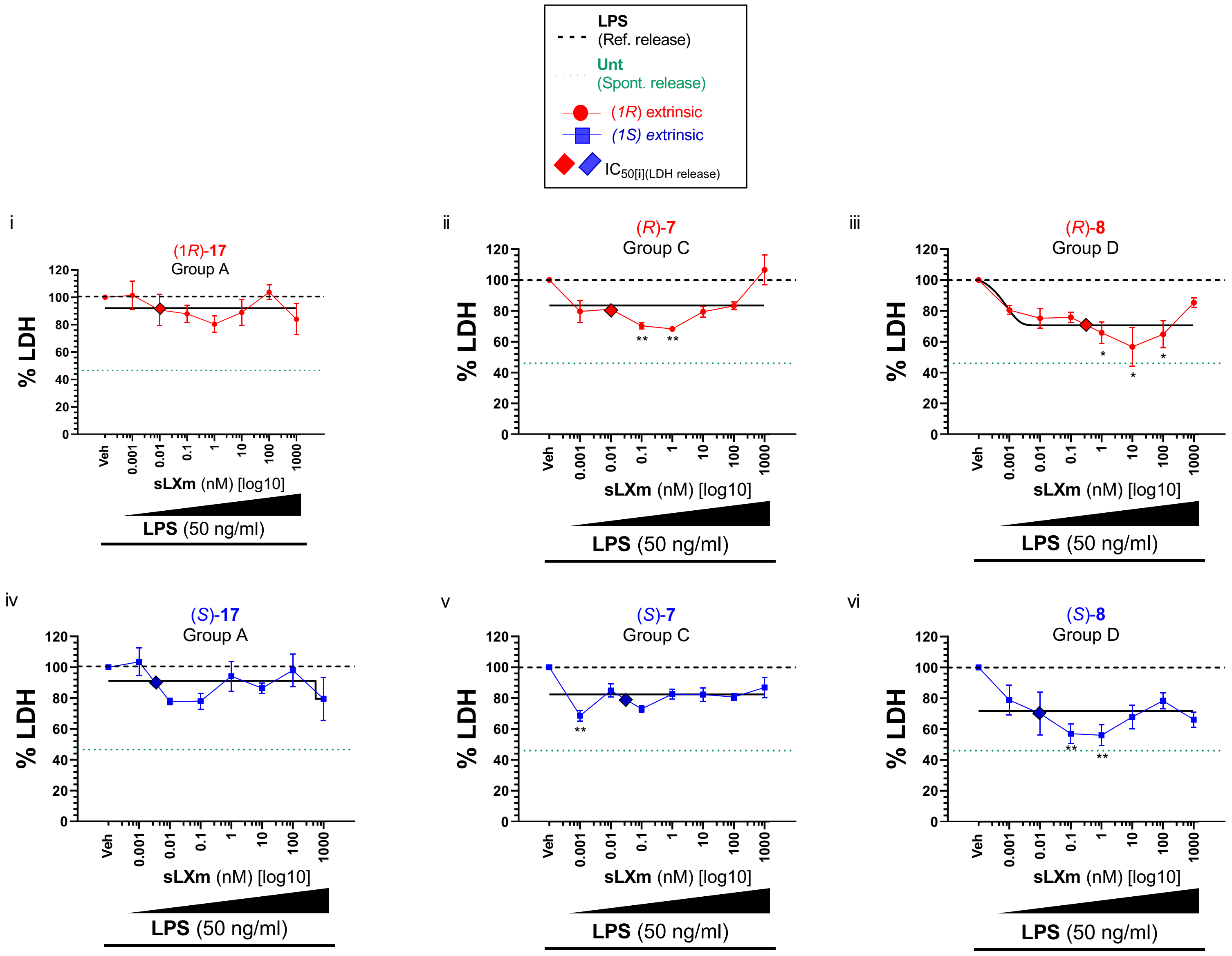
LPS
(Ref. release)
Unt
(Spont. release)
(1R) extrinsic
(1S) extrinsic
IC50[i](LDH release)
i
(1R)-17
Group A
ii
(R)-7
Group C
iii
(R)-8
Group D
iv
(S)-17
Group A
v
(S)-7
Group C
vi
(S)-8
Group D
% LDH
Veh
0.001
0.01
0.1
1
10
100
1000
sLXm (nM) [log10]
LPS (50 ng/ml)

Supplementary Figure 5 - Extrinsic cytotoxic profile of series (17), (7), (8) of QNX-sLXms. $1x10^5$ THP-1 LUCIA® monocytes pre-treated for 30 mins with QNX-sLXms, vehicle or appropriate controls, at increasing doses ranging (1 pM - 1 μM), and subsequently challenged for 24 hrs with 50 ng/ml LPS. After 24 h supernatants were collected and LDH release assayed. Concentration-response and best-fitting curves of **(17)**, **(7)**, **(8)** are shown. Data are expressed as % LDH release relative to LPS ± SEM (n=3). Statistical analysis was carried out by using Student's unpaired 2-tailed T-test of tested compound *vs* LPS (* = $p<0.05$; ** = $p< 0.01$; *** $p < 0.001$; **** $p < 0.0001$).

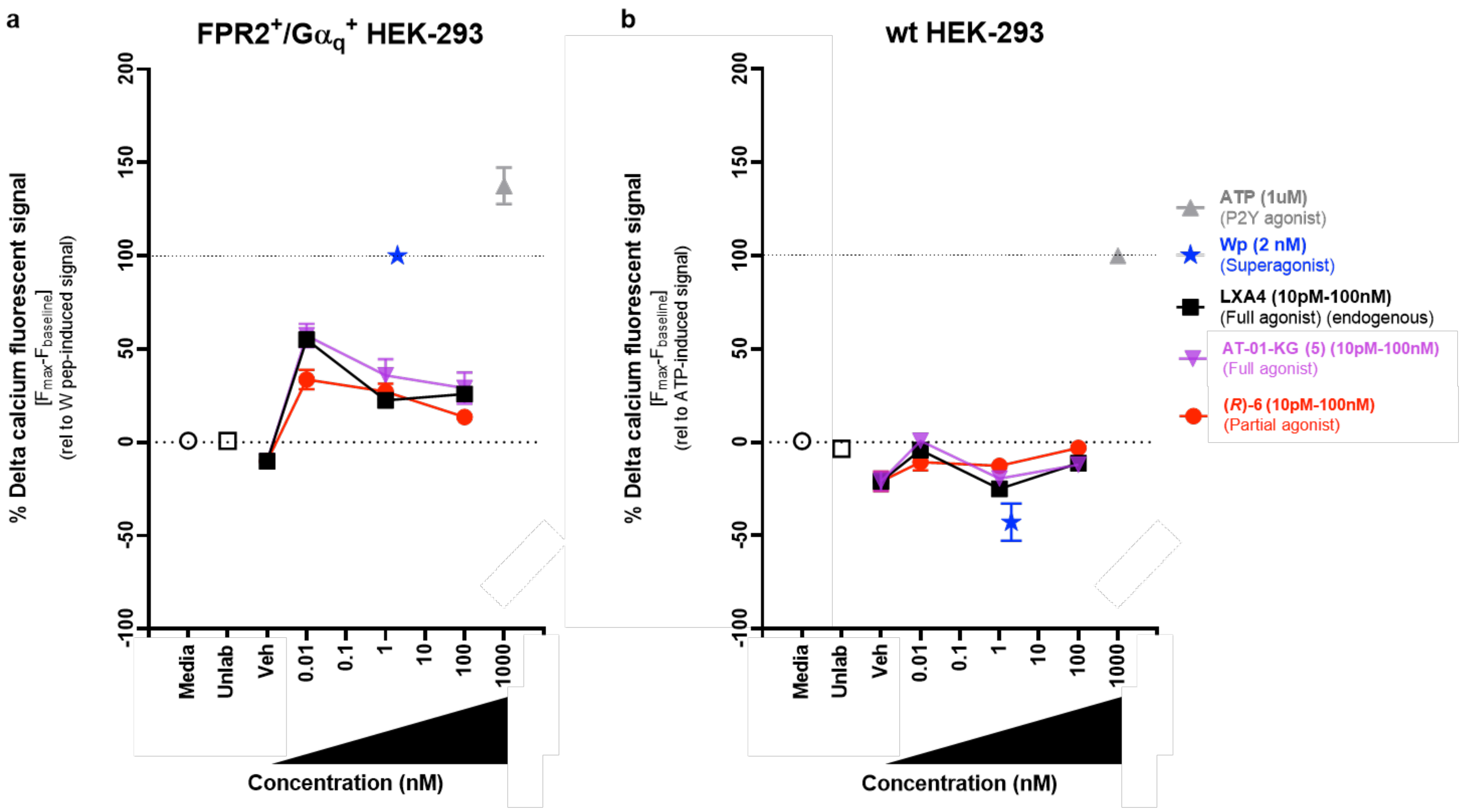
a
FPR2+/Gαq+ HEK-293
b
wt HEK-293
% Delta calcium fluorescent signal
[Fmax-Fbaseline]
(rel to W pep-induced signal)
(rel to ATP-induced signal)
200
150
100
50
0
-50
-100
Media
Unlab
Veh
0.01
0.1
1
10
100
1000
Concentration (nM)
ATP (1uM)
(P2Y agonist)
Wp (2 nM)
(Superagonist)
LXA4 (10pM-100nM)
(Full agonist) (endogenous)
AT-01-KG (5) (10pM-100nM)
(Full agonist)
(R)-6 (10pM-100nM)
(Partial agonist)

Supplementary Figure 6 - Cell model for intracellular calcium flux measurement. Intracellular calcium mobilization was assessed in (**a**) stably transfected cell line overexpressing ALX/FPR2 and $G\alpha_q$ as well as (**b**) in wt system. ATP (1 mM) and Wp (2 nM) were used as controls. Quantification of fluorescent signal was carried out by calculating differential calcium signals at baseline and at peak. Data are expressed as % delta calcium peak relative to the known full agonist (**1**) ± SEM (n=3). Statistical analyses were carried out by using Student's unpaired T-test of tested compound *vs* veh (not shown).

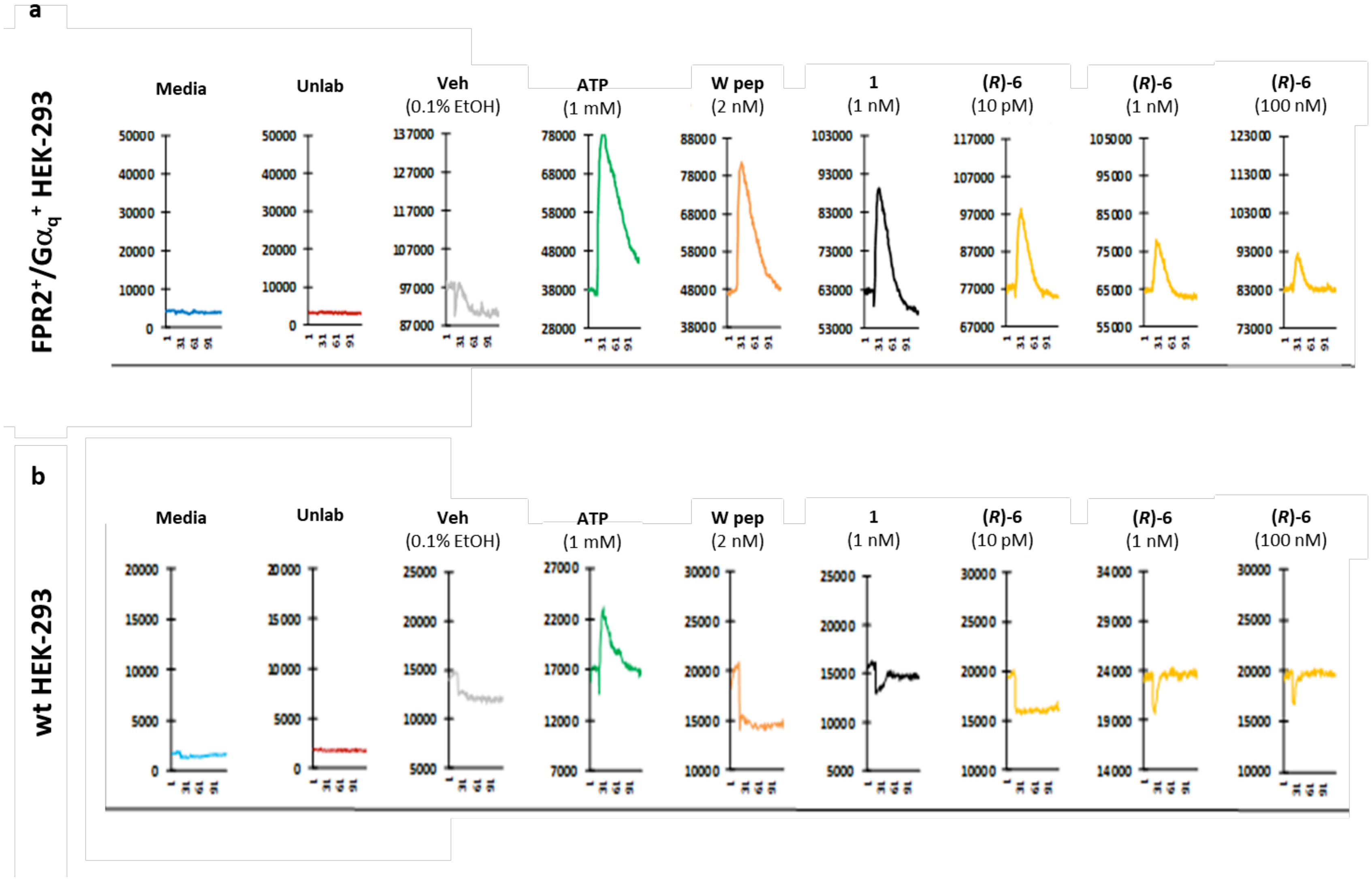
a
FPR2+/Gαq+ HEK-293
Media
Unlab
Veh (0.1% EtOH)
ATP (1 mM)
W pep (2 nM)
1 (1 nM)
(R)-6 (10 pM)
(R)-6 (1 nM)
(R)-6 (100 nM)
b
wt HEK-293
Media
Unlab
Veh (0.1% EtOH)
ATP (1 mM)
W pep (2 nM)
1 (1 nM)
(R)-6 (10 pM)
(R)-6 (1 nM)
(R)-6 (100 nM)

Supplementary Figure 7 - Intracellular calcium flux kinetic traces. Intracellular calcium mobilization was assessed in stably transfected HEK-293 overexpressing ALX/FPR2 and Ga_q (**a**) as well as in the relative wt system (**b**) ATP (P2Y receptor agonist) and Wp (FPR2/ALX agonist) were used as controls. Representative "*baseline + agonist addition*" kinetic steps: the baseline fluorescent signal was measured for 20 s, followed by 100 s immediately after agonist injection. (Subsequent "*Triton + EGTA*" kinetic steps are not shown here). Statistical analyses were carried out by using Student's unpaired T-test of the mimetic and controls not shown).
